# Supplementary material for: Scalarane Sesterterpenoids Isolated from the Marine Sponge Hyrtios erectus and their Cytotoxicity
Source: Mar Drugs. 2022 Sep 25;20(10):604. doi: 10.3390/md20100604 (PMC9605097; doi:10.3390/md20100604)
Supplement: Supplementary file 1 [file marinedrugs-20-00604-s001.zip › marinedrugs-1908112-SI.pdf]

Supplementary Materials

Scalarane Sesterterpenoids Isolated from the Marine Sponge *Hyrtios erectus* and their Cytotoxicity

Huynh Nguyen Khanh Tran [1], Min Jin Kim [1], and Yeon-Ju Lee [1, 2, *]

[1] Marine Natural Products Chemistry Laboratory, Korea Institute of Ocean Science and Technology, 385 Haeyangro, Busan 49111, Republic of Korea

[2] Department of Applied Ocean Science, University of Science and Technology, Daejeon 34113, Republic of Korea

***** Correspondence: yjlee@kiost.ac.kr

List of Tables

Table S.1. Complete NMR data of **1** in $CDCl_3$

Table S.2. Complete NMR data of **2** in $CDCl_3$

Table S.3. Complete NMR data of **3** in C_5D_5N

Table S.4. Complete NMR data of **4** in $CDCl_3$

Table S.5. Complete NMR data of **5** in $CDCl_3$

Table S.6. Complete NMR data of **6** in C_5D_5N

Table S.7. Complete NMR data of **7** in C_5D_5N

Table S.8. Complete NMR data of **8** in C_5D_5N

Table S.9. HRMS analysis results of known compounds (**9**–**18**)

Table S.10. Inhibitory activity of the isolated compounds (**1**−**18**) against cancer cell growth

Table S.11. The cytotoxic effect of isolated compounds (**1**−**18**) on Hela and MCF-7 cancer cell lines at the various concentrations.

List of Figures

Figure S.1.1. ^{1}H NMR spectrum of compound **1** in $CDCl_3$

Figure S.1.2. ^{13}C NMR and DEPT spectra of compound **1** in $CDCl_3$

Figure S.1.3. HSQC spectrum of compound **1** in $CDCl_3$

Figure S.1.4. COSY spectrum of compound **1** in $CDCl_3$

Figure S.1.5. HMBC spectrum of compound **1** in $CDCl_3$

Figure S.1.6. ROESY spectrum of compound **1** in $CDCl_3$

Figure S.1.7. 1D NOESY spectrum of compound **1** in $CDCl_3$

Figure S.1.8. HR-ESIMS spectrum of compound **1**

Figure S.2.1. ^{1}H NMR spectrum of compound **2** in $CDCl_3$

Figure S.2.2. Comparison of ^{1}H NMR spectra between compound **1** and **2** in $CDCl_3$

Figure S.2.3. ^{13}C NMR and DEPT spectra of compound **2** in $CDCl_3$

Figure S.2.4. HSQC spectrum of compound **2** in $CDCl_3$

Figure S.2.5. COSY spectrum of compound **2** in $CDCl_3$

Figure S.2.6. HMBC spectrum of compound **2** in $CDCl_3$

Figure S.2.7. ROESY spectrum of compound **2** in $CDCl_3$

Figure S.2.8. HR-ESIMS spectrum of compound **2**

Figure S.3.1. ^{1}H NMR spectrum of compound **3** in C_5D_5N

Figure S.3.2. ^{13}C NMR and DEPT spectra of compound **3** in C_5D_5N

Figure S.3.3. HSQC spectrum of compound **3** in C_5D_5N

Figure S.3.4. COSY spectrum of compound **3** in C_5D_5N

Figure S.3.5. HMBC spectrum of compound **3** in C_5D_5N

Figure S.3.6. ROESY spectrum of compound **3** in C_5D_5N

Figure S.3.7. HR-ESIMS spectrum of compound **3**

Figure S.4.1. ^{1}H NMR spectrum of compound **4** in $CDCl_3$

Figure S.4.2. ^{13}C NMR and DEPT spectra of compound **4** in $CDCl_3$

Figure S.4.3. HSQC spectrum of compound **4** in $CDCl_3$

Figure S.4.4. COSY spectrum of compound **4** in $CDCl_3$

Figure S.4.5. HMBC spectrum of compound **4** in $CDCl_3$

Figure S.4.6. ROESY spectrum of compound **4** in $CDCl_3$

Figure S.4.7. HR-ESIMS spectrum of compound **4**

Figure S.5.1. ^{1}H NMR spectrum of compound **5** in $CDCl_3$

Figure S.5.2. Comparison of ^{1}H NMR spectra between compound **4** and **5** in $CDCl_3$

Figure S.5.3. ^{13}C NMR and DEPT spectra of compound **5** in $CDCl_3$

Figure S.5.4. HSQC spectrum of compound **5** in $CDCl_3$

Figure S.5.5. COSY spectrum of compound **5** in $CDCl_3$

Figure S.5.6. HMBC spectrum of compound **5** in $CDCl_3$

Figure S.5.7. ROESY spectrum of compound **5** in $CDCl_3$

Figure S.5.8. HR-ESIMS spectrum of compound **5**

Figure S.6.1. ^{1}H NMR spectrum of compound **6** in C_5D_5N

Figure S.6.2. ^{13}C NMR and DEPT spectra of compound **6** in C_5D_5N

Figure S.6.3. HSQC spectrum of compound **6** in C_5D_5N

Figure S.6.4. COSY spectrum of compound **6** in C_5D_5N

Figure S.6.5. HMBC spectrum of compound **6** in C_5D_5N

Figure S.6.6. ROESY spectrum of compound **6** in C_5D_5N

Figure S.6.7. HR-ESIMS spectrum of compound **6**

Figure S.7.1. ^{1}H NMR spectrum of compound **7** in C_5D_5N

Figure S.7.2. ^{13}C NMR and DEPT spectra of compound **7** in C_5D_5N

Figure S.7.3. HSQC spectrum of compound **7** in C_5D_5N

Figure S.7.4. COSY spectrum of compound **7** in C_5D_5N

Figure S.7.5. HMBC spectrum of compound **7** in C_5D_5N

Figure S.7.6. ROESY spectrum of compound **7** in C_5D_5N

Figure S.7.7. HR-ESIMS spectrum of compound **7**

Figure S.8.1. ^{1}H NMR spectrum of compound **8** in C_5D_5N

Figure S.8.2. ^{13}C NMR and DEPT spectra of compound **8** in C_5D_5N

Figure S.8.3. HSQC spectrum of compound **8** in C_5D_5N

Figure S.8.4. COSY spectrum of compound **8** in C_5D_5N

Figure S.8.5. HMBC spectrum of compound **8** in C_5D_5N

Figure S.8.6. ROESY spectrum of compound **8** in C_5D_5N

Figure S.8.7. HR-ESIMS spectrum of compound **8**

Figure S.9. A photo of *Hyrtios erectus* used in this research

Table S.1. Complete NMR data of **1** in $CDCl_3$

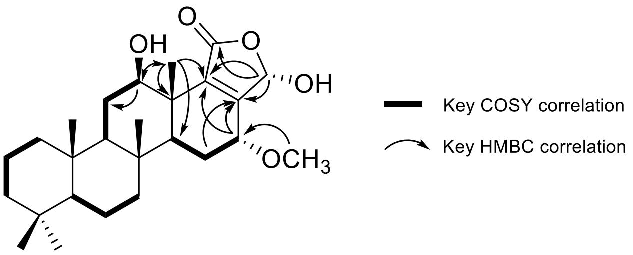

Position	δ_C (ppm)	δ_H (mult, *J* in Hz)	HMBC	COSY
1	39.7	*β* 1.65, m<br>*α* 0.70, m[c]	56.6, 42.1, 37.4, 18.2	1.53, 1.36, 0.70<br>1.65, 1.53
2	18.2	*β* 1.53, m[c]<br>*α* 1.36, m[c]		1.36, 1.06, 0.70<br>1.65, 1.53, 1.06
3	42.1	*β* 1.30, m<br>*α* 1.06, m	56.6, 33.3, 18.2	1.06<br>1.53, 1.36, 1.30
4	33.3			
5	56.6	0.70, m[c]		1.53, 1.36
6	18.6	*β* 1.53, m[c]<br>*α* 1.36, m[c]	56.6	1.76, 1.36, 0.70<br>1.53, 0.70
7	42.7	*β* 1.76, m[c]<br>*α* 0.86, m		1.53, 0.86<br>1.76
8	36.8			
9	57.8	0.84, m[c]		1.76, 1.42
10	37.4			
11	25.5	*β* 1.76, m[c]<br>*α* 1.42, m	75.0, 57.8	3.61, 1.42, 0.84<br>3.61, 1.76, 0.84
12	75.0	3.61, dd (10.8, 4.2)	25.5, 14.7	1.76, 1.42
13	41.4			
14	49.7	1.22, m		1.54
15	21.7	*α* 2.06, d (14.4)<br>*β* 1.54, m	158.3, 69.7, 49.7, 41.4	1.54<br>3.98, 2.06, 1.22
16	69.7	3.98, d (4.2)	158.3, 140.8, 49.7	1.54
17	158.3			
18	140.8			
19	173.7			
20	97.7	6.06, s	173.7, 158.3, 140.8	
21	33.3	0.78, s	56.6, 42.1, 33.3, 21.3	
22	21.3	0.75, s	56.6, 42.1, 33.3	
23	15.9	0.78, s	57.8, 56.6, 39.7, 37.4	
24	17.2	0.83, s	57.8, 49.7, 42.7, 36.8	
25	14.7	1.02, s	140.8, 75.0, 49.7, 41.4	
16-OCH_3	57.4	3.35, s	69.7	

[c]Overlapped with other signals.

Table S.2. Complete NMR data of **2** in $CDCl_3$

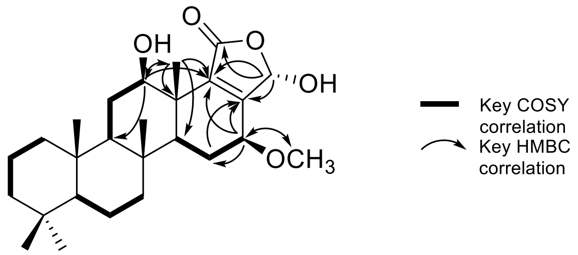

Position	δ_C (ppm)	δ_H (mult, *J* in Hz)	HMBC	COSY
1	40.0	*β* 1.72, m<br>*α* 0.77, m[c]		1.59, 1.42, 0.77<br>1.72, 1.59, 1.42
2	18.7	*β* 1.59, m[c]<br>*α* 1.42, m[c]		1.72, 1.42, 1.09, 0.77<br>1.72, 1.59, 1.09, 0.77
3	42.3	*β* 1.38, d (13.8)<br>*α* 1.09, m[c]		1.09<br>1.59, 1.42, 1.38
4	33.4			
5	56.9	0.77, m[c]		1.73, 1.59, 1.42
6	18.3	*β* 1.59, m[c]<br>*α* 1.42, m[c]		1.42, 0.91, 0.77<br>1.59, 0.91,0.77
7	41.8	*β* 1.73, m<br>*α* 0.91, m		0.77<br>1.59, 1.42
8	37.6			
9	58.1	0.85, m		1.49
10	37.3			
11	25.6	*β* 1.83, m<br>*α* 1.49, m	75.2, 58.1, 42.8, 37.6	3.60, 1.49<br>3.60, 1.83, 0.85
12	75.2	3.60, dd (10.8, 4.2)	140.0, 58.1, 16.5	1.83, 1.49
13	42.8			
14	54.0	1.09, m[c]		1.53
15	23.6	*α* 2.30, dd (12.0, 7.2)<br>*β* 1.53, m	160.0, 74.5, 54.0, 42.8	4.10, 1.53<br>4.10, 2.30, 1.09
16	74.5	4.10, dd (10.2, 7.2)	160.0, 140.0, 57.7, 23.6	2.30, 1.53
17	160.0			
18	140.0			
19	172.7			
20	96.9	6.15, s	172.7, 160.0, 140.0	
21	33.5	0.84, s[c]	56.9, 42.3, 33.4, 21.4	
22	21.4	0.81, s	56.9, 42.3, 33.5, 33.4	
23	16.1	0.84, s[c]	58.1, 56.9, 40.0, 37.3	
24	17.5	0.90, s	58.1, 54.0, 41.8, 37.6	
25	16.5	1.15, s	140.0, 75.2, 54.0, 42.8	
16-OCH_3	57.7	3.47, s	74.5	

[c]Overlapped with other signals.

Table S.3. Complete NMR data of **3** in C_5D_5N

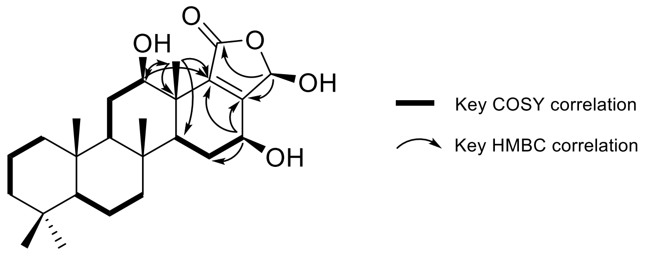

Position	δ_C (ppm)	δ_H (mult, *J* in Hz)	HMBC	COSY
1	40.3	*β* 1.61, d (13.2)		0.73
		α 0.73, m		1.61, 1.54
2	19.3	*β* 1.54, m		1.35, 1.33, 1.13, 0.73
		α 1.35, m[c]		1.54
3	42.8	*β* 1.33, m[c]		1.54, 1.13
		α 1.13, dt (13.8, 4.2)		1.54, 1.33
4	33.8			
5	57.1	0.71, m		1.44, 1.28
6	18.9	*β* 1.44, m		1.68, 1.28, 0.71, 0.63
		α 1.28, m		1.68, 1.44, 0.71, 0.63
7	41.9	*β* 1.68, d (12.6)		1.44, 1.28, 0.63
		α 0.63, m[c]		1.68, 1.44, 1.28
8	37.6			
9	58.2	0.65, d (12.6)		1.62
10	37.9			
11	26.6	*β* 1.96, m[c]	76.2, 58.2, 43.7, 37.6	3.65, 1.62
		α 1.62, d (10.8)		3.65, 1.96, 0.65
12	76.2	3.65, dd (10.8, 4.2)	139.2, 17.2	1.96, 1.62
13	43.7			
14	54.9	1.00, d (12.0)	66.4, 43.7, 37.6	1.99
15	28.8	*α* 2.39, dd (12.6, 7.2)	165.3, 66.4, 54.9, 43.7, 37.6	5.12, 1.99
		β 1.99, m[c]		5.12, 2.39, 1.00
16	66.4	5.12, dd (10.2, 7.2)	165.3, 139.2, 28.8, 17.2[e]	2.39, 1.99
17	165.3			
18	139.2			
19	174.8			
20	99.4	6.96, s	174.8, 165.3	
21	33.9	0.87, s	57.1, 42.8, 33.8, 21.8	
22	21.8	0.80, s	57.1, 42.8, 33.9, 33.8	
23	16.5	0.77, s	57.1, 40.3, 37.9	
24	17.8	0.85, s	58.2, 54.9, 41.9, 37.6	
25	17.2	1.34, s	139.2, 76.2, 54.9, 43.7	

[c]Overlapped with other signals.
[e]Long correlation

Table S.4. Complete NMR data of **4** in $CDCl_3$

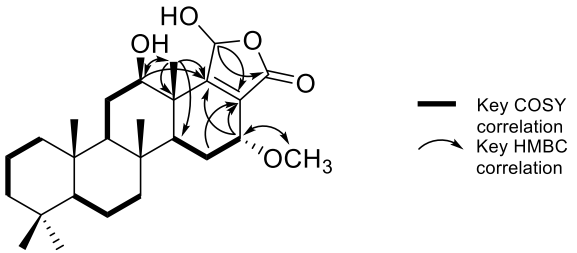

**Position**	**δ_C (ppm)**		**δ_H (mult, *J* in Hz)**		**HMBC**		**COSY**	
	major	minor	major	minor	major	minor	major	minor
1	39.9	39.8	*β* 1.60, m[c]	*β* 1.60, m[c]			1.43	1.43
			α 1.43, m[c]	*α* 1.43, m[c]			1.60	1.60
2	18.3/18.7[d]	18.2/18.7[d]	*β* 1.60, m[c]	*β* 1.60, m[c]			1.43, 1.15	1.43, 1.15
			α 1.43, m[c]	*α* 1.43, m[c]			1.60, 1.15	1.60, 1.15
3	42.2	42.1	*β* 1.37, m	*β* 1.37, m				
			α 1.15, m	*α* 1.15, m			1.60, 1.43	1.60, 1.43
4	33.4	33.2						
5	56.6	58.2	0.80, m[c]	0.80, m[c]			1.68	1.68
6	18.7/18.3[d]	18.7/18.2[d]	*β* 1.68, d (12.6)	*β* 1.68, d (12.6)			1.79, 0.80	1.79, 0.80
			α 0.80, m[c]	*α* 0.80, m[c]			1.68	1.68
7	41.5	41.2	*β* 1.79, d (12.6)	*β* 1.79, d (12.6)			1.68, 0.94	1.68, 0.94
			α 0.94, d (12.6)[c]	*α* 0.94, d (12.6)[c]			1.79	1.79
8	37.6	37.5						
9	58.7	58.0	0.94, d (12.6)[c]	1.02, m			1.82, 1.55	1.82, 1.55
10	37.0	37.1						
11	25.8	26.9	*β* 1.82, m	*β* 1.82, m	74.1, 58.7, 45.3, 37.0	37.1	3.80, 1.55, 0.94	3.66, 1.55, 1.02
			α 1.55, d (12.6)	*α* 1.55, d (12.6)			3.80, 1.82, 0.94	3.66, 1.82, 1.02
12	74.1	75.7	3.80, dd (10.8, 4.5)	3.66, dd (10.8, 4.2)	170.7, 25.8, 15.4	169.7, 13.5	1.82, 1.55	1.82, 1.55
13	45.3	43.6						
14	49.0	49.6	1.44, m	1.39, m			2.06, 1.50	1.63
15	22.5	22.8	*α* 2.06, d (15.0)	1.63, m	126.9, 69.2, 45.3		4.00, 1.50, 1.44	4.32, 1.39
			β 1.50, dd (13.5, 3.9)				4.00, 2.06, 1.44	
16	69.2	69.3	4.00, d (3.6)	4.03, d (3.0)	170.7, 126.9, 57.7, 49.0	128.0, 49.6	2.06, 1.50	1.63
17	126.9	128.0						
18	170.7	169.7						
19	95.9	98.0	6.12, d (1.2)	6.22, s	170.4, 126.9	170.7, 128.0		
20	170.4	170.7						
21	33.4	33.3	0.81, s	0.81, s	56.6, 42.2, 33.4			

22	21.4	21.4	0.85, s[c]	0.85, s[c]	56.6, 33.4	
23	16.3	16.3	0.85, s[c]	0.85, s[c]	58.7, 56.6, 39.9, 37.0	
24	17.9	17.8	0.90, s	0.90, s	58.7, 49.0	
25	15.4	13.5	1.11, s	1.18, s	170.7, 74.1, 49.0, 45.3	169.7, 75.7, 49.6, 43.6
16-OCH_3	57.7	58.2	3.46, s	3.45, s	69.2	

[c]Overlapped with other signals.
[d]Signals may be interchanged.

Table S.5. Complete NMR data of **5** in $CDCl_3$

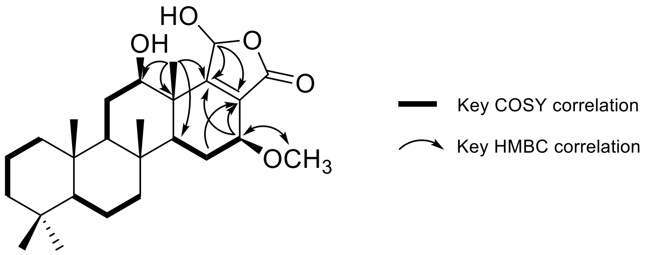

Position	δ_C (ppm)		δ_H (mult, *J* in Hz)		HMBC		COSY	
	major	minor	major	minor	major	minor	major	minor
1	40.0	39.9	*β* 1.68, m	*β* 1.68, m			1.42, 0.78	1.42, 0.78
			α 0.78, m[c]	*α* 0.78, m[c]			1.68	1.68
2	18.7	18.7	*β* 1.57, m[c]	*β* 1.57, m[c]				
			α 1.42, m[c]	*α* 1.42, m[c]			1.68, 1.37	1.68, 1.37
3	42.1	42.0	*β* 1.37, m	*β* 1.37, m			1.42, 1.13	1.42, 1.13
			α 1.13, m	*α* 1.13, m			1.37	1.37
4	33.3	33.3						
5	56.7	56.8	0.78, m[c]	0.78, m[c]			1.42	1.42
6	18.3	18.1	*β* 1.57, m[c]	*β* 1.57, m[c]			1.82, 1.42, 0.90	1.82, 1.42, 0.90
			α 1.42, m[c]	*α* 1.42, m[c]			1.57, 0.78	1.57, 0.78
7	41.5	41.6	*β* 1.82, m	*β* 1.82, m			1.57	1.57
			α 0.90, m	*α* 0.90, m			1.57	1.57
8	37.5	37.5						
9	58.7	58.4	0.87, m [c]	0.87, m [c]			1.79, 1.52	1.82, 1.52
10	37.6	37.5						
11	26.0	26.1	*β* 1.79, m	*β* 1.82, m			3.80, 1.52, 0.87	3.60, 1.52, 0.87
			α 1.52, m	*α* 1.52, m			3.80, 1.79, 0.87	3.60, 1.82, 0.87
12	74.4	75.9	3.80, dd (11.4, 4.2)	3.60, dd (10.2, 3.6)			1.79, 1.52	1.82, 1.52
13	44.7	43.7						
14	53.4	54.7	1.42, m[c]	1.42, m[c]			1.63	1.63
15	24.6	24.5	*α* 2.25, m	*α* 2.25, m	128.6, 73.8, 44.7		4.09	4.09
			β 1.63, m	*β* 1.63, m			4.09, 1.42	4.09, 1.42
16	73.8	73.2	4.09, dd (9.3, 7.5)	4.09, dd (9.3, 7.5)	169.2, 128.6, 58.0		2.25, 1.63	2.25, 1.63
17	128.6	128.6						
18	169.1	170.4						
19	95.4	97.3	6.18, s	6.15, s	169.1, 128.6			
20	169.2	169.1						
21	33.4	33.4	0.84, s[c]	0.84, s[c]	56.7, 42.1, 33.3, 21.4			

22	21.4	21.4	0.81, s	0.80, s	56.7, 42.1, 33.4, 33.3	
23	16.7	16.3	0.84, s[c]	0.84, s[c]	56.7	
24	17.6	17.5	0.91, s	0.93, s	58.7, 53.4, 41.5, 37.5	58.4, 54.7, 41.6, 37.5
25	16.6	15.1	1.21, s	1.30, s	169.1, 74.4, 53.4, 44.7	170.4, 75.9, 54.7, 43.7
16-OCH_3	58.0	58.1	3.54, s	3.56, s	73.8	73.2

[c]Overlapped with other signals.

Table S.6. Complete NMR data of **6** in C_5D_5N

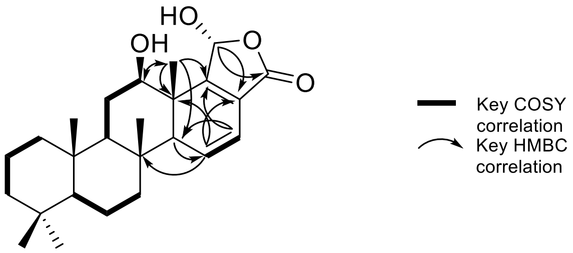

Position	δ_C (ppm)	δ_H (mult, J in Hz)	HMBC	COSY
1	40.2	β 1.55, m[c]		0.66, 1.33
		α 0.66, m		1.55, 1.33
2	19.3	β 1.55, m[c]		1.33, 1.10, 0.66
		α 1.33, m[c]		1.55, 0.66
3	42.7	β 1.33, m[c]		1.55, 1.10
		α 1.10, m		1.55, 1.33
4	33.8			
5	57.0	0.68, m		1.35
6	18.6	β 1.47, m		1.84, 1.35, 0.74
		α 1.35, m		1.84, 1.47, 0.74, 0.68
7	41.3	β 1.84, d (12.6)	57.0	1.47, 1.35, 0.74
		α 0.74, m		1.84, 1.47, 1.35
8	37.9			
9	58.1	0.81, m		1.96
10	33.9			
11	28.1	β 1.96, d (11.4)	72.8, 44.9, 37.9	4.57, 1.74, 0.81
		α 1.74, q (12.0)		4.57, 1.96, 0.81
12	72.8	4.57, dd (11.4, 4.2)	12.5	1.96, 1.74
13	44.9			
14	57.9	2.19, brs	167.3, 133.6, 118.4, 44.9, 37.9, 19.7, 12.5	6.57, 6.11
15	133.6	6.11, dd (9.6, 1.8)	126.3, 57.9, 44.9, 37.9	6.57, 2.19
16	118.4	6.57, dd (9.6, 2.4)	167.3, 57.9	6.11, 2.19
17	126.3			
18	167.3			
19	99.1	7.02, s	170.8, 126.3	
20	170.8			
21	33.8	0.85, s	57.0, 42.7, 33.8, 21.9	
22	21.9	0.80, s[c]	57.0, 42.7, 33.8	
23	16.6	0.80, s[c]	57.0, 40.2, 33.9	
24	19.7	1.01, s	58.1, 57.9, 41.3, 37.9	
25	12.5	1.26, s	167.3, 72.8, 57.9, 44.9	

[c]Overlapped with other signals.

Table S.7. Complete NMR data of **7** in C_5D_5N

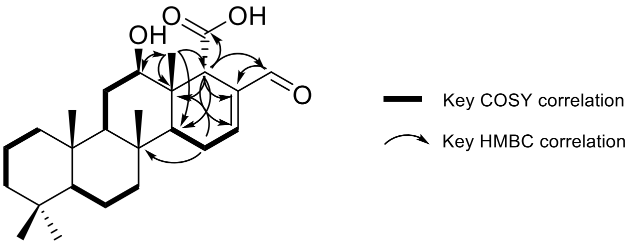

Position	δ_C (ppm)	δ_H (mult, *J* in Hz)	HMBC	COSY
1	41.9	*β* 1.63, d (13.2)<br>*α* 0.89, m		1.40, 0.89<br>1.75, 1.63, 1.40
2	28.1	*β* 1.75, t (11.4)[c]<br>*α* 1.40, m		1.51, 1.40, 0.89<br>1.75, 1.63, 0.89
3	40.1	*β* 1.51, m[c]<br>*α* 0.48, t (9.6)		1.75, 0.48<br>1.51
4	33.7			
5	56.5	0.55, d (12.6)	37.8, 33.7, 22.0	1.28
6	19.3	1.28, m[c]		0.55
7	42.8	1.31, m[c]		
8	38.2			
9	58.7	0.95, m		1.75
10	37.8			
11	28.0	*α* 1.96, dd (11.6, 3.6)<br>*β* 1.75, t (11.4)[c]		4.24<br>4.24, 0.95
12	76.6	4.24, dd (11.4, 3.6)	52.1, 16.8	1.96, 1.75
13	42.5			
14	47.8	2.29, m[c]	42.5, 38.2, 25.4, 16.8	2.48
15	25.4	*α* 2.48, m<br>*β* 2.29, m[c]	42.5, 38.2	7.07, 2.29<br>7.07, 2.48
16	153.7	7.07, m		2.48, 2.29
17	140.1			
18	52.1	*β* 4.27, s	194.4, 176.6, 153.7, 140.1, 47.8, 42.5, 16.8	
19	176.6			
20	194.4	9.71, s	52.1	
21	33.8	0.81, s[c]	56.5, 40.1, 33.7, 22.0	
22	22.0	0.77, s	56.5, 33.8, 33.7	
23	17.3	0.81, s[c]	58.7, 56.5, 37.8	
24	17.6	0.93, s	58.7, 47.8, 42.8, 38.2	
25	16.8	1.13, s	76.6, 52.1, 47.8, 42.5	

[c]Overlapped with other signals.

Table S.8. Complete NMR data of **8** in C_5D_5N

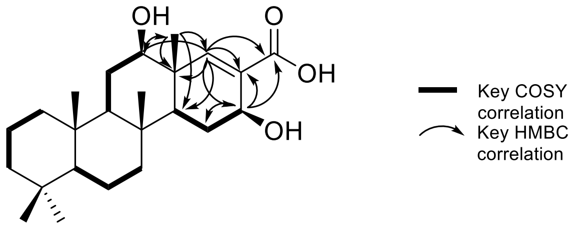

Position	δ_C (ppm)	δ_H (mult, *J* in Hz)	HMBC	COSY
1	40.4	*β* 1.60, d (13.2)		1.33, 0.72
		α 0.72, m		1.60, 1.54
2	19.4	*β* 1.54, m		1.33, 0.76, 0.72
		α 1.33, m[c]		1.73, 1.60, 1.54, 0.76
3	41.7	*β* 1.73, d (13.2)		1.33, 0.76
		α 0.76, m[c]		1.73, 1.54, 1.33
4	38.1			
5	57.2	0.76, m[c]		1.47, 1.33
6	19.0	*β* 1.47, d (13.8)		1.33, 0.76
		α 1.33, m[c]		1.47, 1.16, 0.76
7	42.8	*β* 1.36, m		1.16
		α 1.16, dd (13.2, 9.6)		1.36, 1.33
8	38.0			
9	59.4	0.86, m		2.01, 1.79
10	34.0			
11	27.9	*α* 2.01, m		3.74, 0.86
		β 1.79, m		3.74, 0.86
12	77.0	3.74, dd (10.8, 3.6)	149.7, 16.9	2.01, 1.79
13	43.9			
14	53.1	1.17, d (12.6)		2.01
15	28.1	*α* 2.37, dd (12.6, 7.2)		5.13, 2.01
		β 2.01, m		5.13, 2.37, 1.17
16	69.4	5.13, dd (8.7, 7.5)	149.7, 133.0, 28.1, 16.9[e]	2.37, 2.01
17	133.0			
18	149.7	7.94, s	172.0, 133.0, 69.4, 53.1, 43.9	
19	172.0			
20	33.9	0.88, s	57.2, 41.7, 38.1, 22.0	
21	22.0	0.81, s[c]	57.2, 38.1, 33.9	
22	16.9[c]	0.81, s[c]	57.2, 40.4, 34.0	
23	18.2	0.91, s	59.4, 53.1, 38.0	
24	16.9[c]	1.40, s	149.7, 77.0, 53.1, 43.9	

[c]Overlapped with other signals.
[e]Long correlation

Table S.9. HRMS analysis results of known compounds (**9**–**18**)

Compounds	Formula	Calculated	Observed $[M+H]^+$	Observed $[M+Na]^+$
**9**	$C_{25}H_{38}O_3$	387.2894	387.2874	409.2691
**10**	$C_{29}H_{44}O_6$	511.3030		511.3016
**11**	$C_{25}H_{38}O_3$	387.2894	387.2881	409.2693
**12**	$C_{28}H_{42}O_6$	497.2874		497.2853
**13**	$C_{25}H_{38}O_4$	403.2843	403.2842	425.2652
**14**	$C_{25}H_{38}O_5$	441.2611		441.2604
**15**	$C_{25}H_{38}O_5$	419.2792	419.2789	441.2600
**16**	$C_{25}H_{38}O_4$	403.2843	403.2837	425.2651
**17**	$C_{27}H_{40}O_6$	461.2898	461.2909	483.2706
**18**	$C_{29}H_{42}O_6$	509.2874		509.2856

Table S.10. Inhibitory activity of the isolated compounds (**1−18**) against cancer cell growth

Compounds	Cancer Cell Lines			Compounds	Cancer Cell Lines	
	HeLa	MCF-7			HeLa	MCF-7
**1**	53.4 ± 1.11	27.3 ± 1.09		**11**	> 80.0	49.4 ± 1.25
**2**	46.2 ± 1.16	26.2 ± 1.12		**12**	41.9 ± 1.19	24.3 ± 1.09
**3**	60.4 ± 1.15	29.9 ± 1.08		**13**	65.2 ± 1.13	40.8 ± 1.14
**4**	61.3 ± 1.12	45.9 ± 1.25		**14**	58.4 ± 1.14	30.9 ± 1.11
**5**	70.7 ± 1.10	76.4 ± 1.24		**15**	> 80.0	51.3 ± 1.10
**6**	59.3 ± 1.16	33.8 ± 1.09		**16**	46.3 ± 1.18	20.0 ± 1.08
**7**	> 80.0	> 80.0		**17**	46.9 ± 1.14	36.5 ± 1.22
**8**	> 80.0	> 80.0		**18**	> 80.0	> 80.0
**9**	> 80.0	43.8 ± 1.09				
**10**	45.7 ± 1.10	27.7 ± 1.15		staurosporine	0.18 ± 0.07	0.13 ± 0.12

Table S.11. The cytotoxic effect of isolated compounds (**1**–**18**) on Hela and MCF-7 cancer cell lines at the various concentrations.

Compounds	Cancer Cell Lines	
	**Hela**	**MCF-7**
**1**	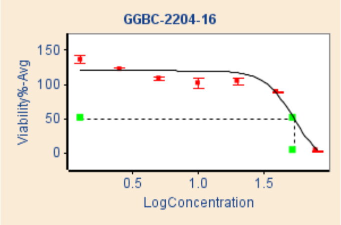	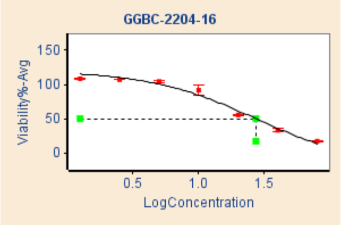
**2**	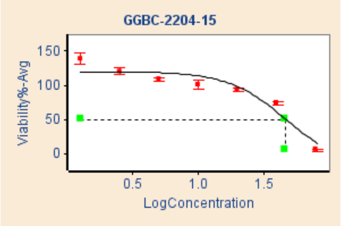	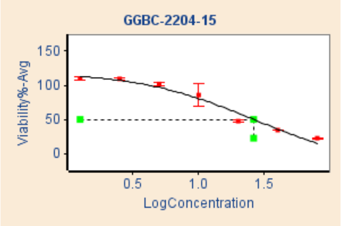
**3**	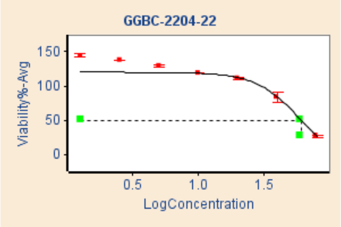	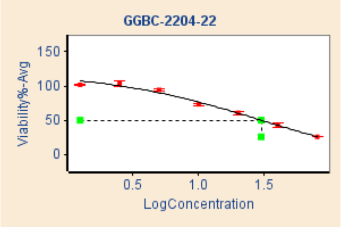
**4**	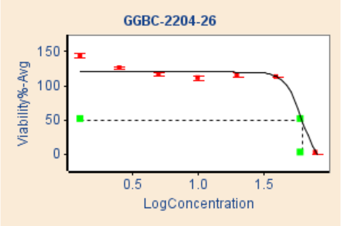	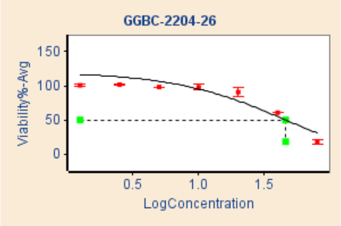
**5**	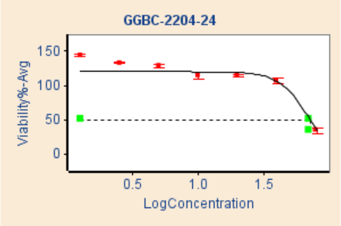	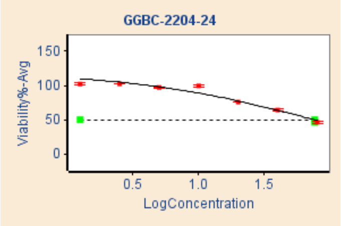
**6**	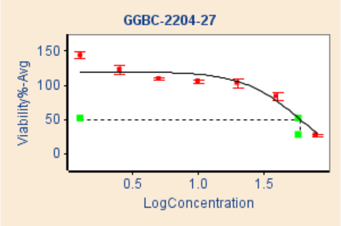	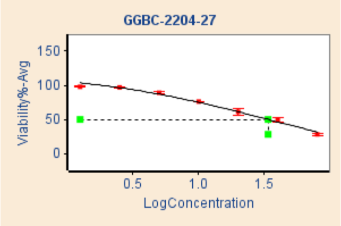

7

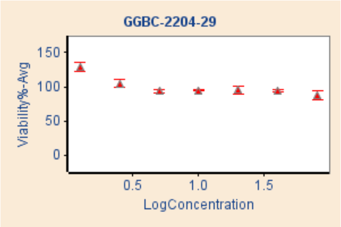

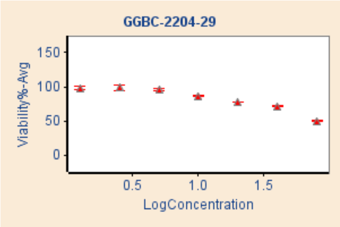

8

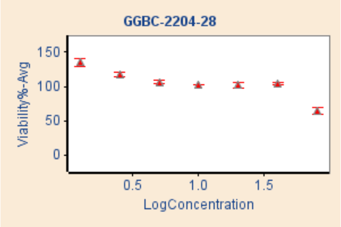

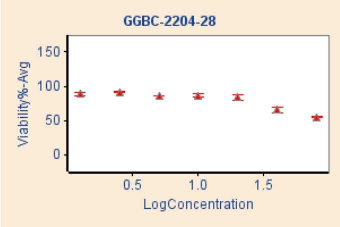

9

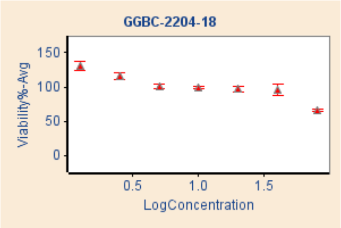

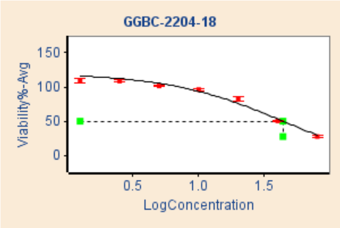

10

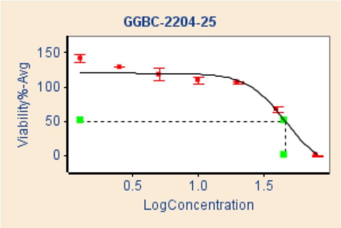

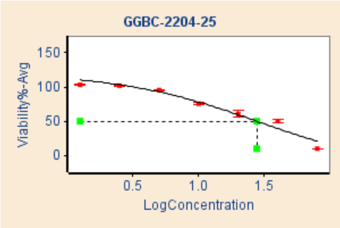

11

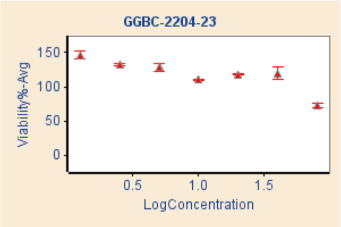

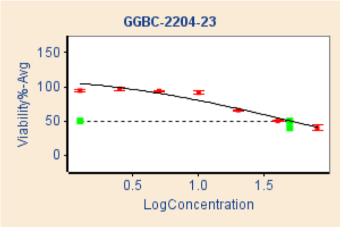

12

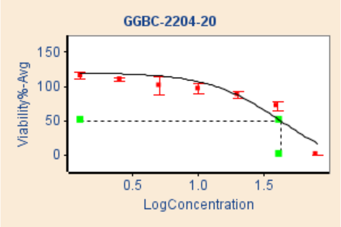

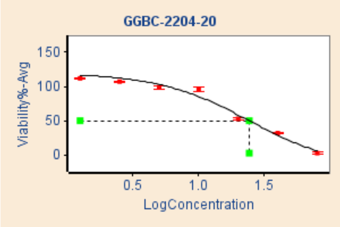

13

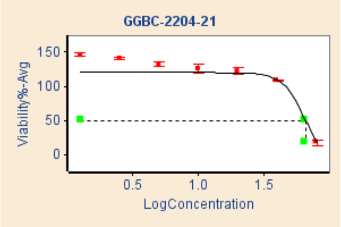

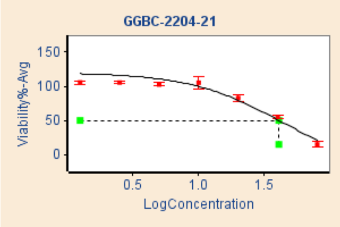

14

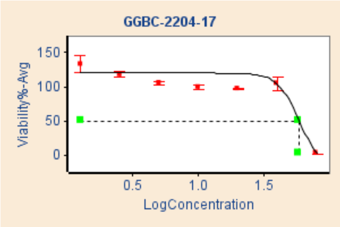

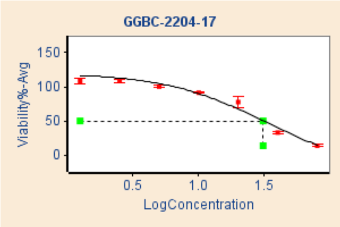

15

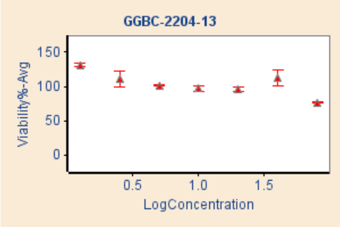

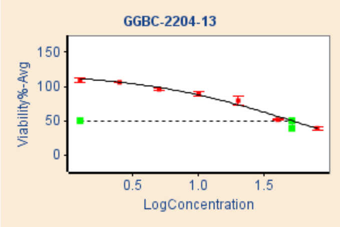

16

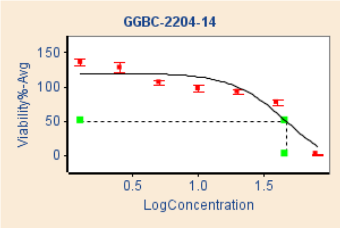

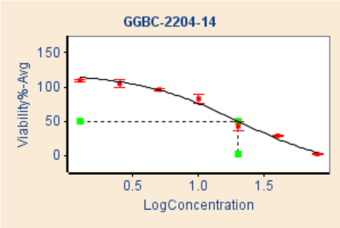

17

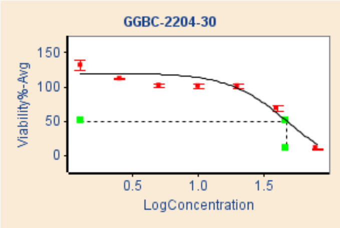

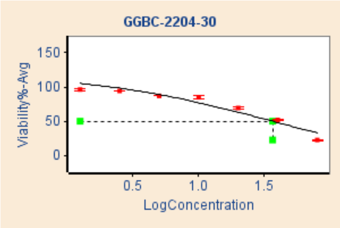

18

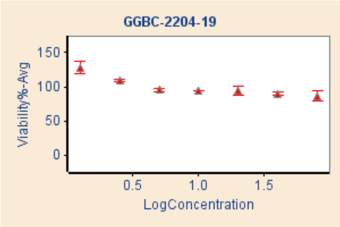

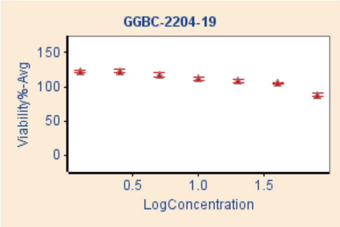

starosporine

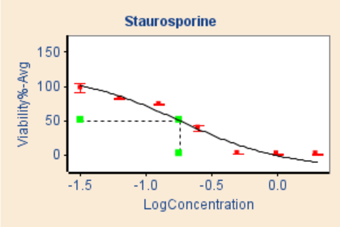

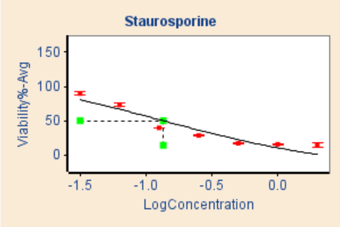

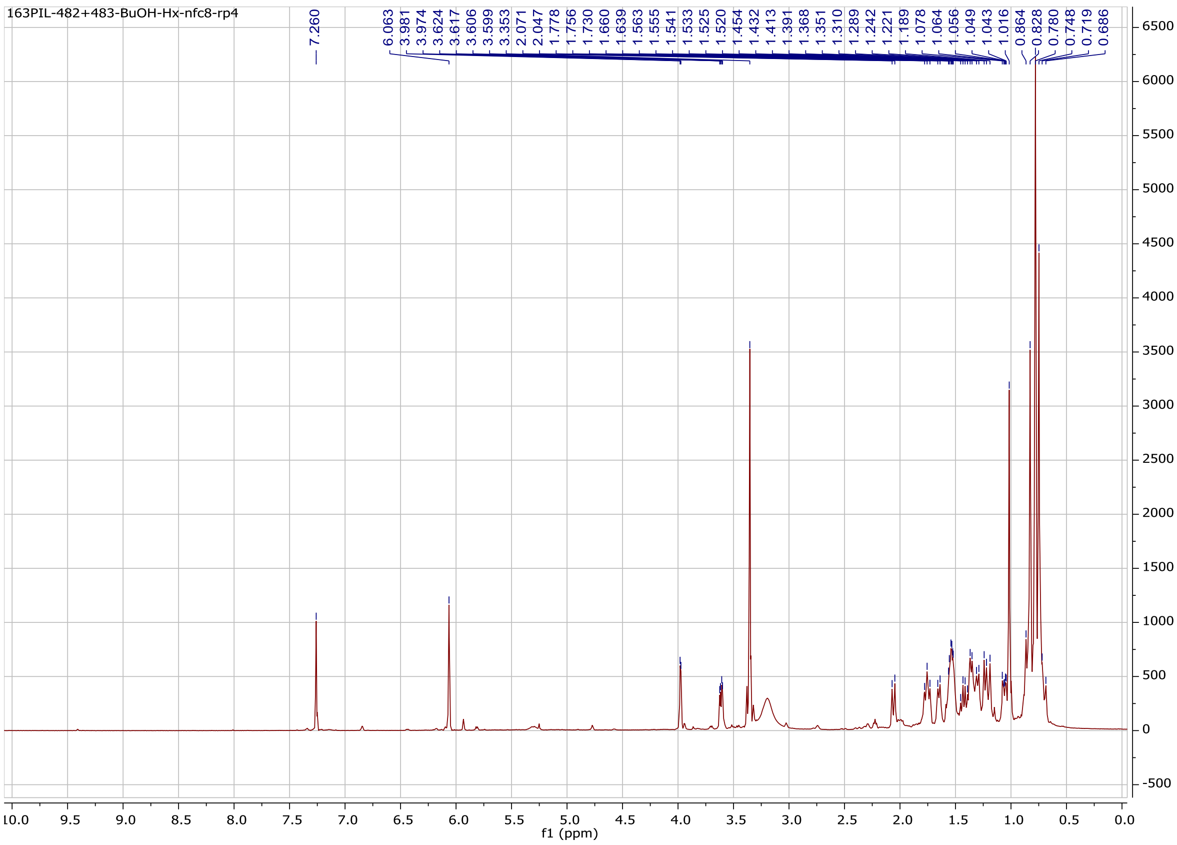

Fig. S.1.1. ^{1}H NMR spectrum of compound **1** in $CDCl_3$

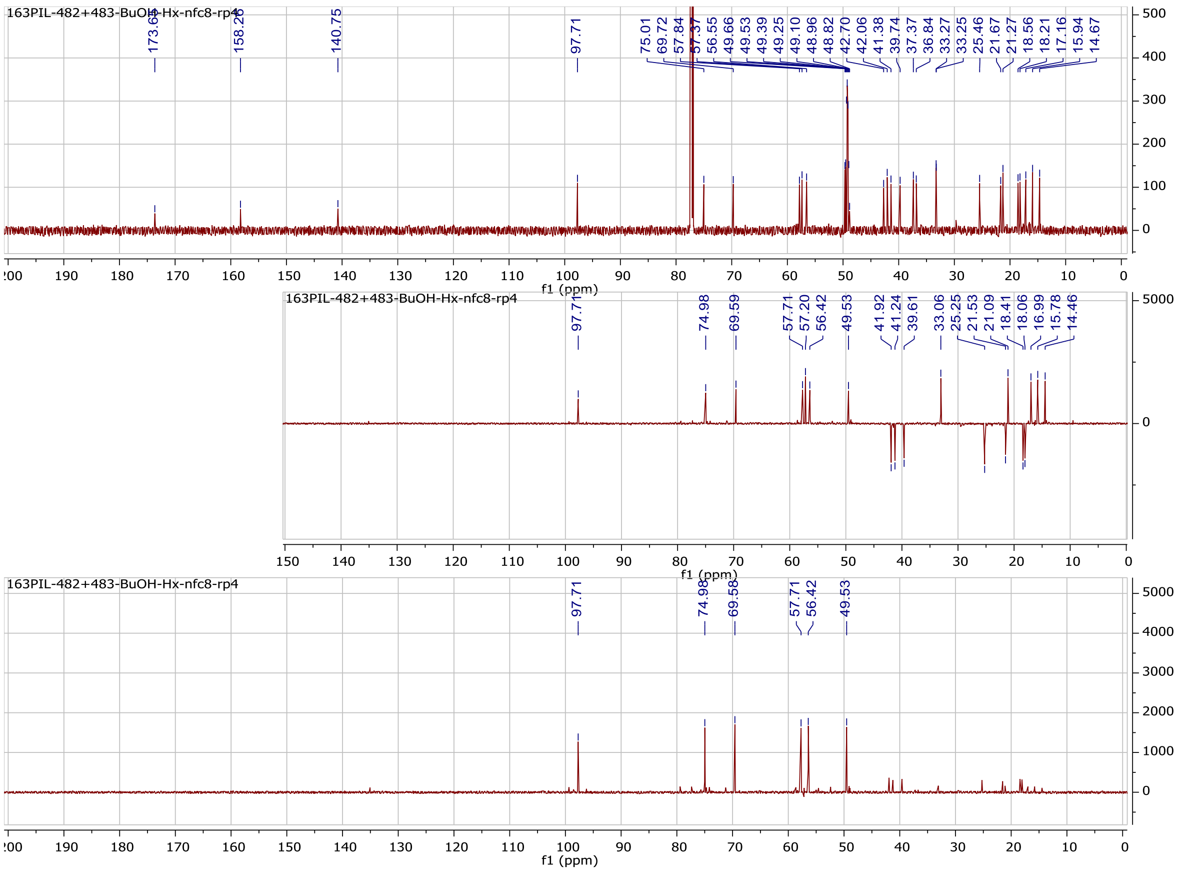

Fig. S.1.2. ^{13}C NMR and DEPT spectra of compound **1** in $CDCl_3$

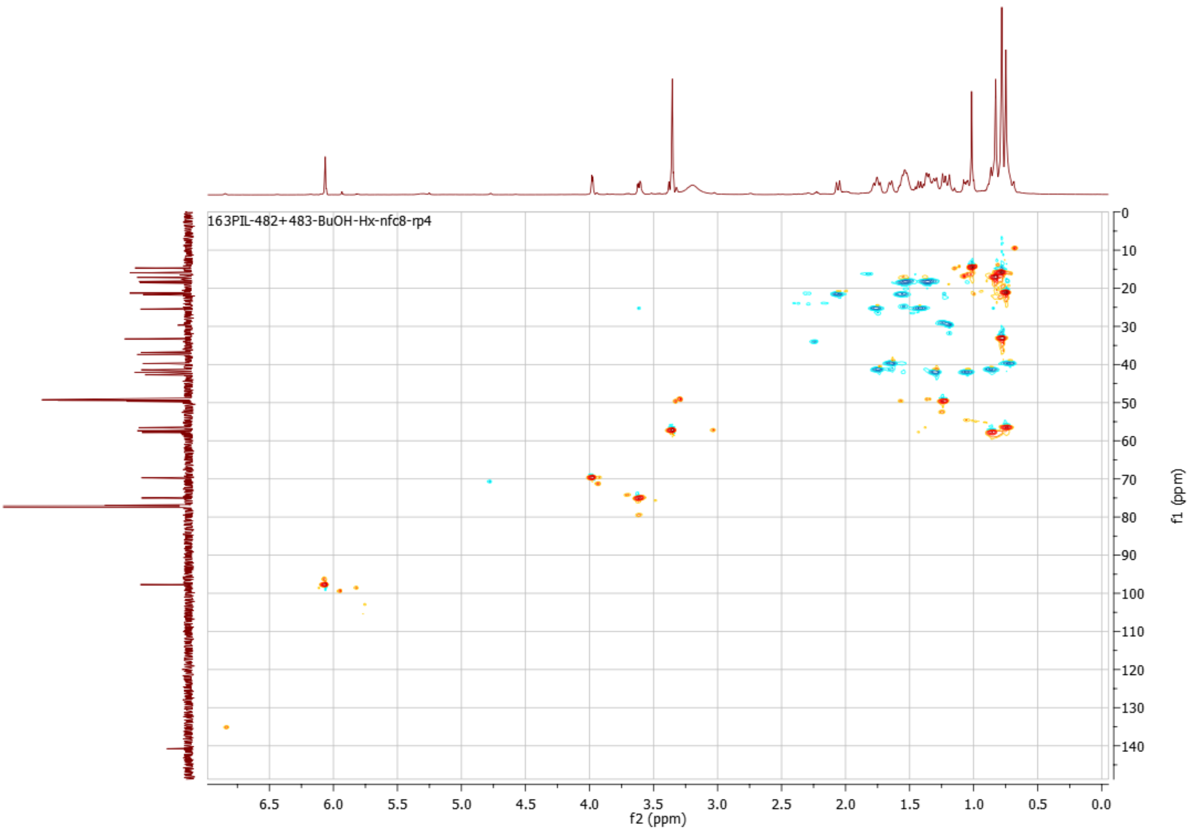

Fig. S.1.3. HSQC spectrum of compound **1** in $CDCl_3$

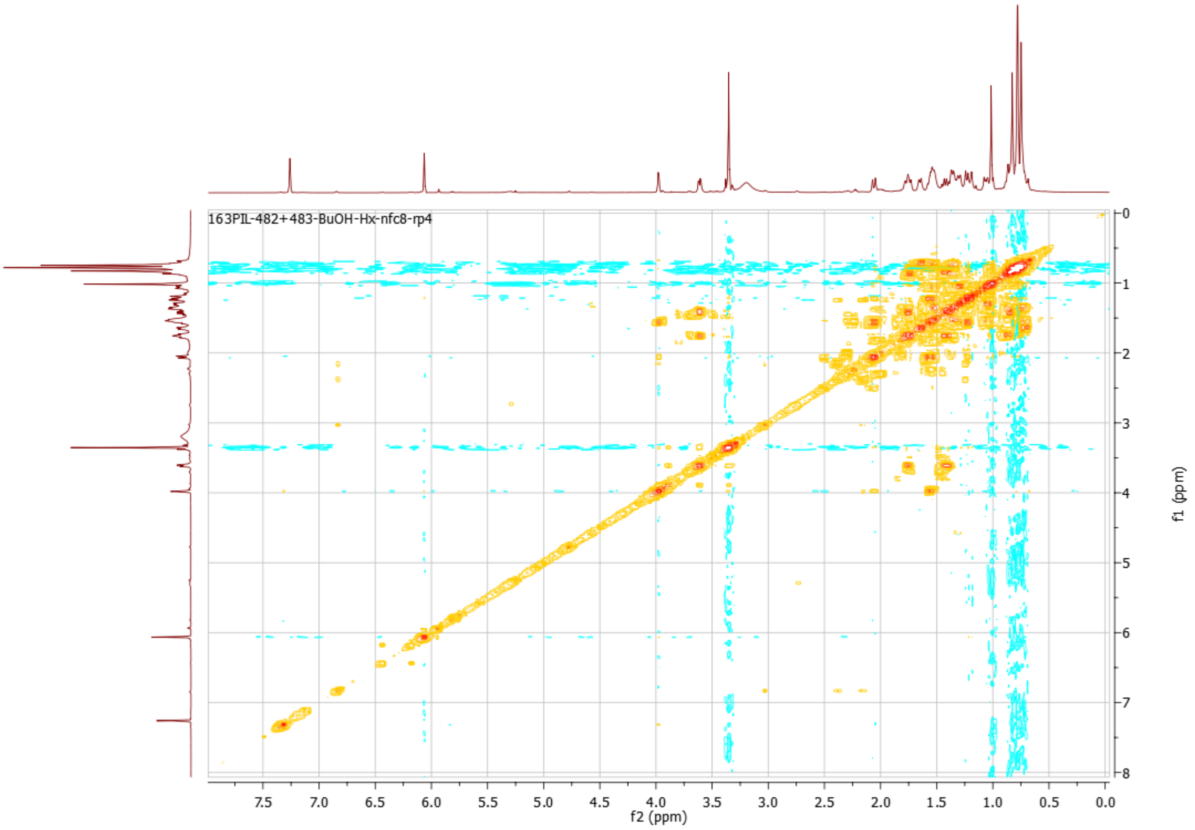

Fig. S.1.4. COSY spectrum of compound **1** in $CDCl_3$

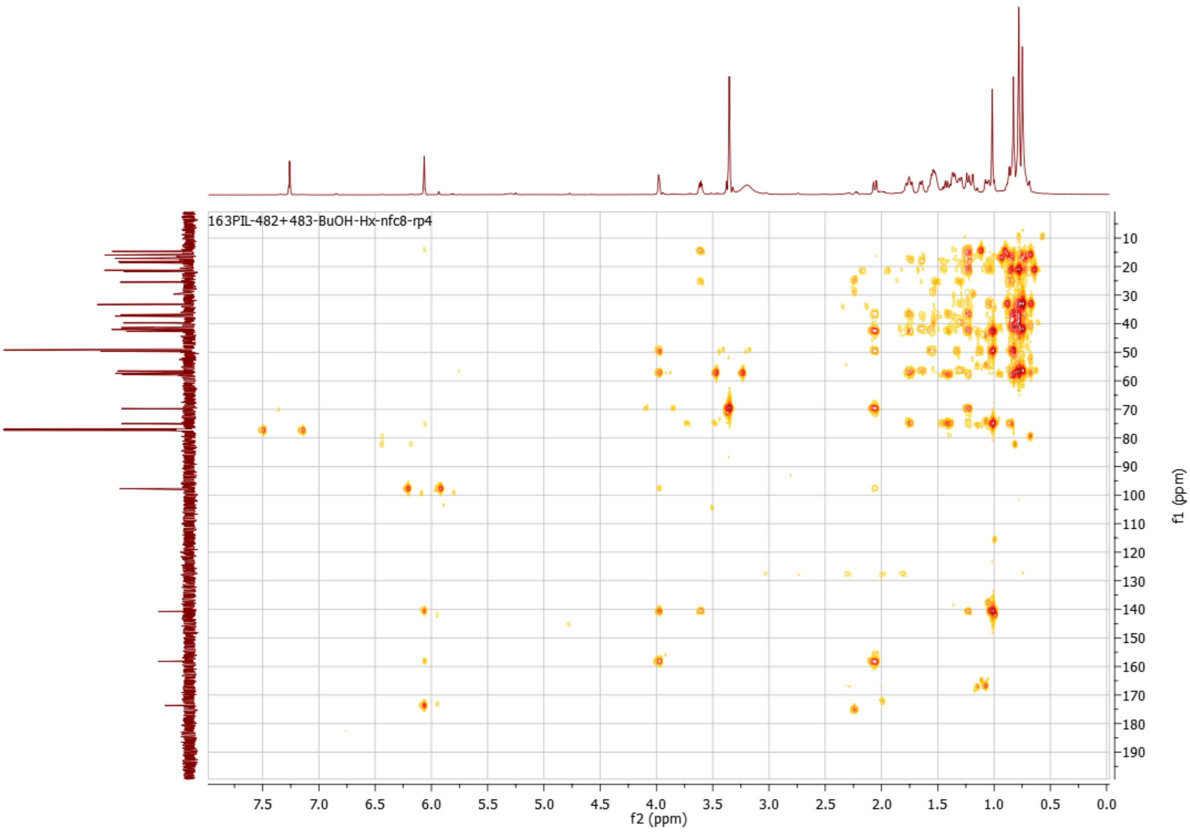

Fig. S.1.5. HMBC spectrum of compound **1** in $CDCl_3$

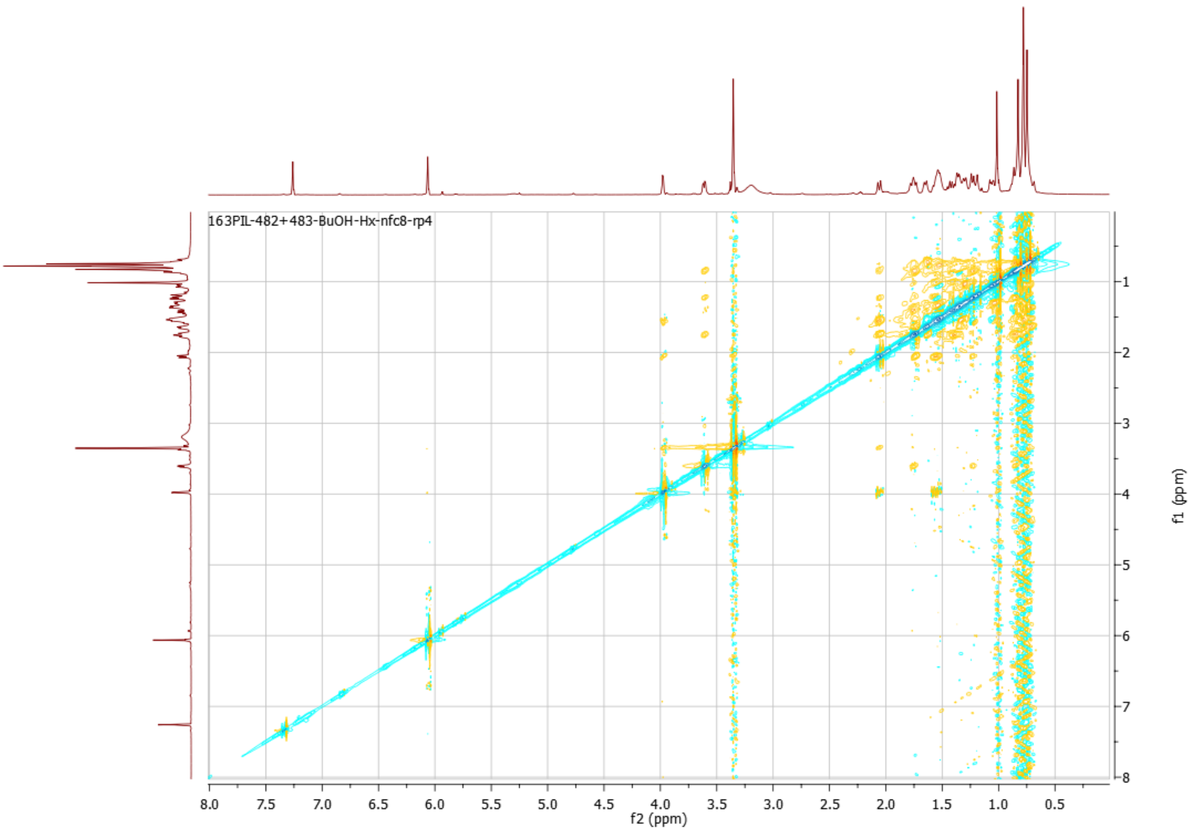

Fig. S.1.6. ROESY spectrum of compound **1** in $CDCl_3$

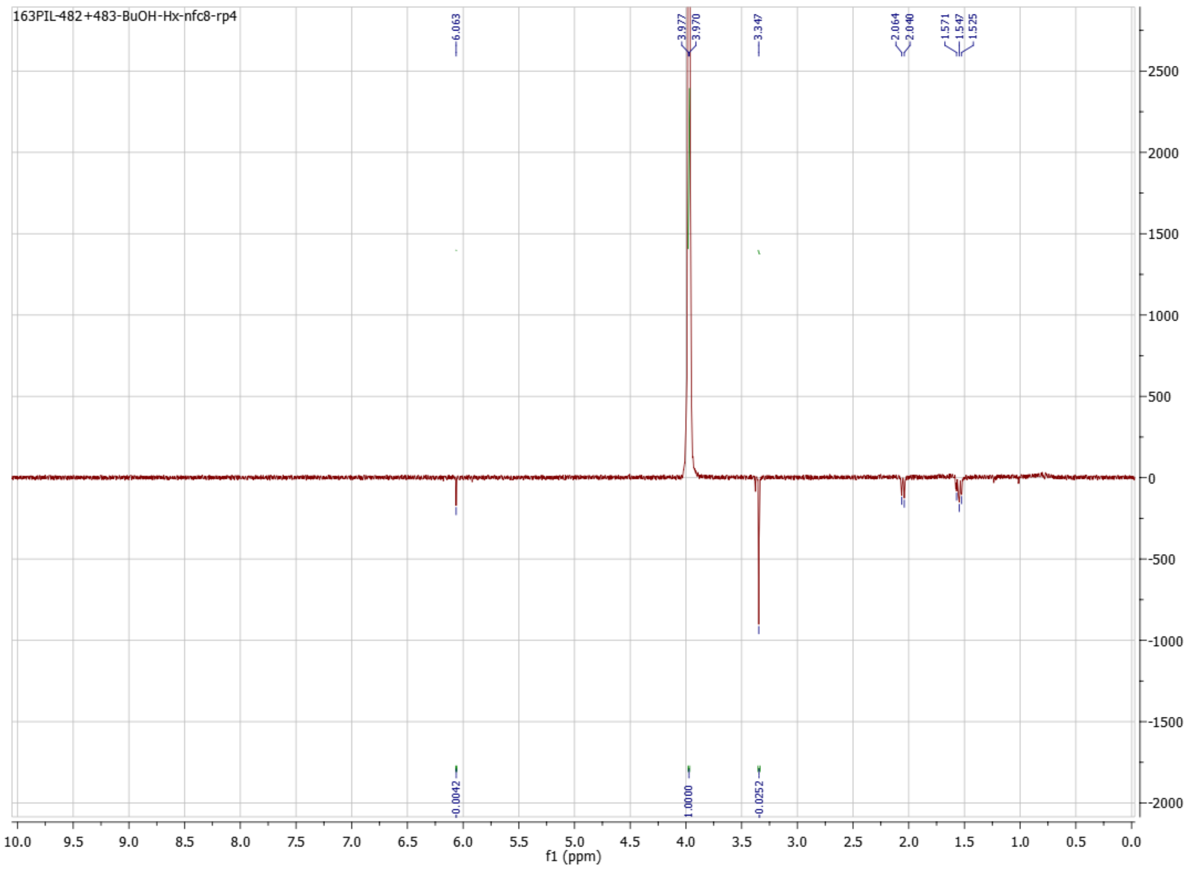

Fig. S.1.7. 1D NOESY spectrum of compound **1** in $CDCl_3$

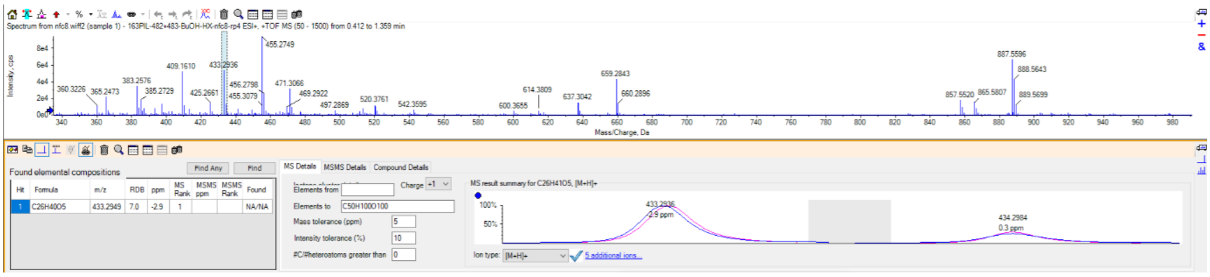

Fig. S.1.8. HR-ESIMS spectrum of compound **1**

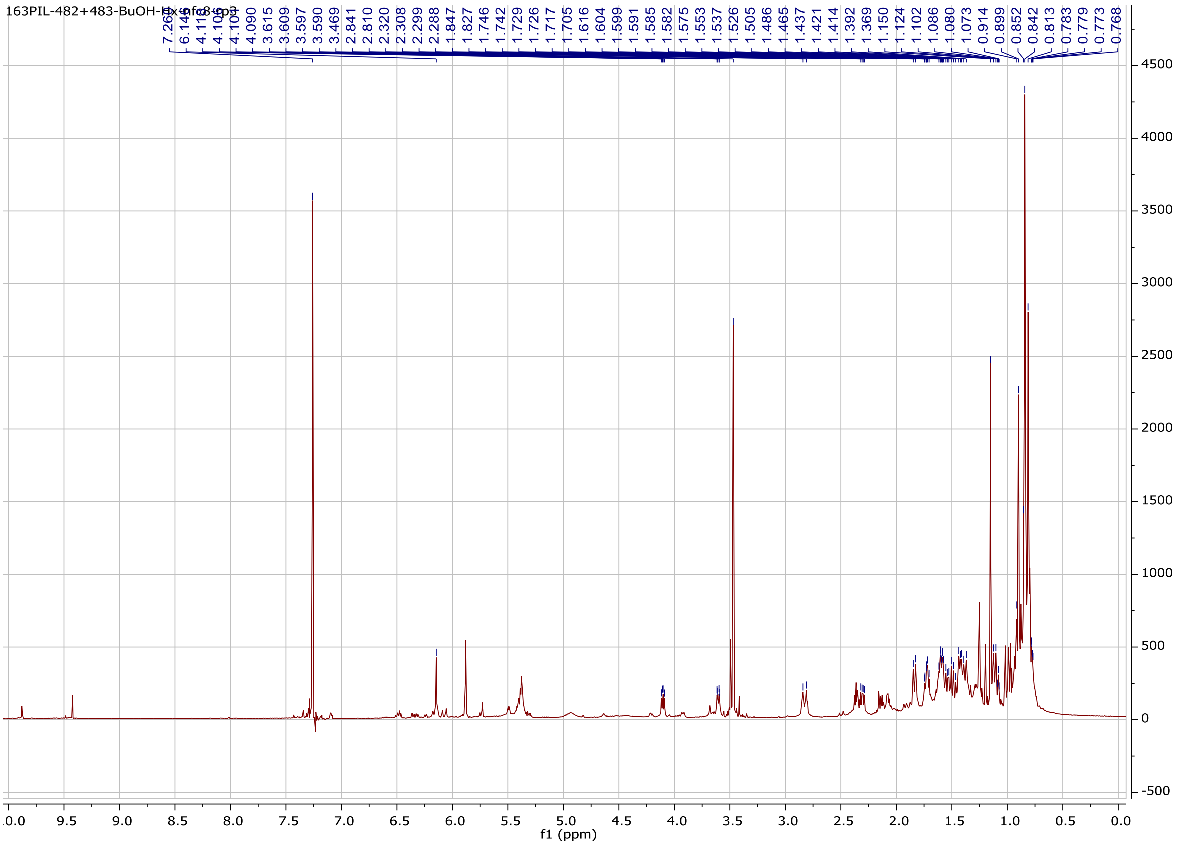

Fig. S.2.1. ^{1}H NMR spectrum of compound **2** in $CDCl_3$

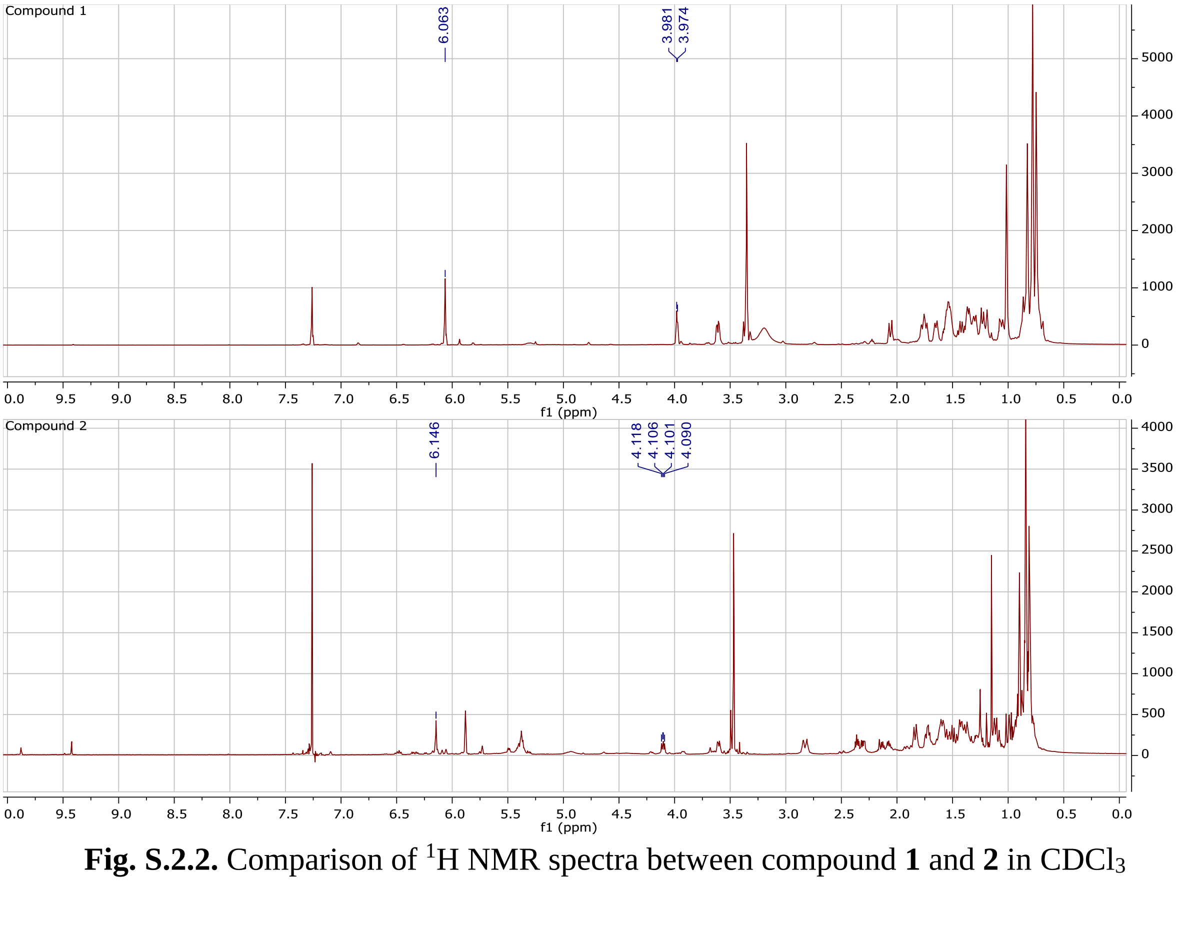

Fig. S.2.2. Comparison of ^{1}H NMR spectra between compound **1** and **2** in $CDCl_3$

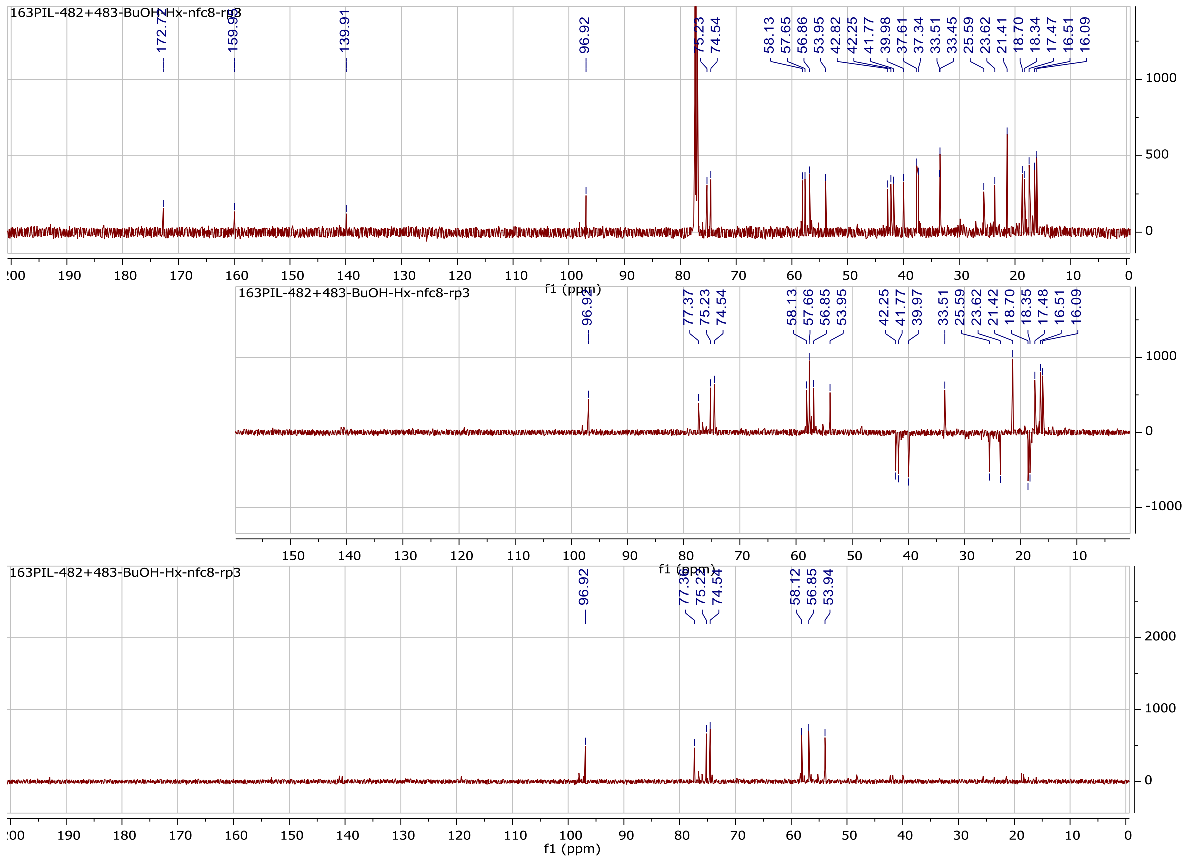

Fig. S.2.3. ^{13}C NMR and DEPT spectra of compound **2** in $CDCl_3$

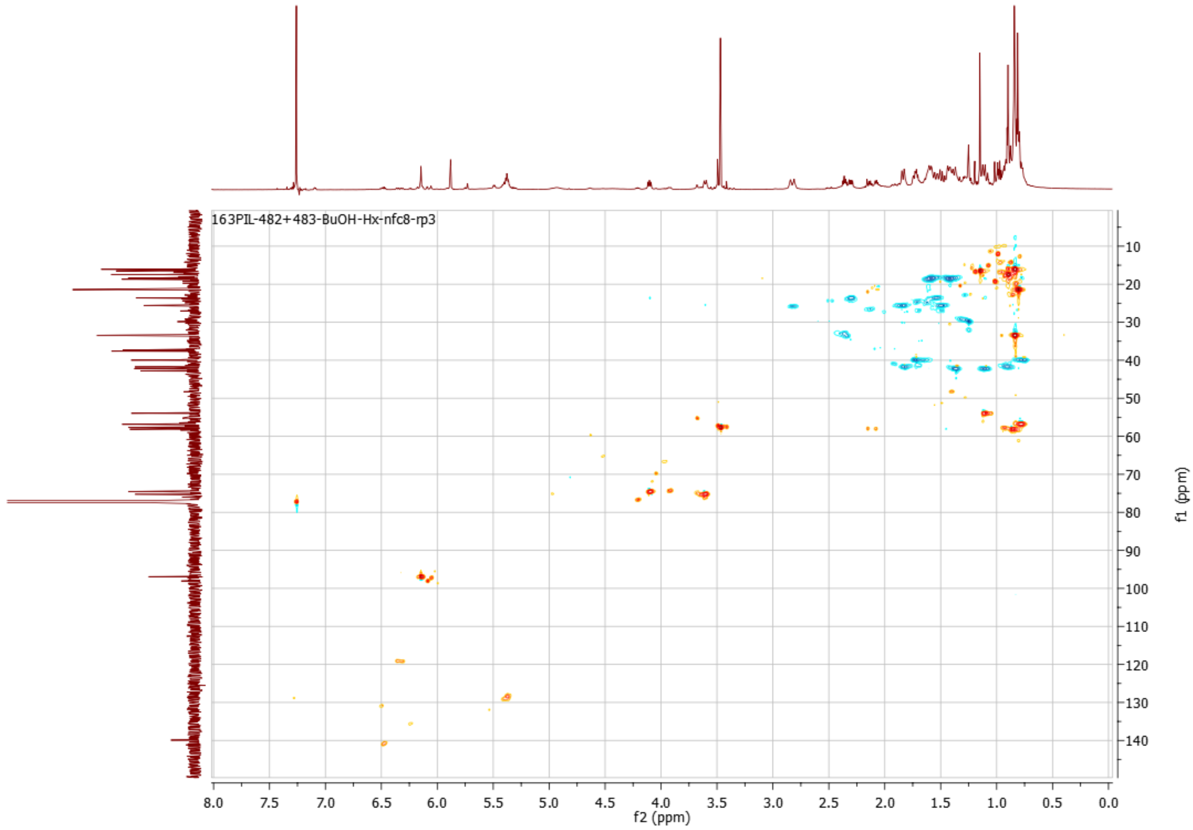

Fig. S.2.4. HSQC spectrum of compound **2** in $CDCl_3$

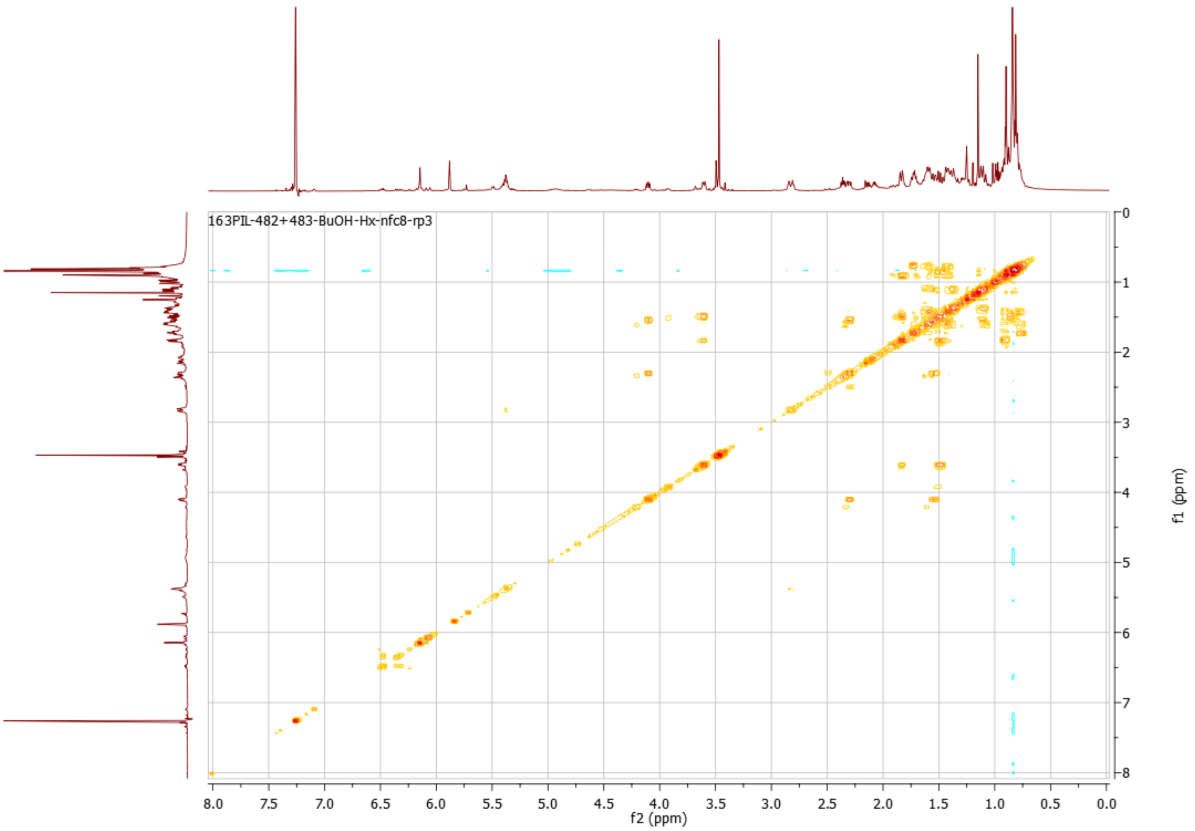

Fig. S.2.5. COSY spectrum of compound **2** in $CDCl_3$

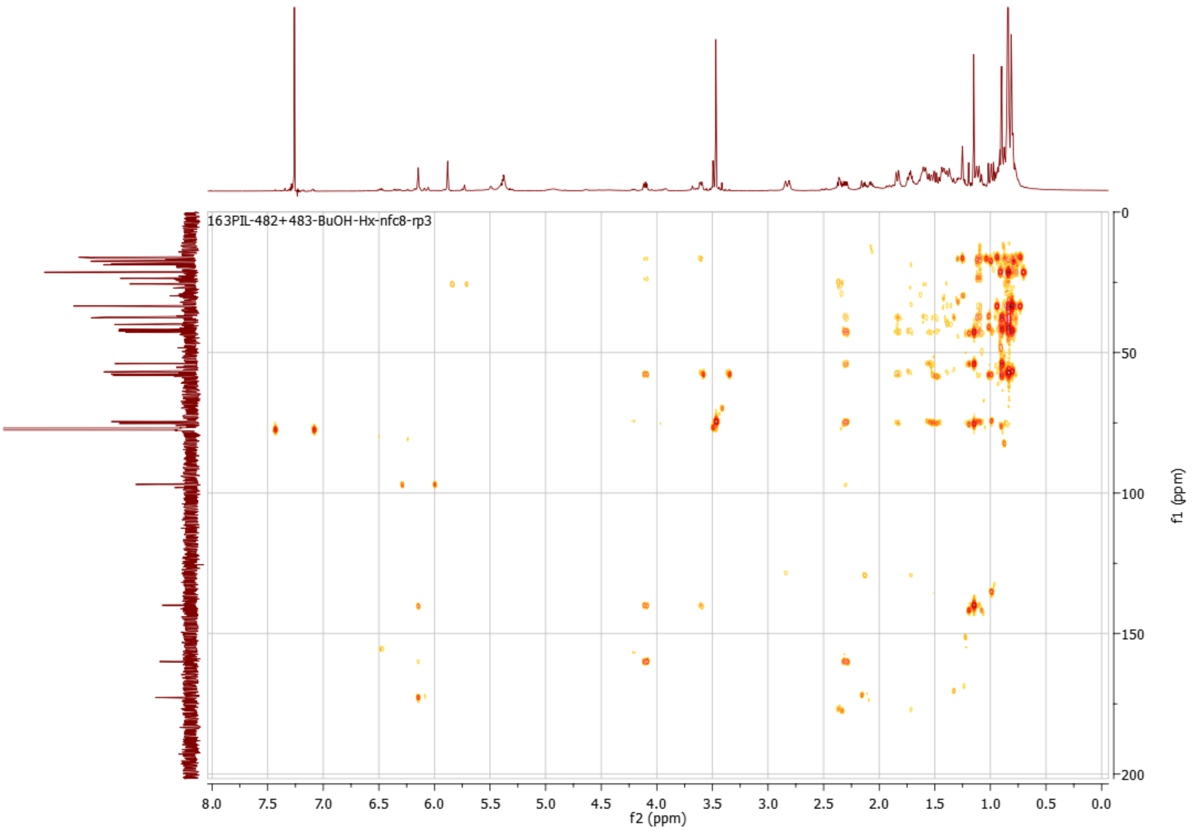

Fig. S.2.6. HMBC spectrum of compound **2** in $CDCl_3$

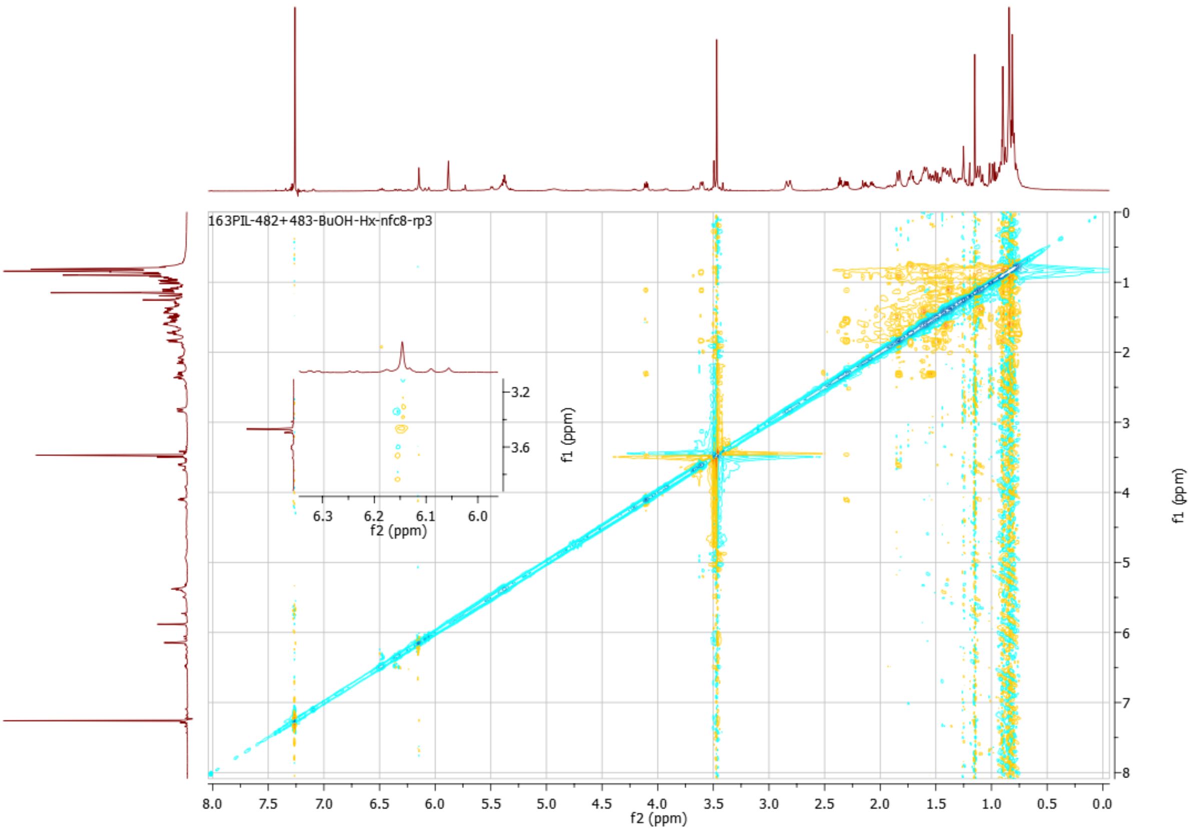

Fig. S.2.7. ROESY spectrum of compound **2** in $CDCl_3$

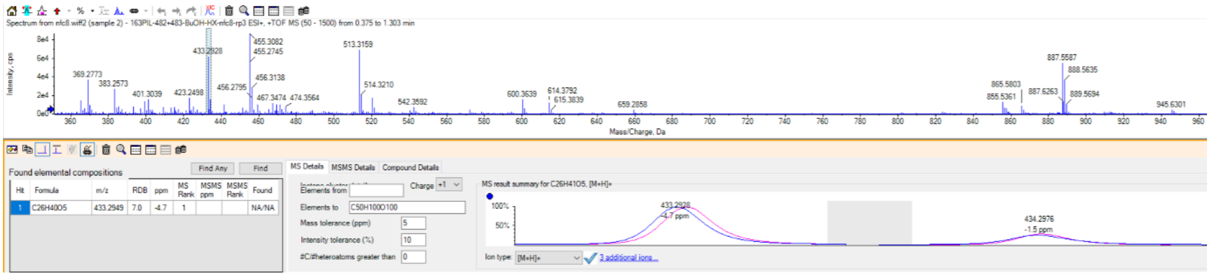

Fig. S.2.8. HR-ESIMS spectrum of compound **2**

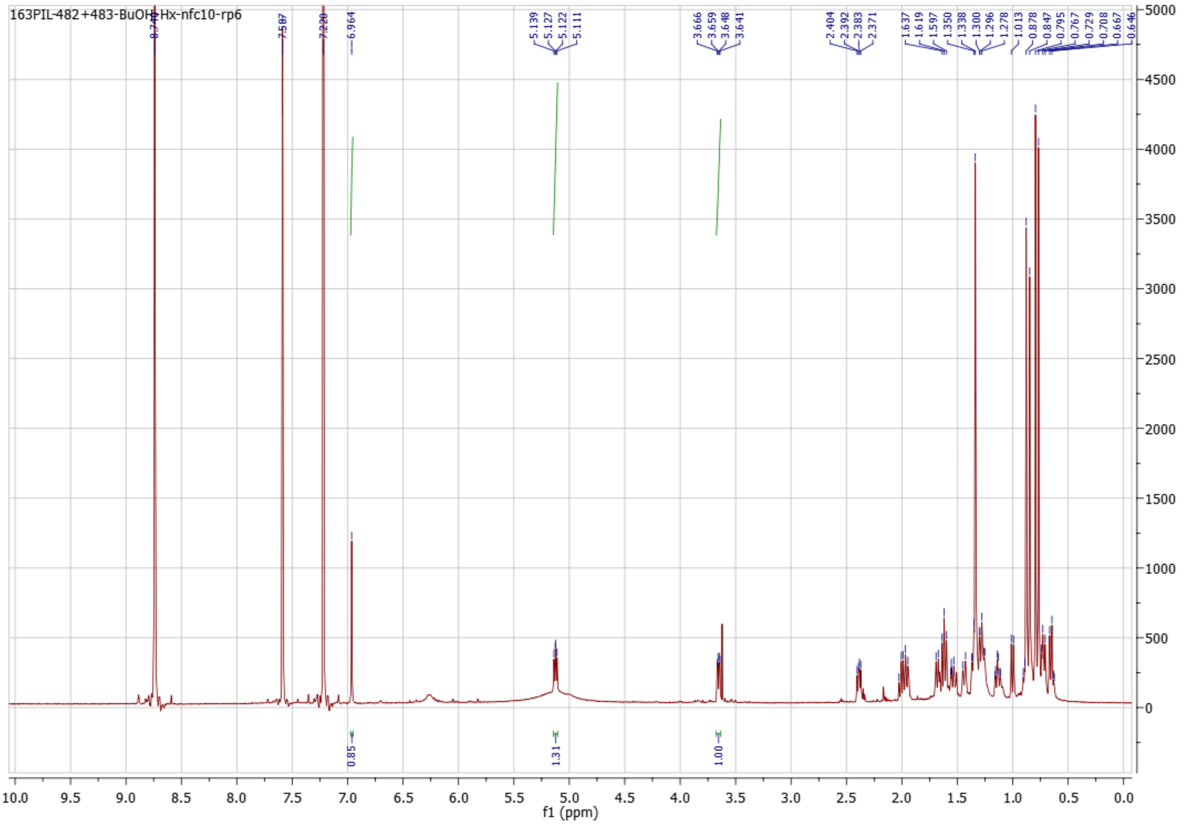

Fig. S.3.1. ^{1}H NMR spectrum of compound **3** in C_5D_5N

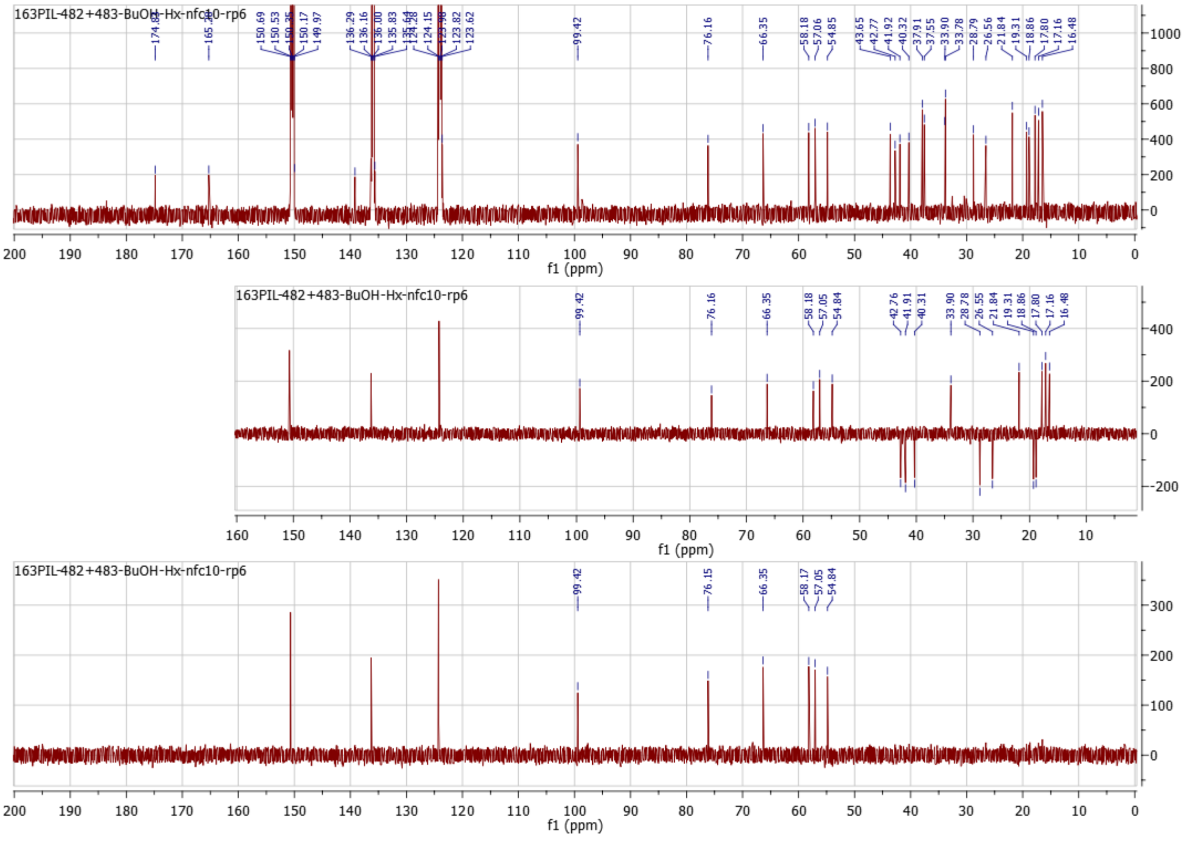

Fig. S.3.2. ^{13}C NMR and DEPT spectra of compound **3** in C_5D_5N

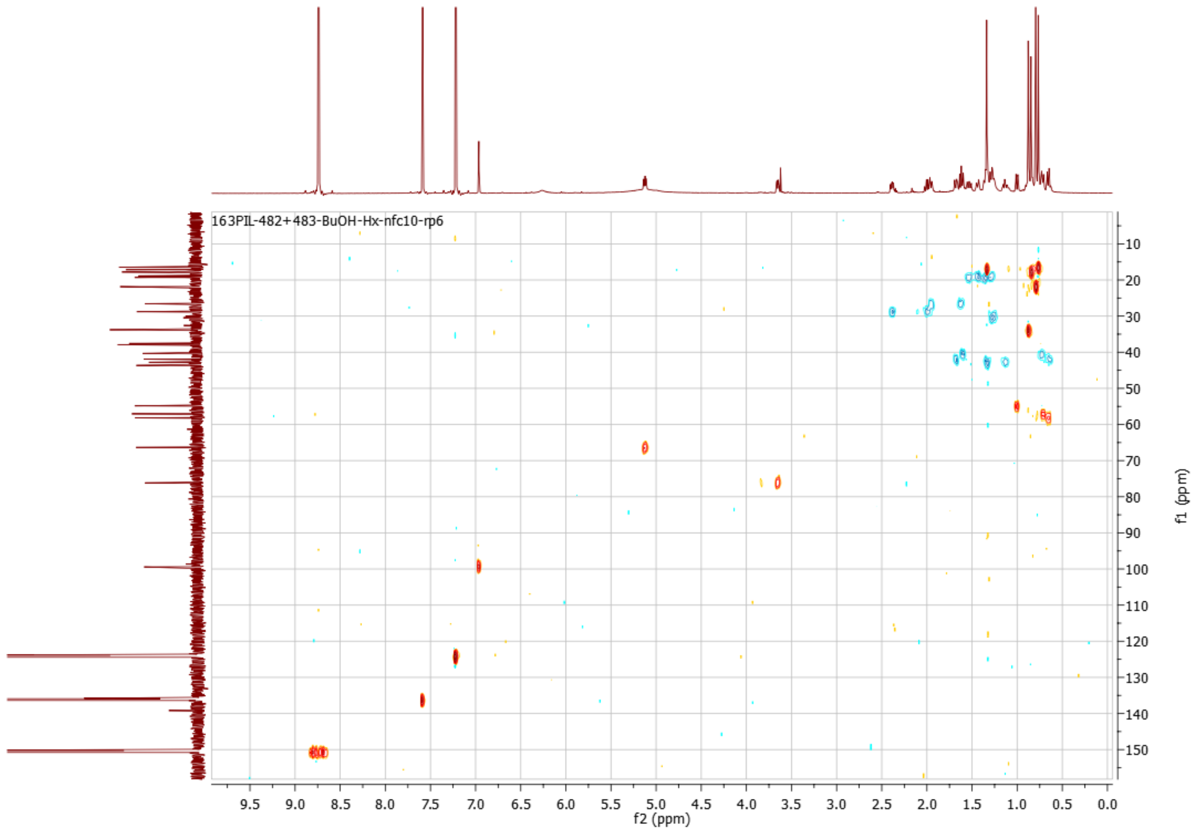

Fig. S.3.3. HSQC spectrum of compound **3** in C_5D_5N

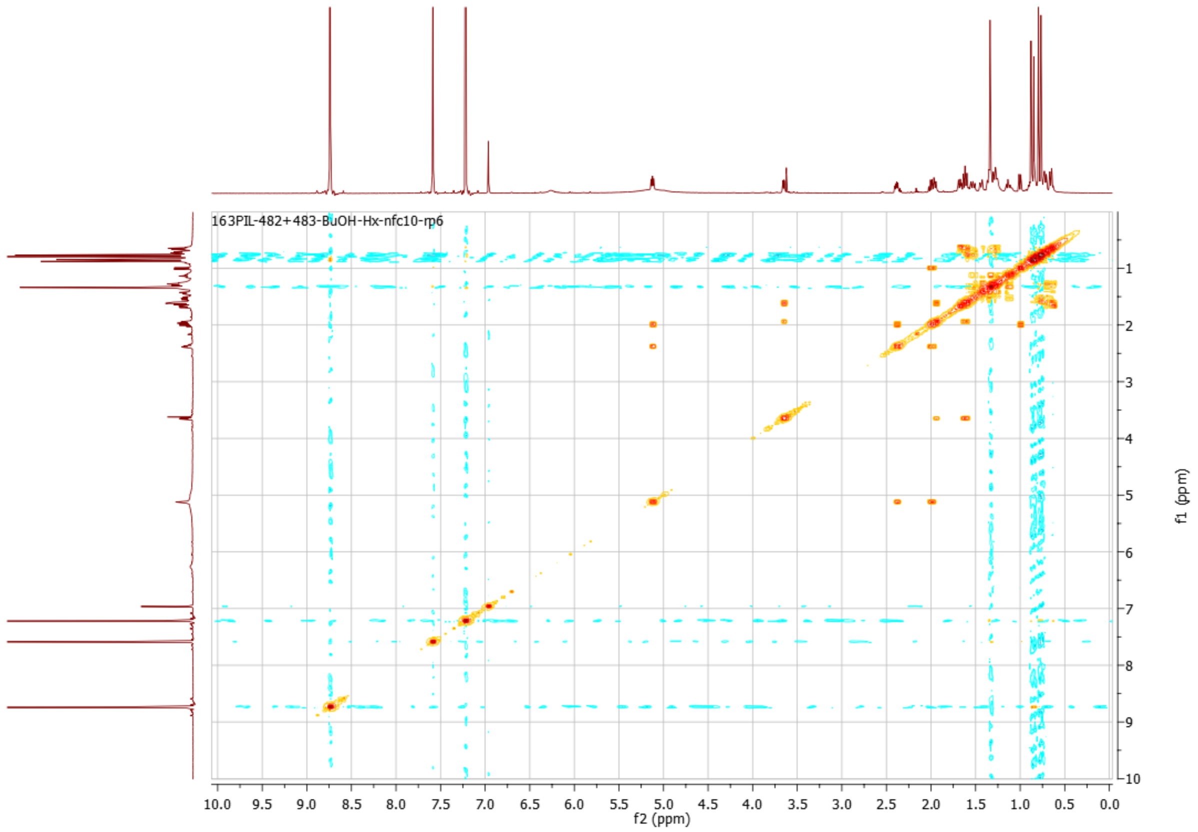

Fig. S.3.4. COSY spectrum of compound **3** in C_5D_5N

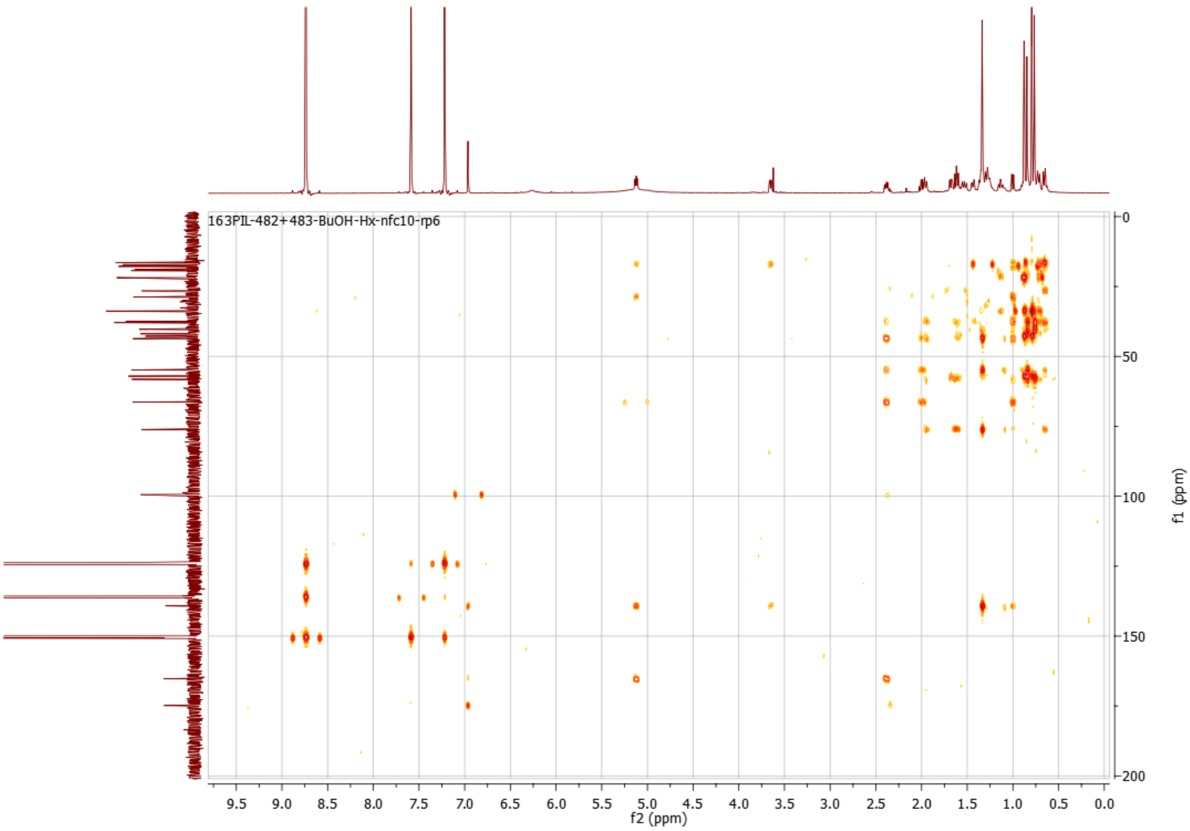

Fig. S.3.5. HMBC spectrum of compound **3** in C_5D_5N

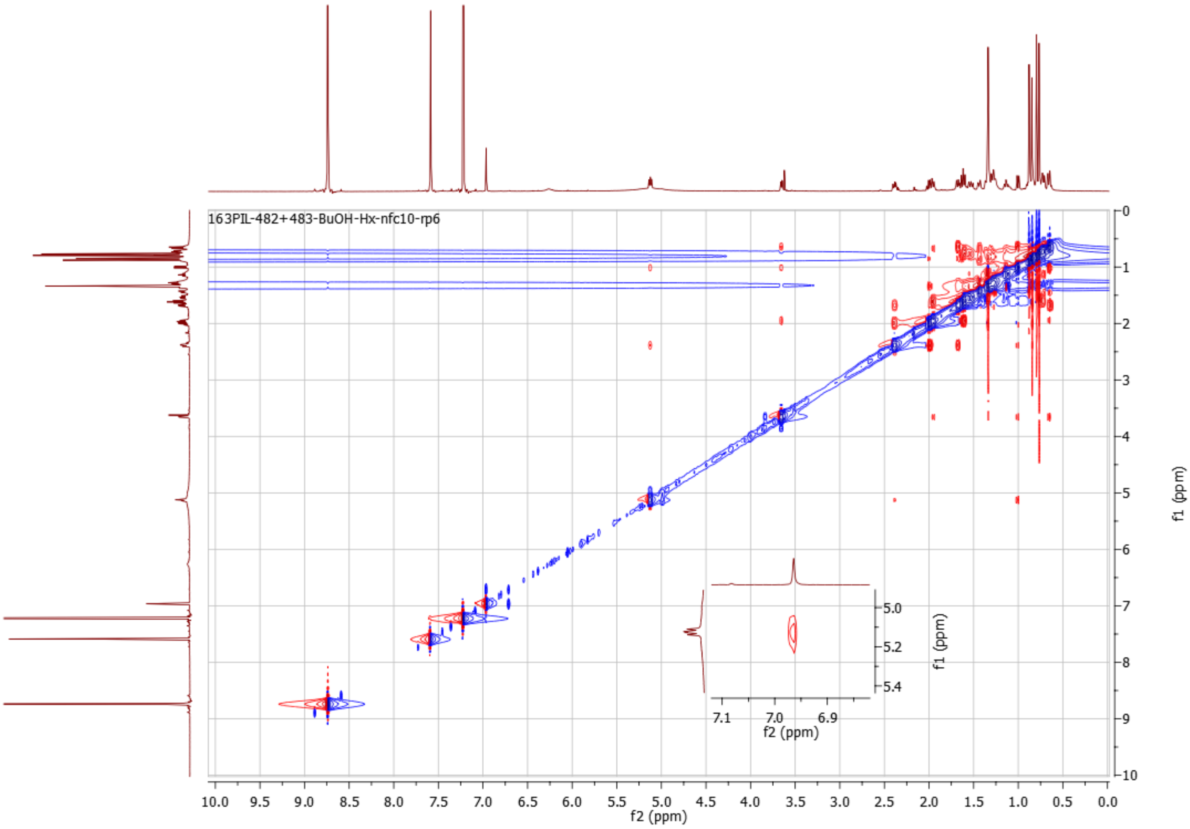

Fig. S.3.6. ROESY spectrum of compound **3** in C_5D_5N

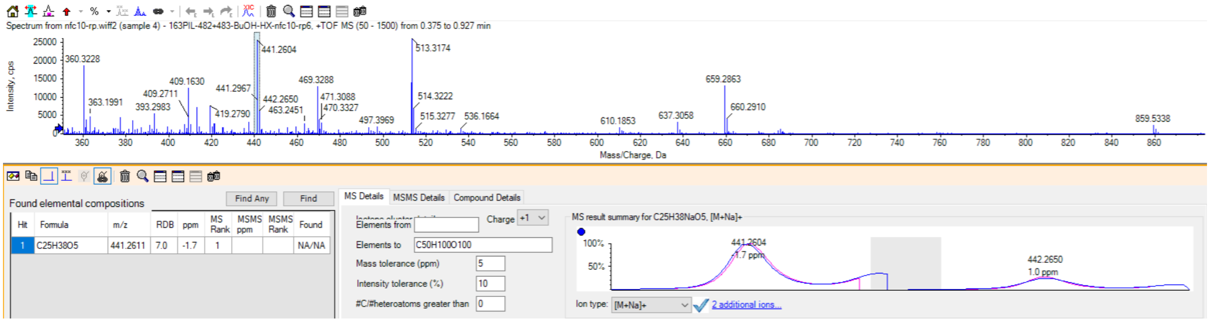

Fig. S.3.7. HR-ESIMS spectrum of compound **3**

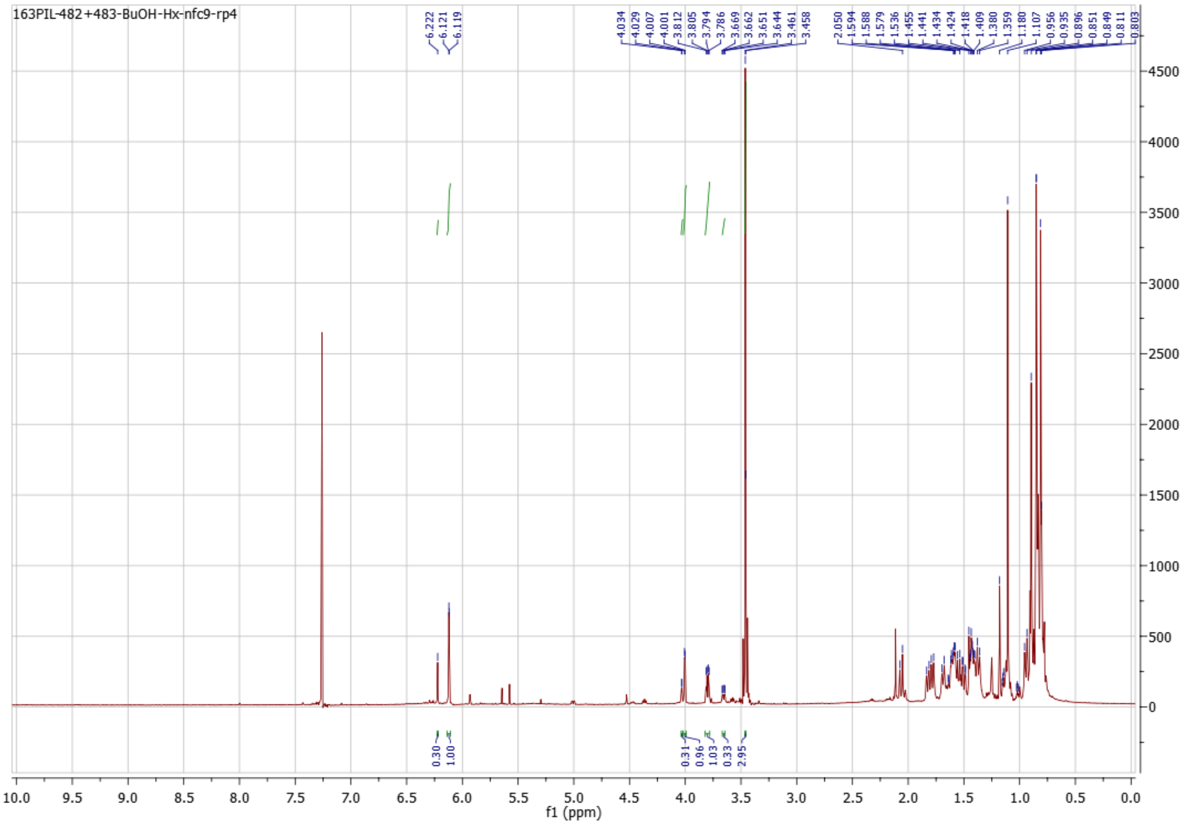

Fig. S.4.1. ^{1}H NMR spectrum of compound **4** in $CDCl_3$

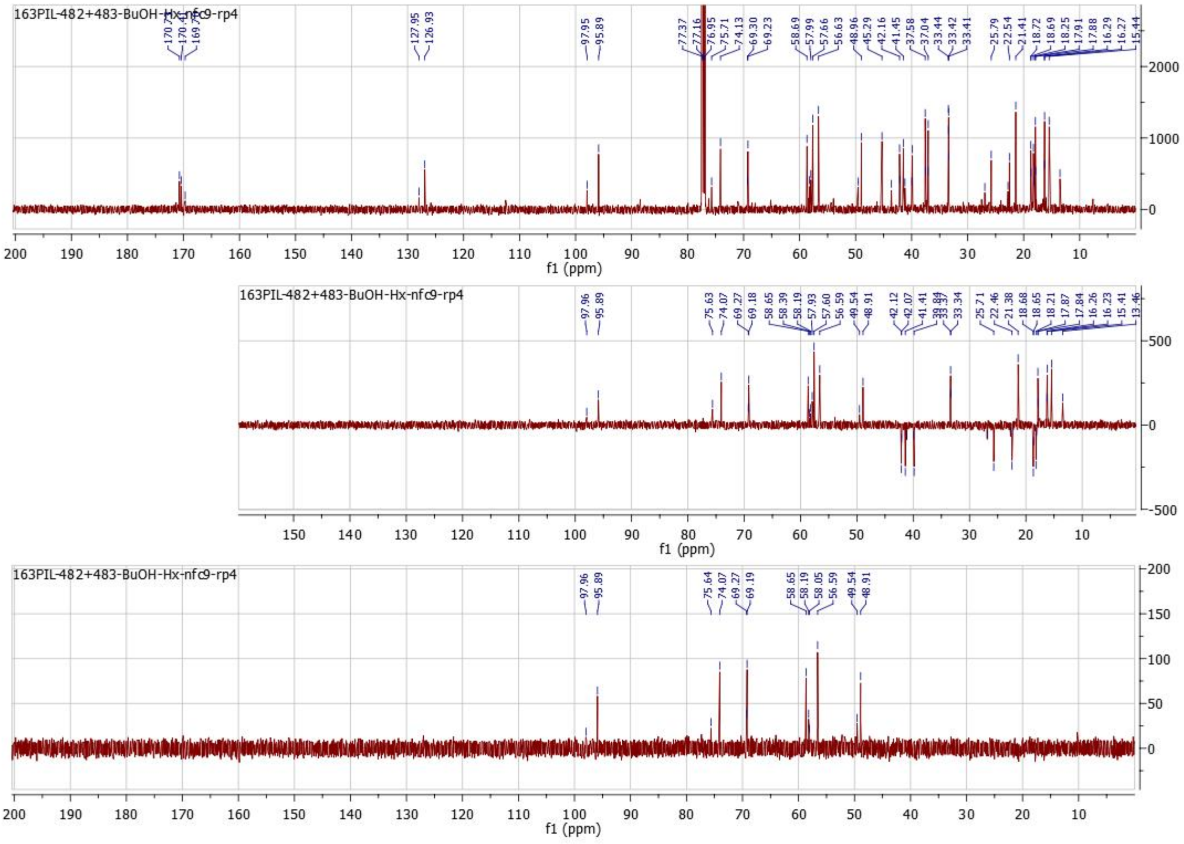

Fig. S.4.2. ^{13}C NMR and DEPT spectra of compound **4** in $CDCl_3$

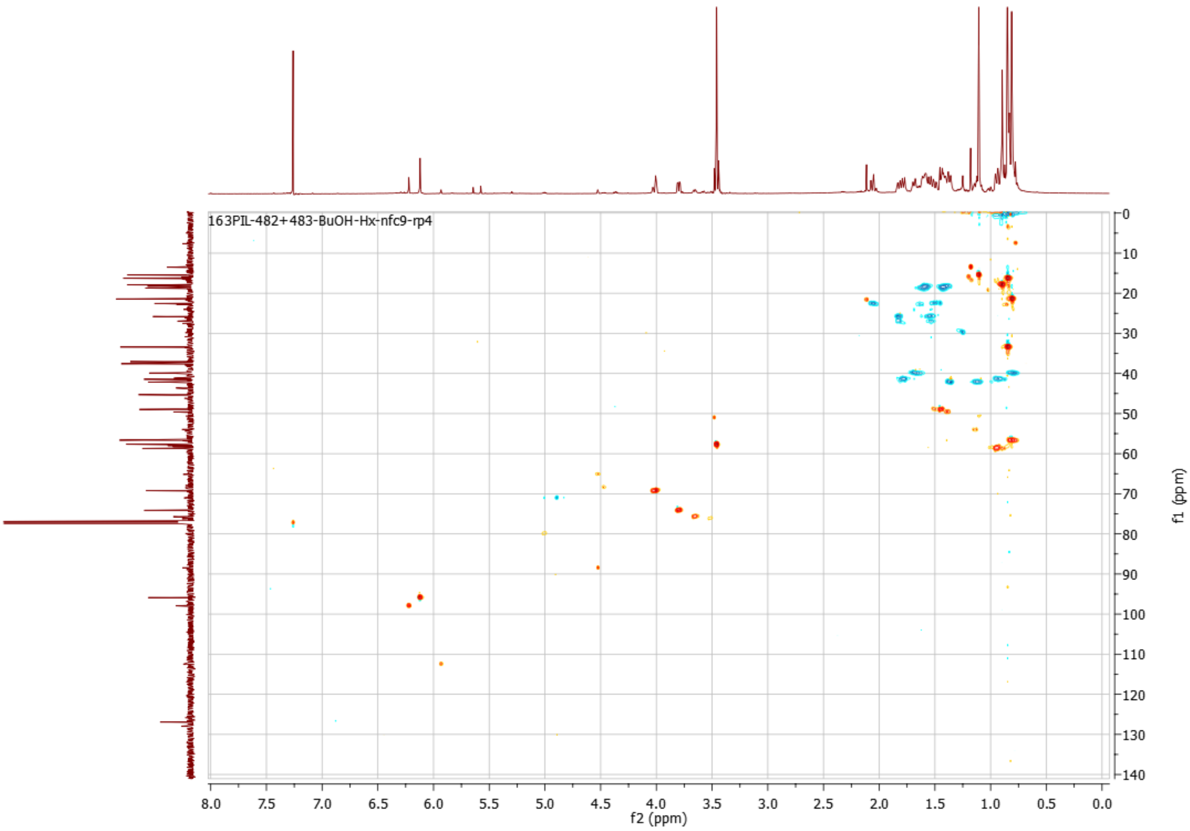

Fig. S.4.3. HSQC spectrum of compound **4** in $CDCl_3$

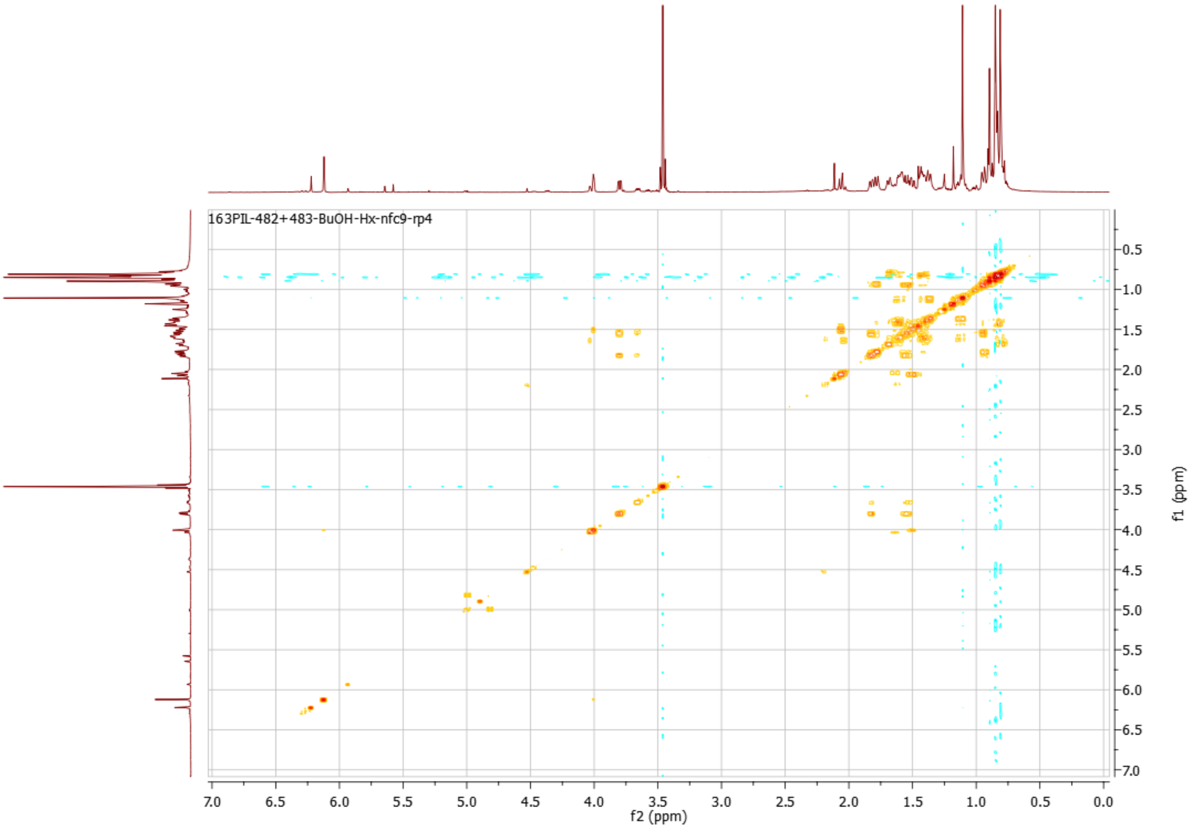

Fig. S.4.4. COSY spectrum of compound **4** in $CDCl_3$

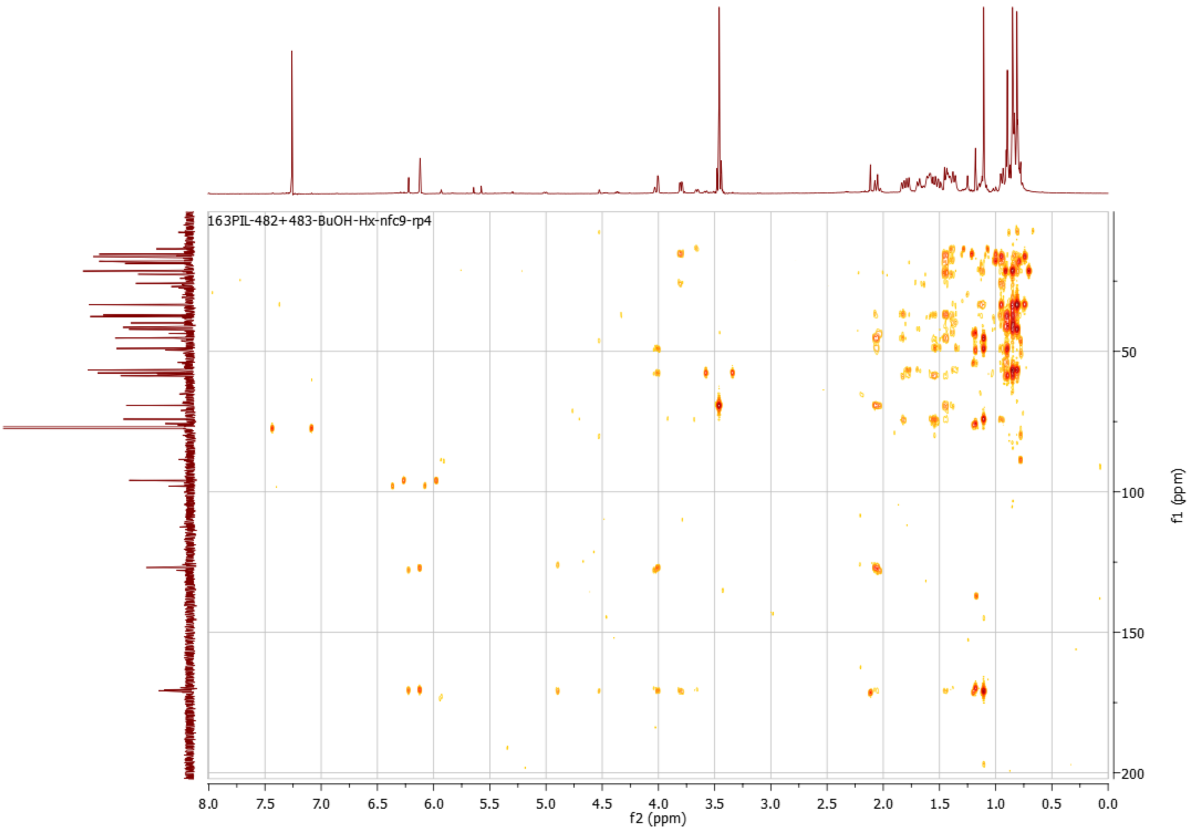

Fig. S.4.5. HMBC spectrum of compound **4** in $CDCl_3$

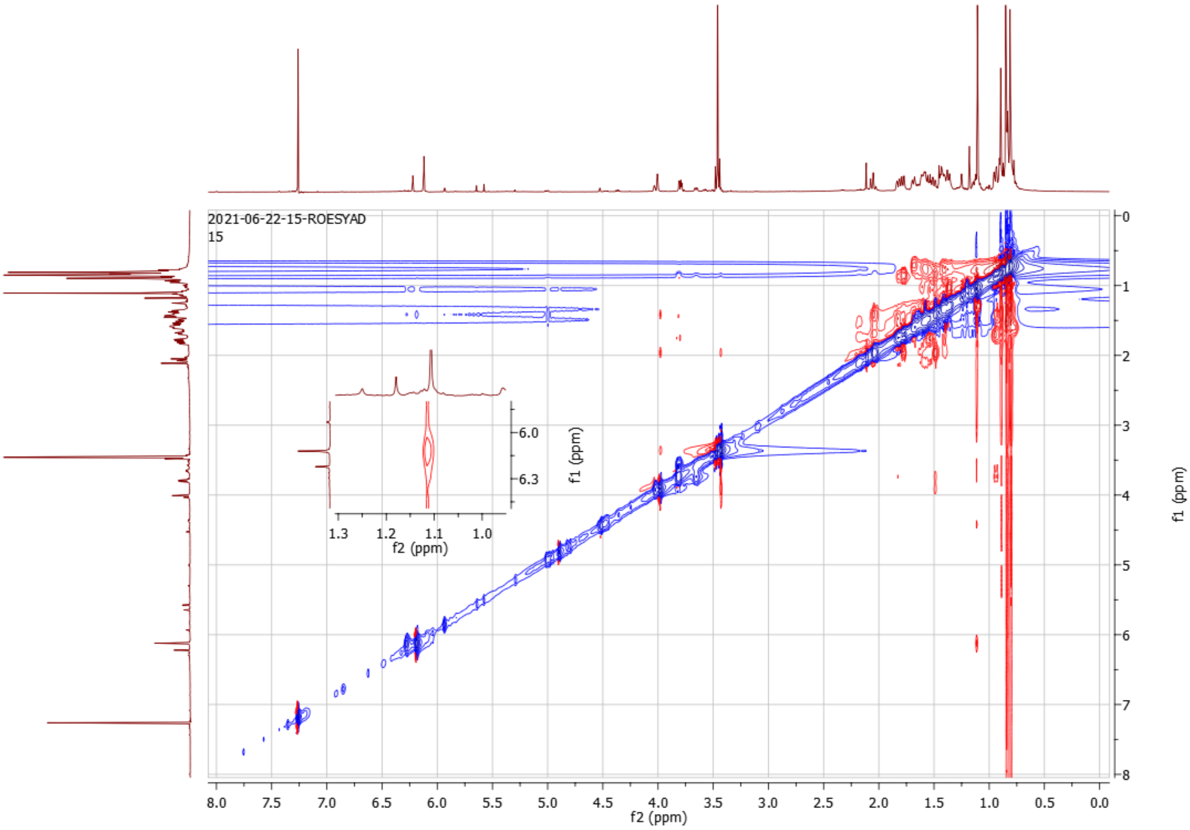

Fig. S.4.6. ROESY spectrum of compound **4** in $CDCl_3$

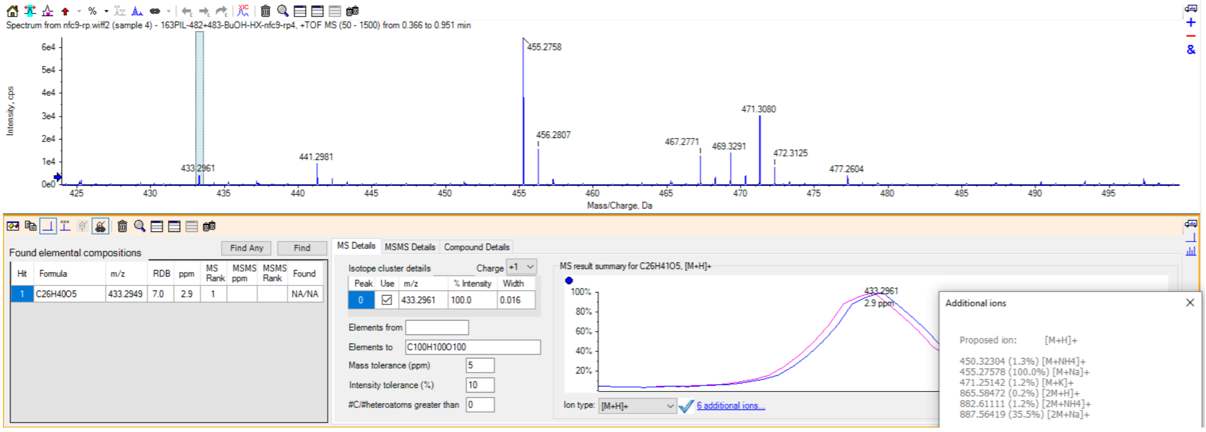

Fig. S.4.7. HR-ESIMS spectrum of compound **4**

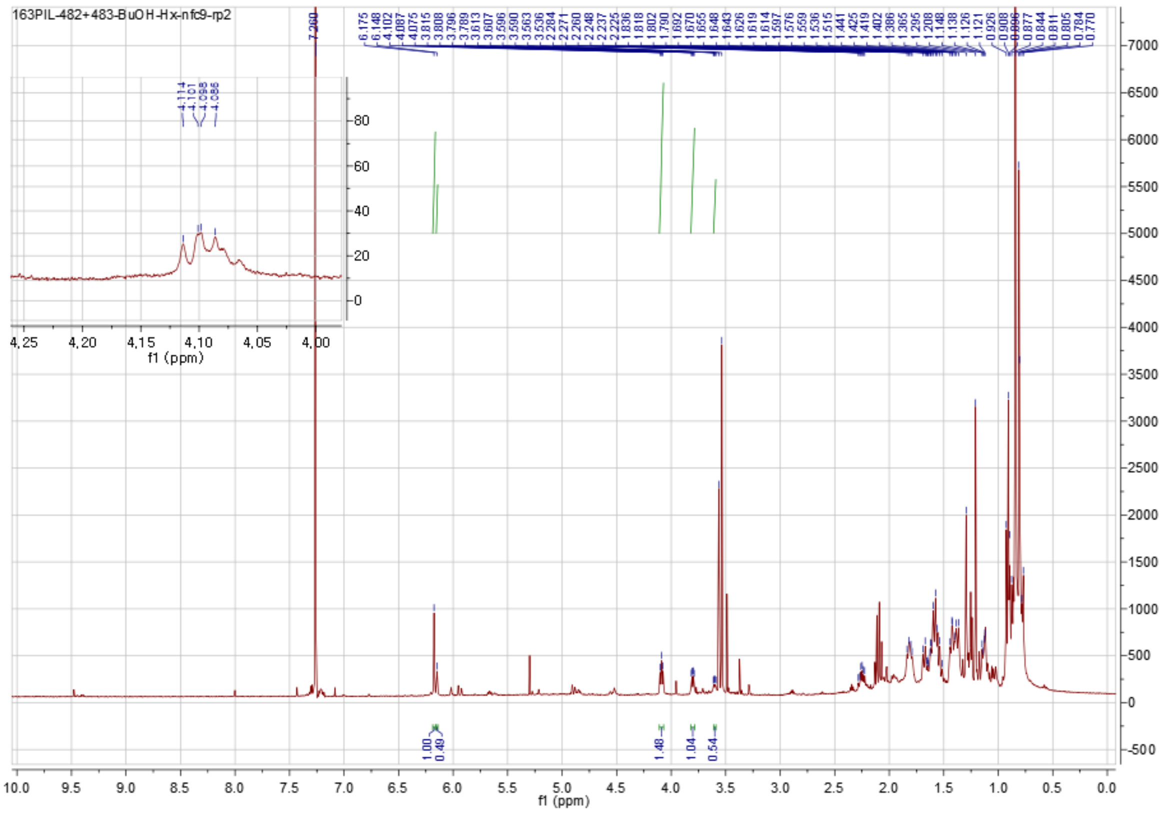

Fig. S.5.1. ^{1}H NMR spectrum of compound **5** in $CDCl_3$

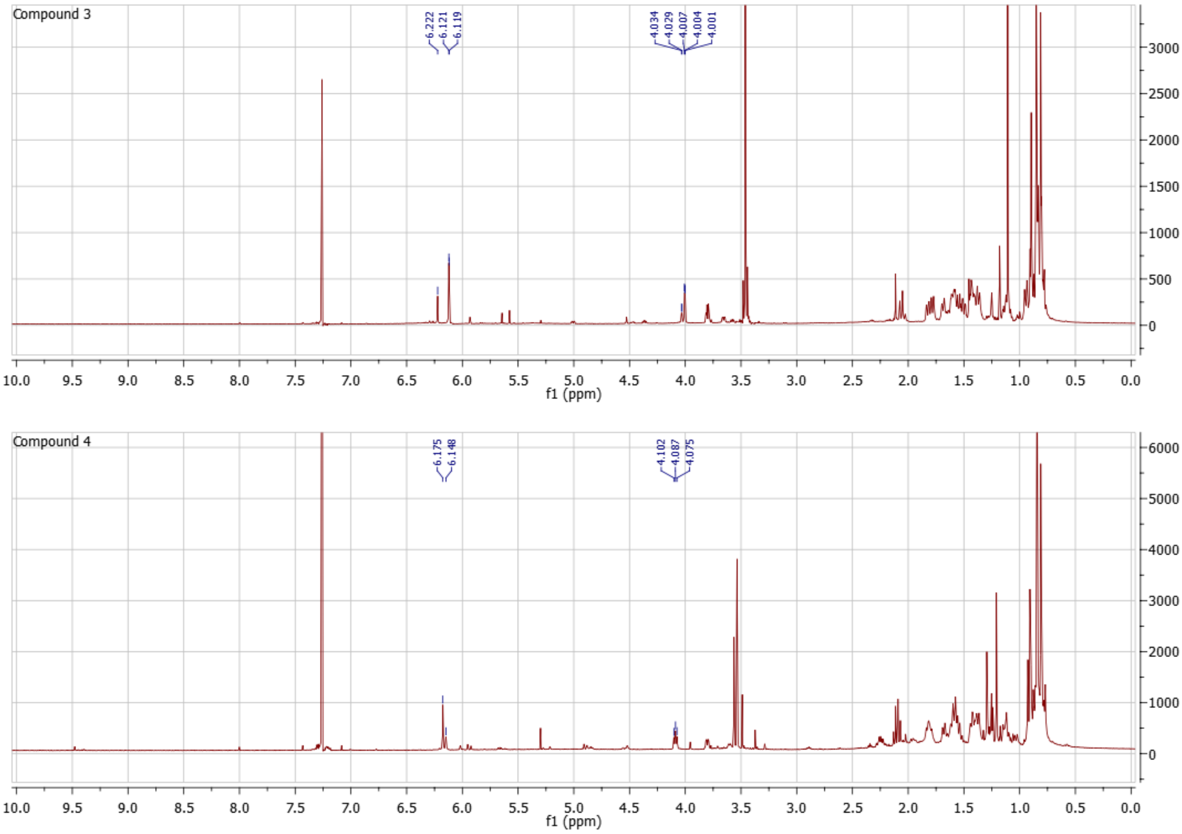

Fig. S.5.2. Comparison of ^{1}H NMR spectra between compound **4** and **5** in $CDCl_3$

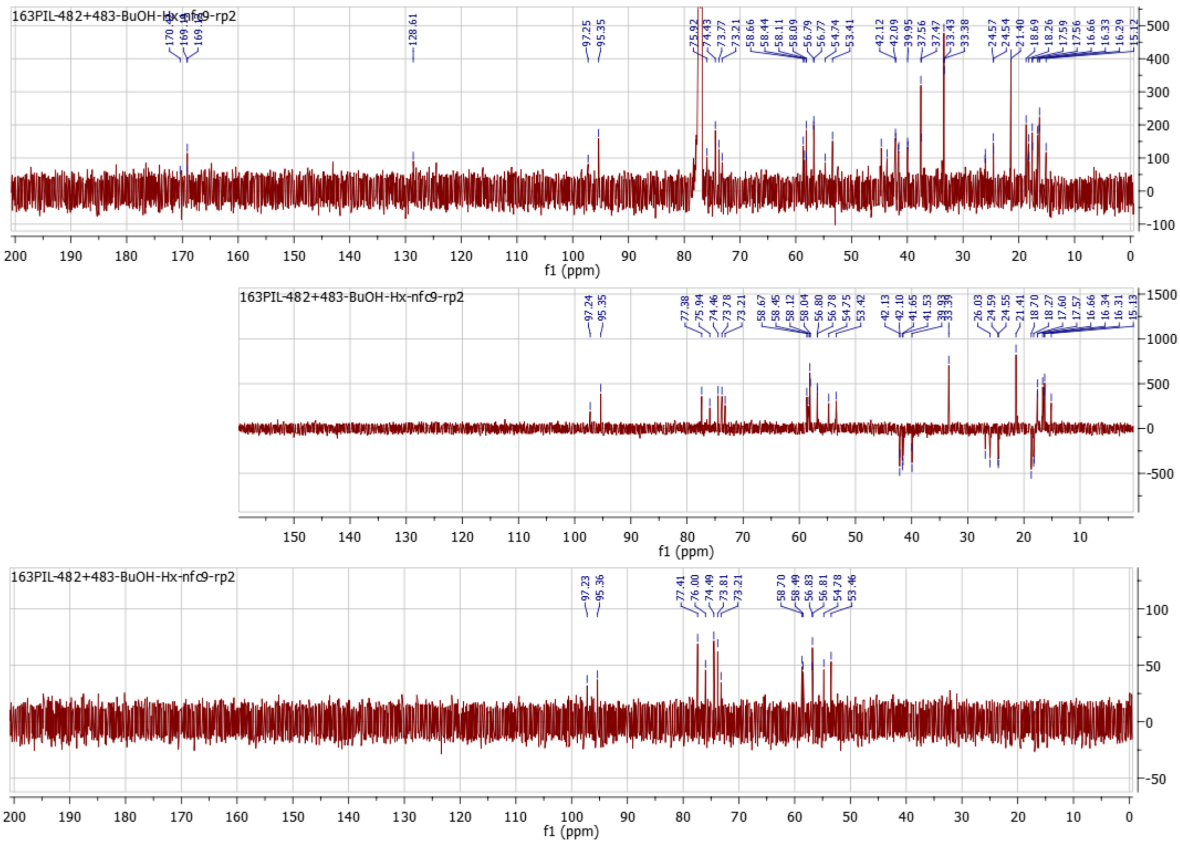

Fig. S.5.3. ^{13}C NMR and DEPT spectra of compound **5** in $CDCl_3$

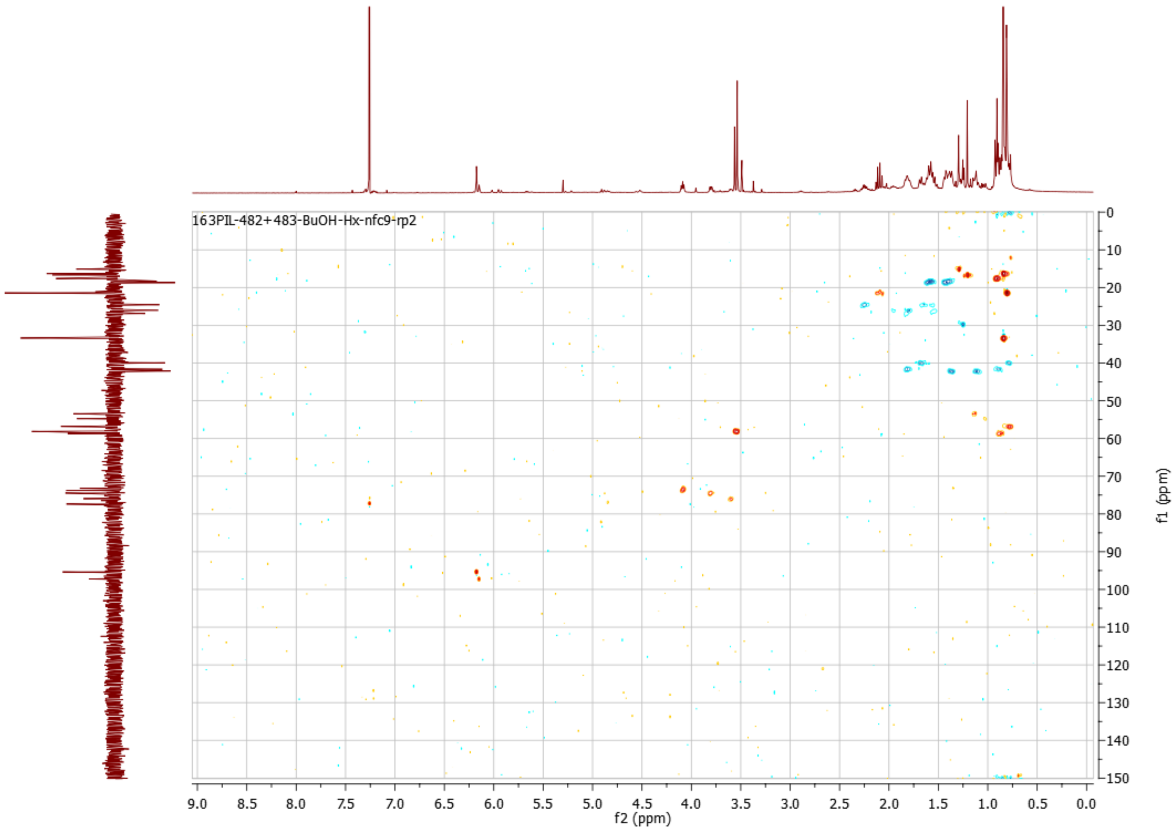

Fig. S.5.4. HSQC spectrum of compound **5** in $CDCl_3$

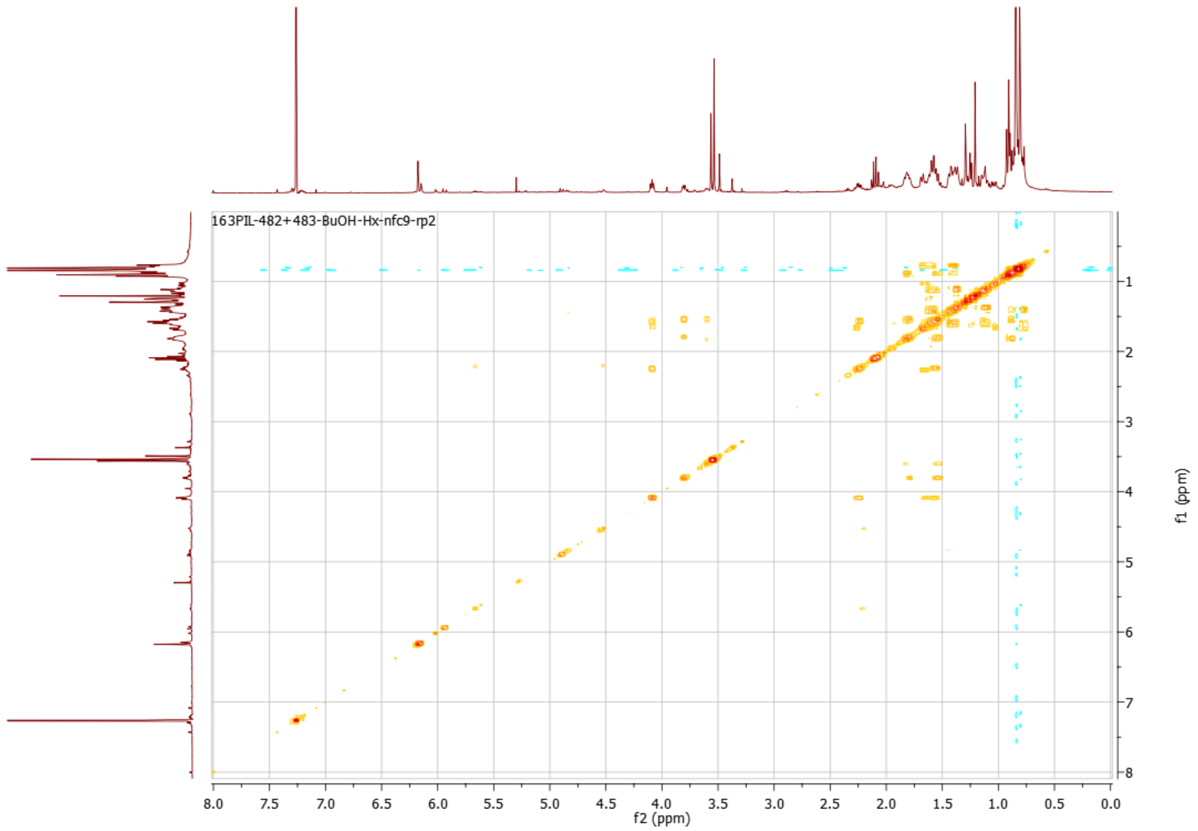

Fig. S.5.5. COSY spectrum of compound **5** in $CDCl_3$

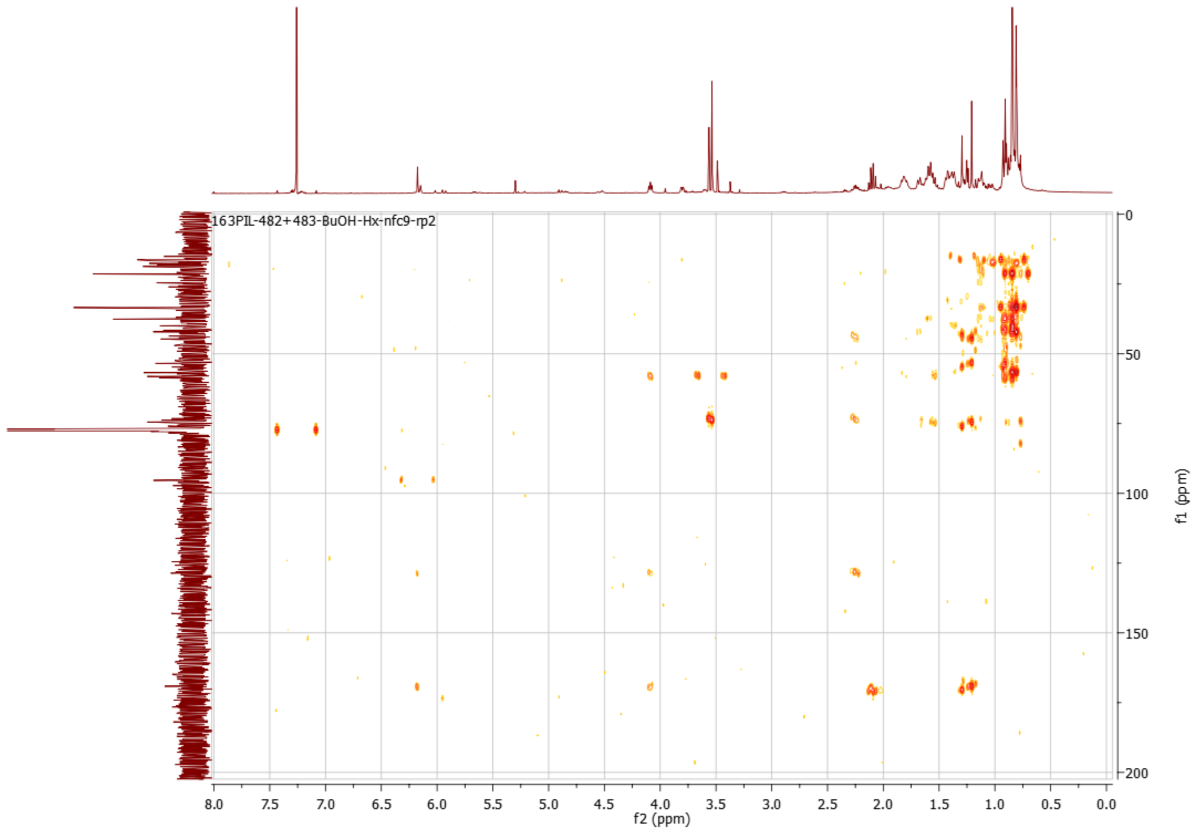

Fig. S.5.6. HMBC spectrum of compound **5** in $CDCl_3$

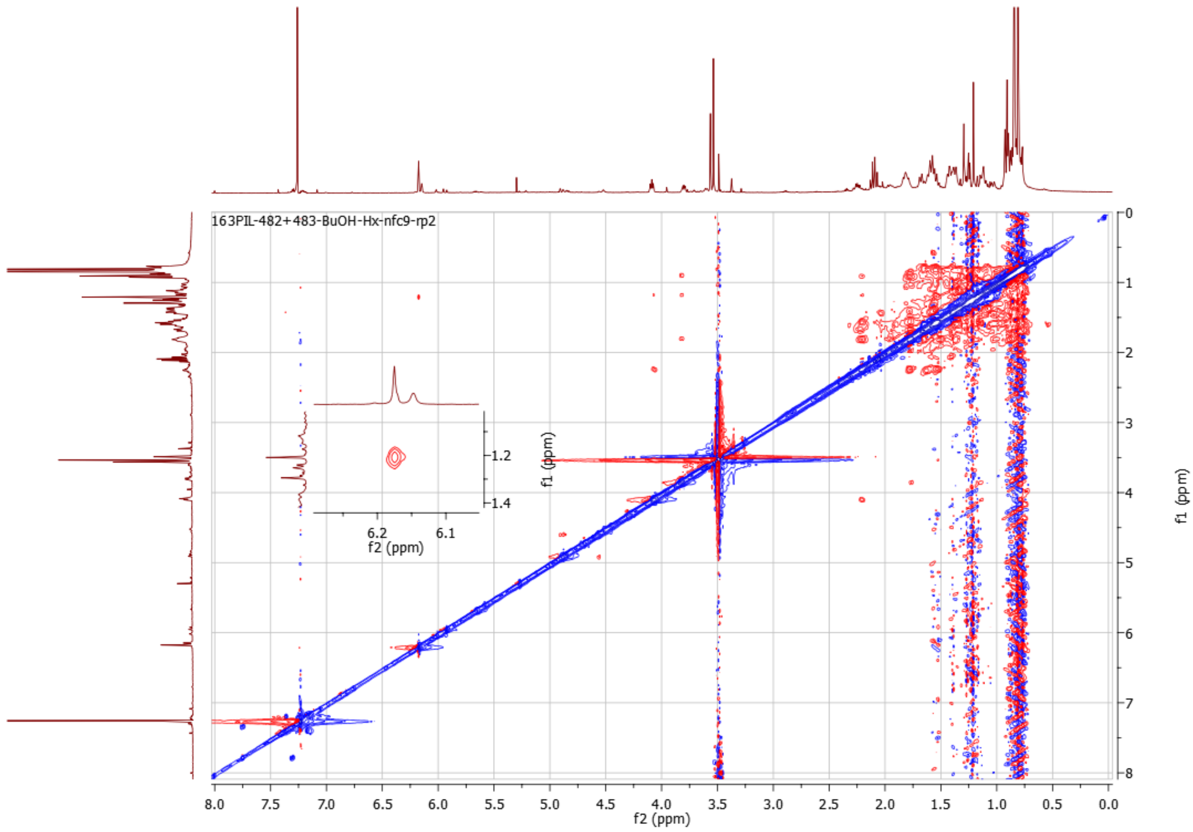

Fig. S.5.7. ROESY spectrum of compound **5** in $CDCl_3$

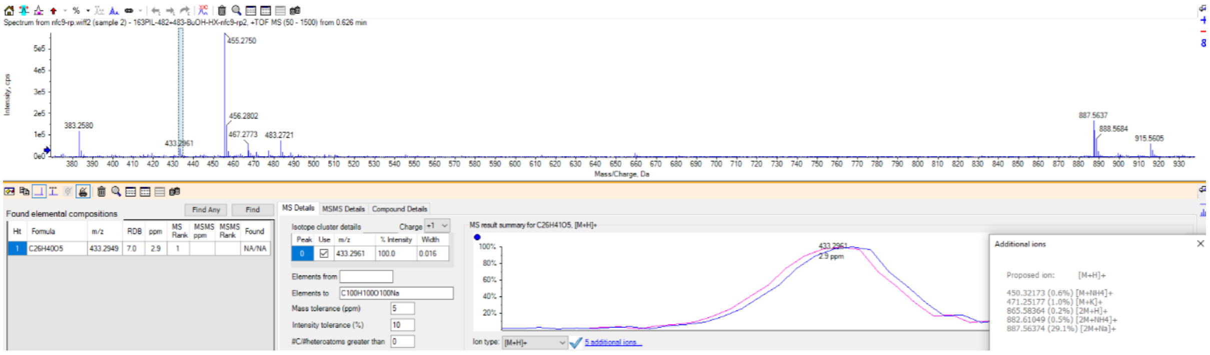

Fig. S.5.8. HR-ESIMS spectrum of compound **5**

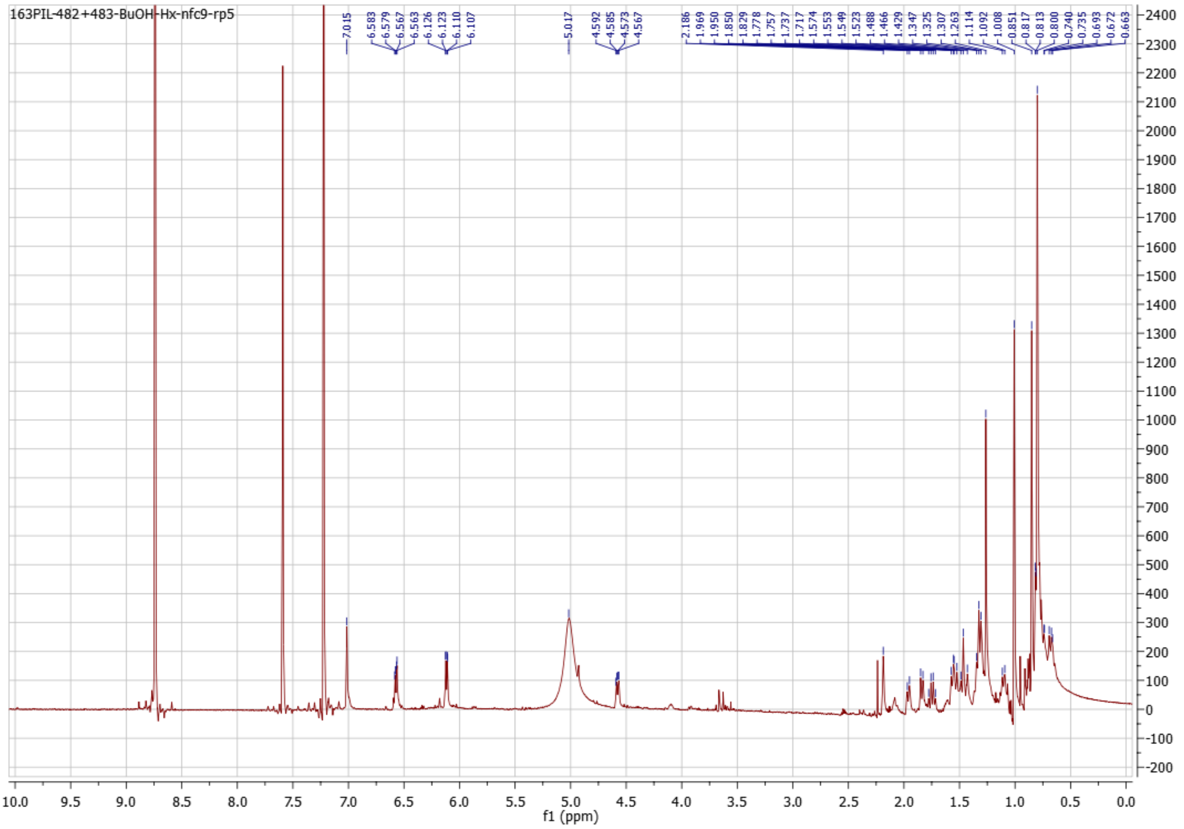

Fig. S.6.1. ^{1}H NMR spectrum of compound **6** in C_5D_5N

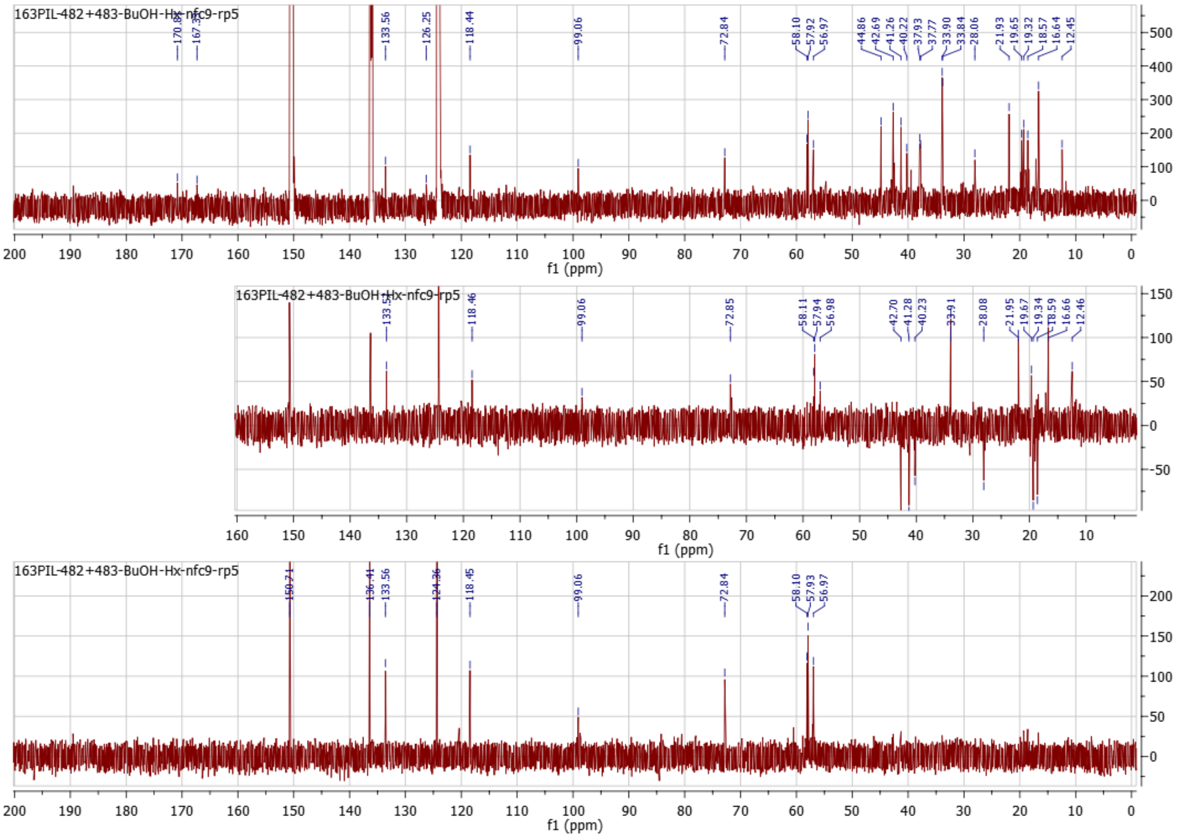

Fig. S.6.2. ^{13}C NMR and DEPT spectra of compound **6** in C_5D_5N

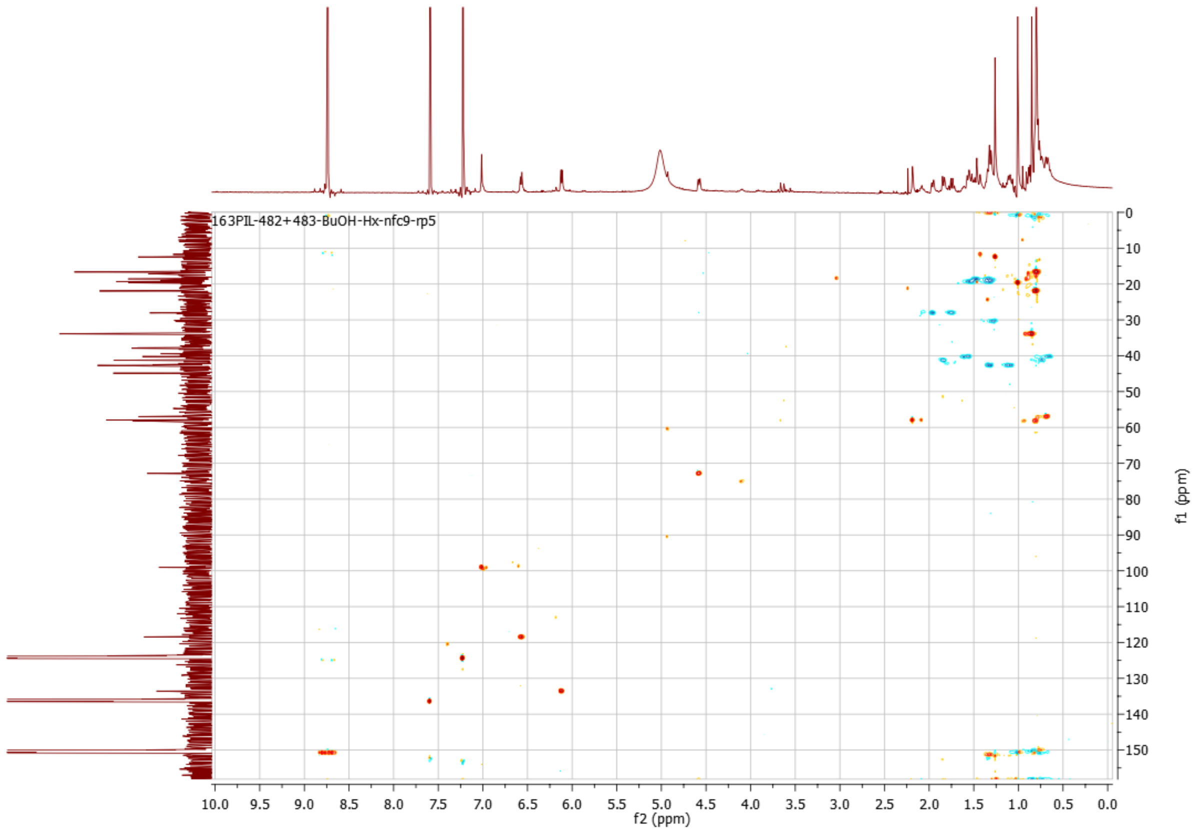

Fig. S.6.3. HSQC spectrum of compound **6** in C_5D_5N

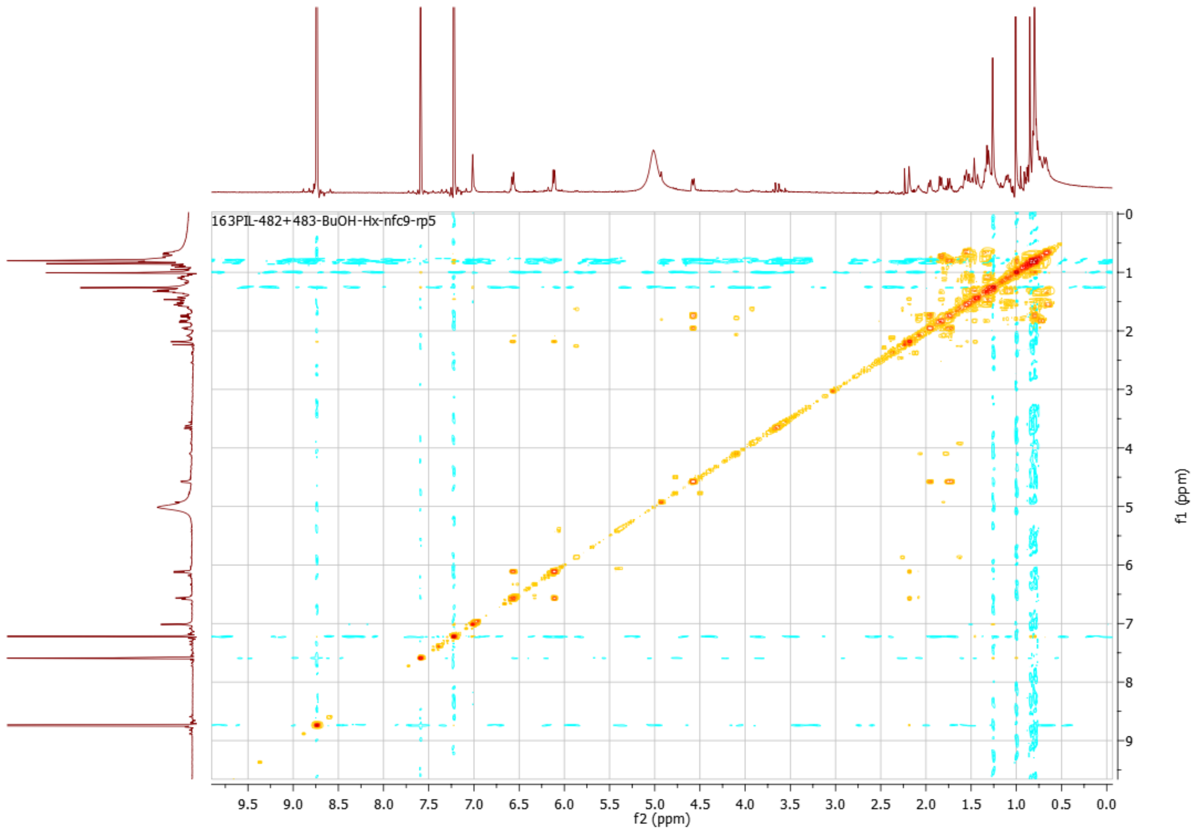

Fig. S.6.4. COSY spectrum of compound **6** in C_5D_5N

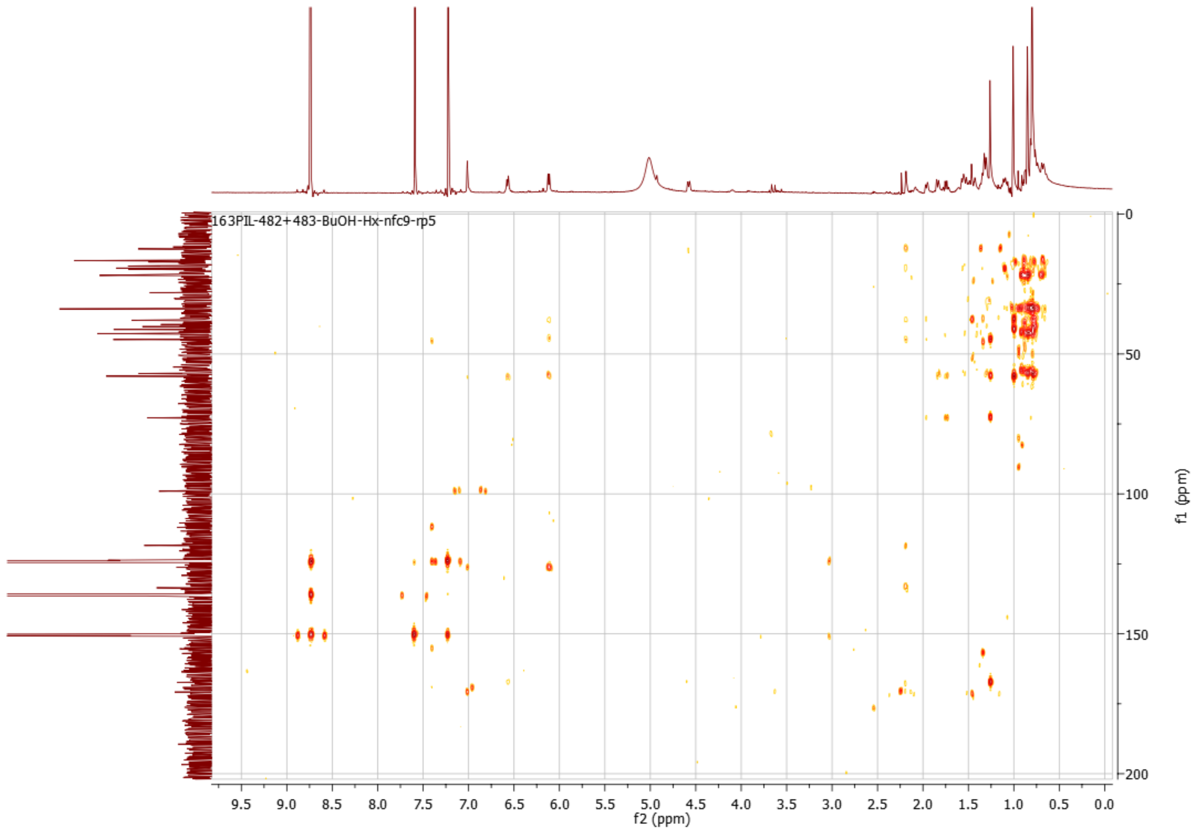

Fig. S.6.5. HMBC spectrum of compound **6** in C_5D_5N

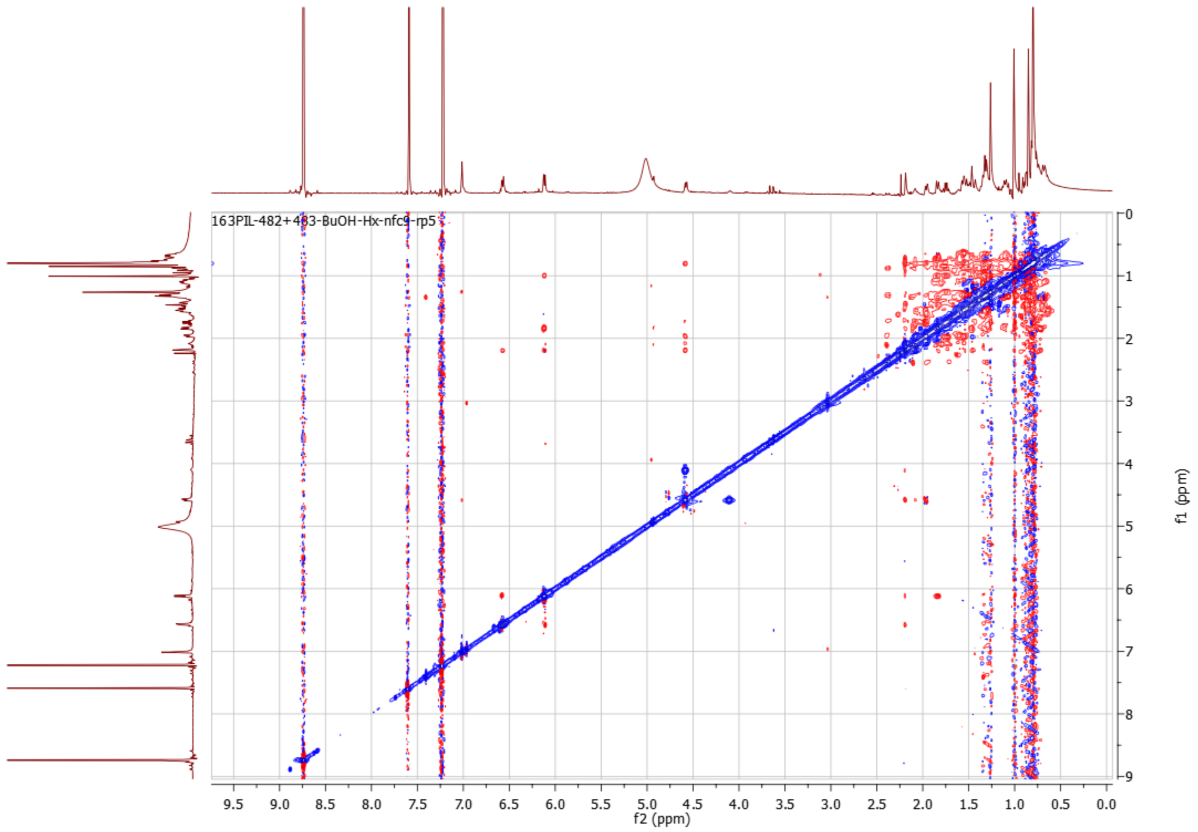

Fig. S.6.6. ROESY spectrum of compound **6** in C_5D_5N

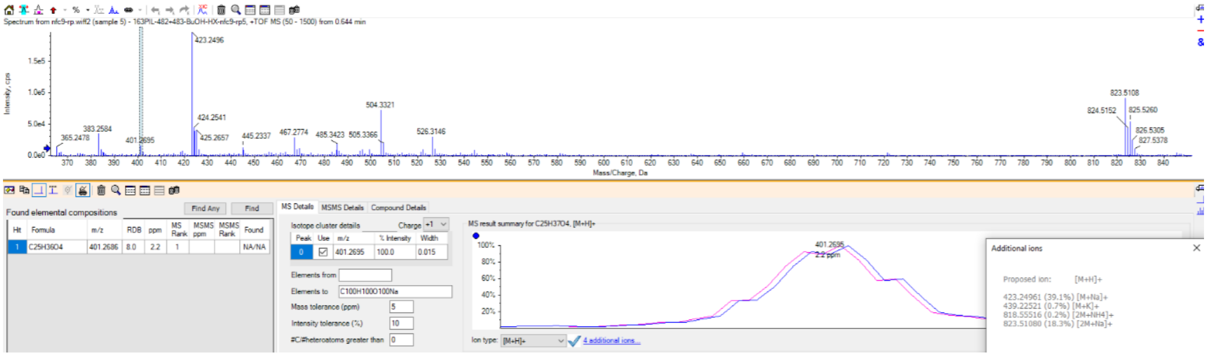

Fig. S.6.7. HR-ESIMS spectrum of compound **6**

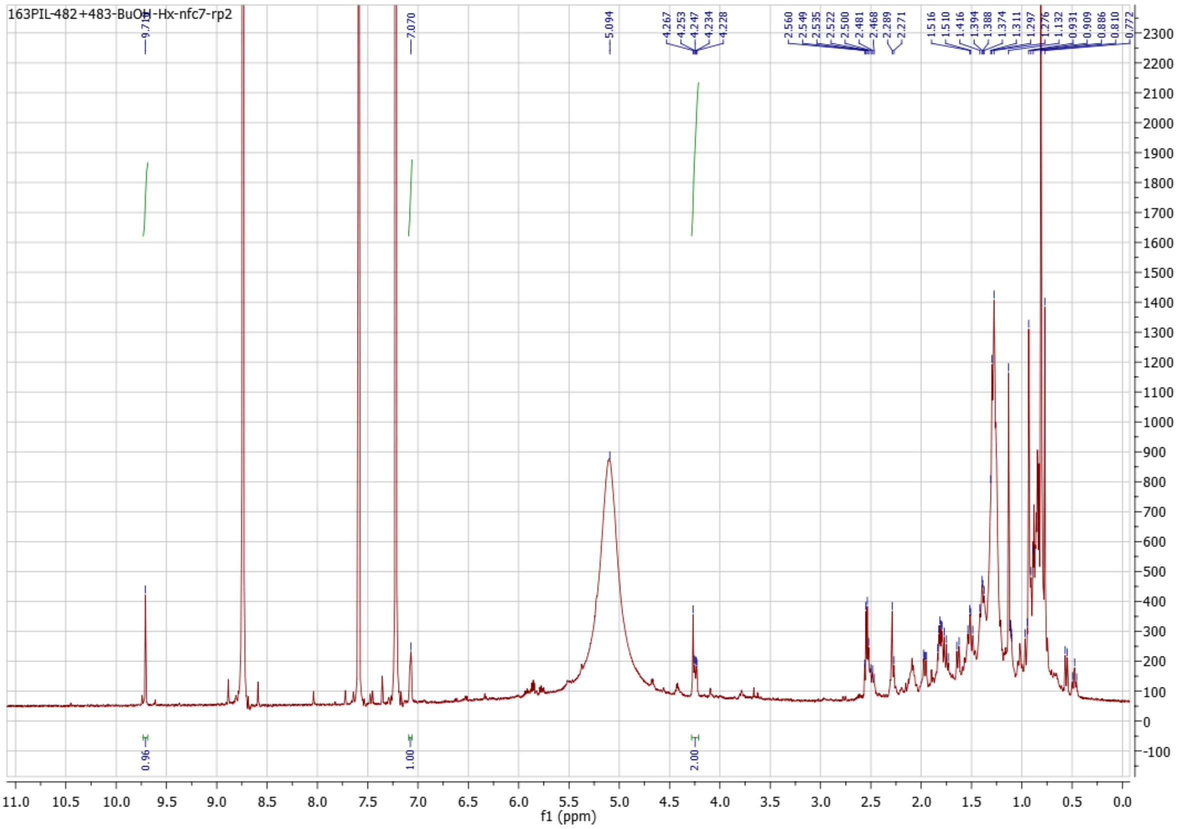

Fig. S.7.1. ^{1}H NMR spectrum of compound **7** in C_5D_5N

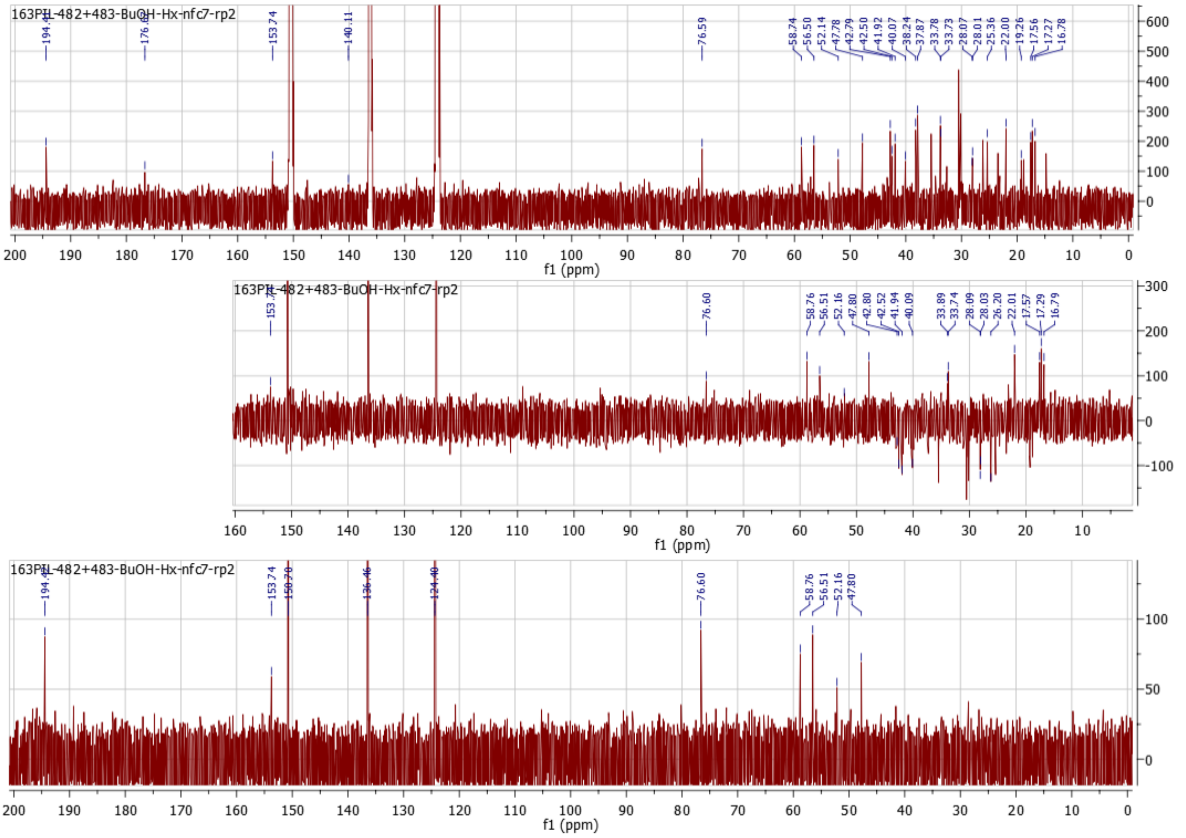

Fig. S.7.2. ^{13}C NMR and DEPT spectra of compound **7** in C_5D_5N

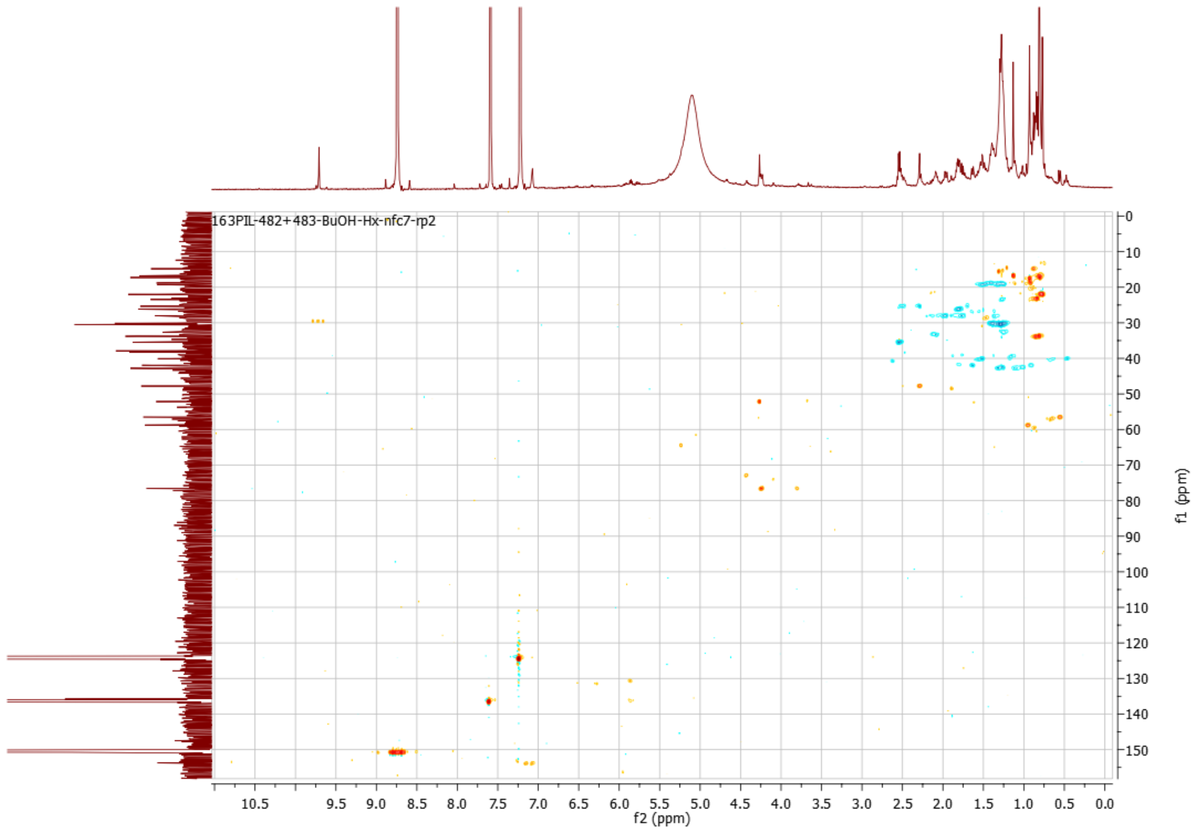

Fig. S.7.3. HSQC spectrum of compound **7** in C_5D_5N

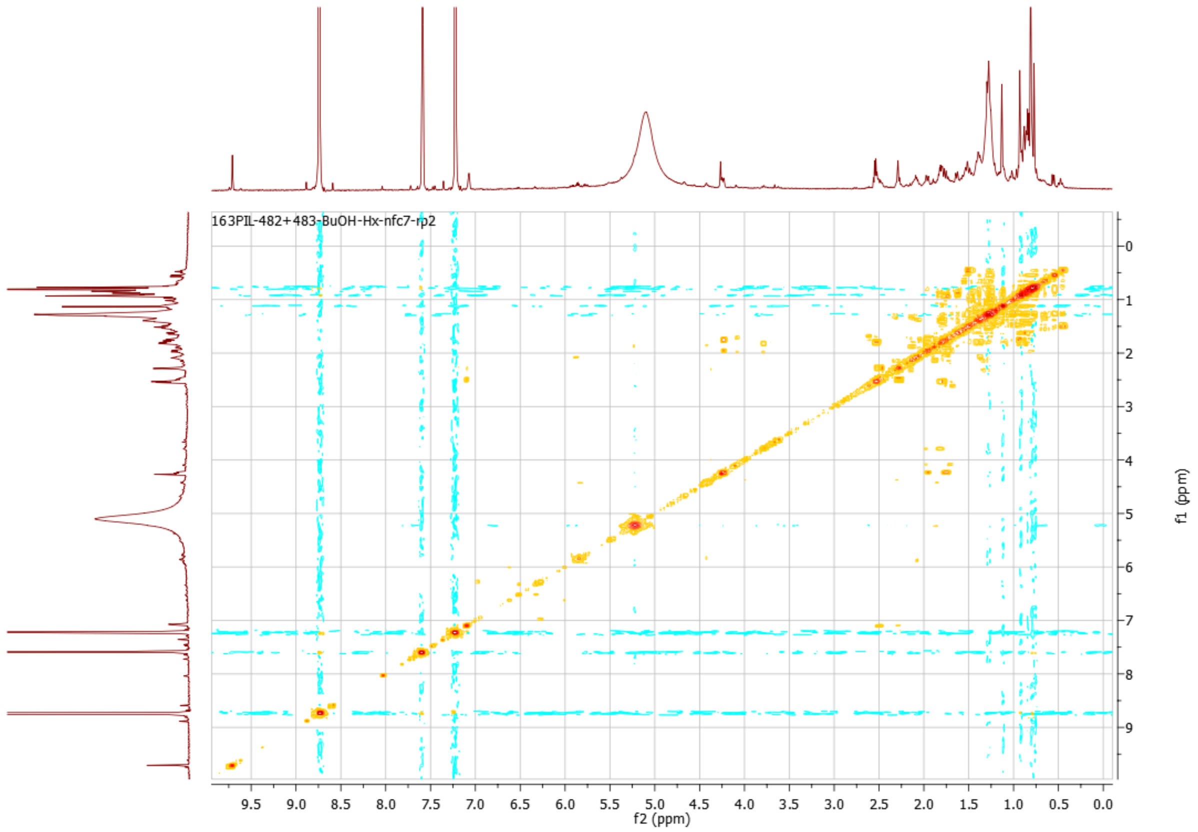

Fig. S.7.4. COSY spectrum of compound **7** in C_5D_5N

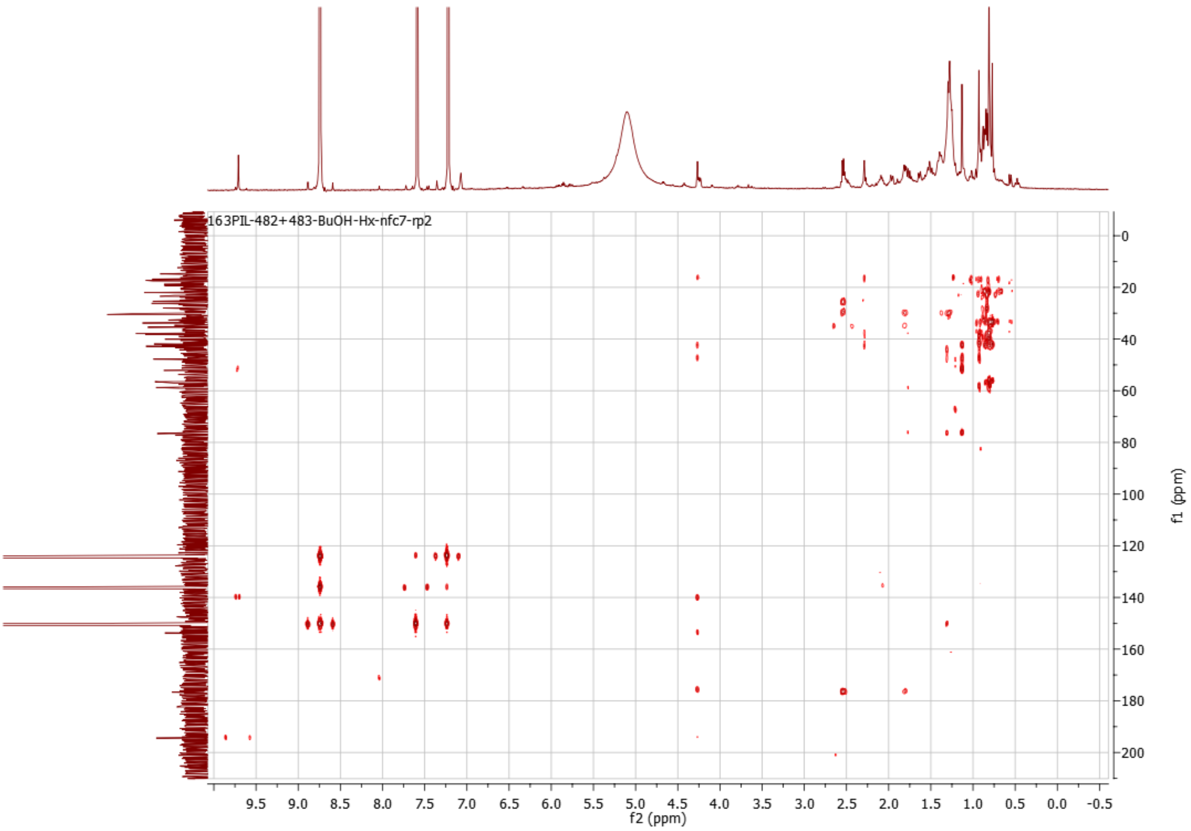

Fig. S.7.5. HMBC spectrum of compound **7** in C_5D_5N

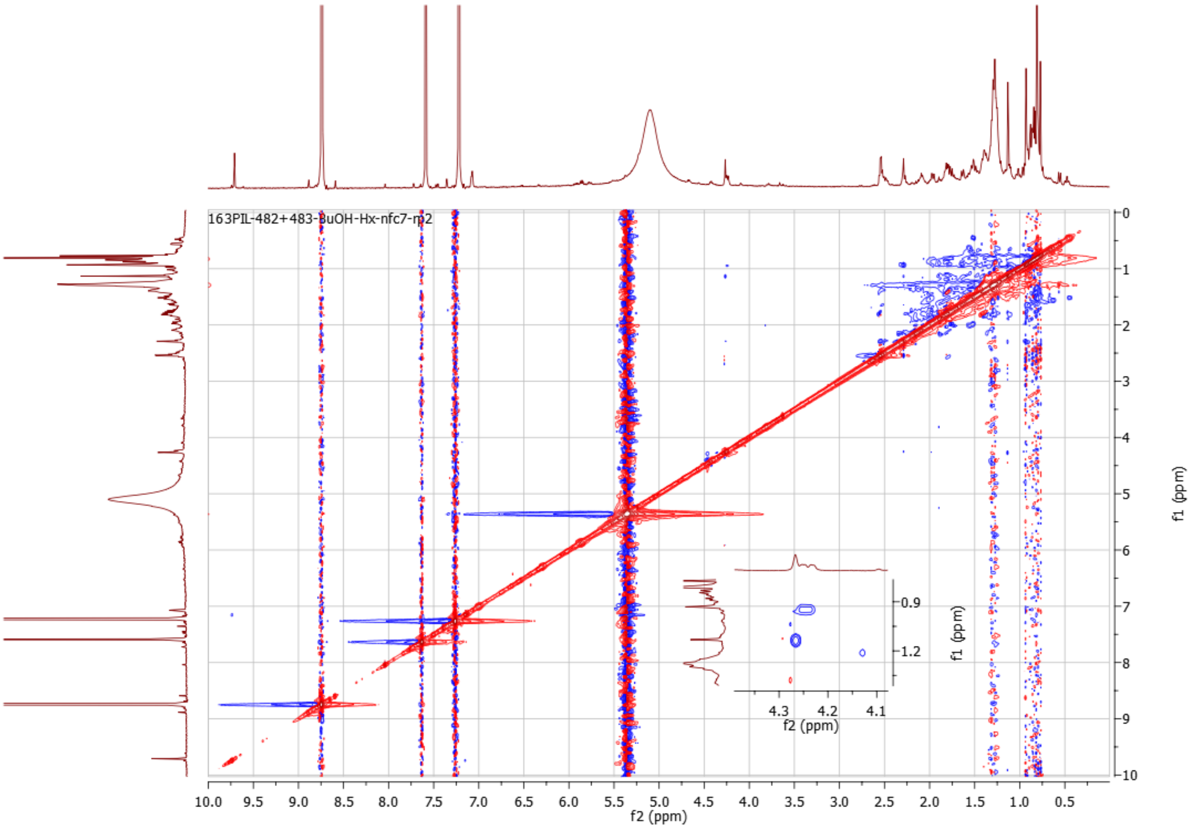

Fig. S.7.6. ROESY spectrum of compound **7** in C_5D_5N

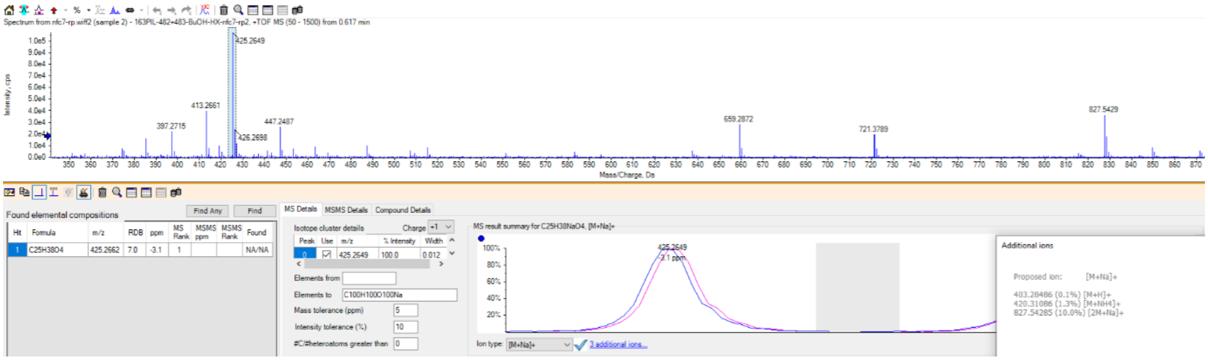

Fig. S.7.7. HR-ESIMS spectrum of compound **7**

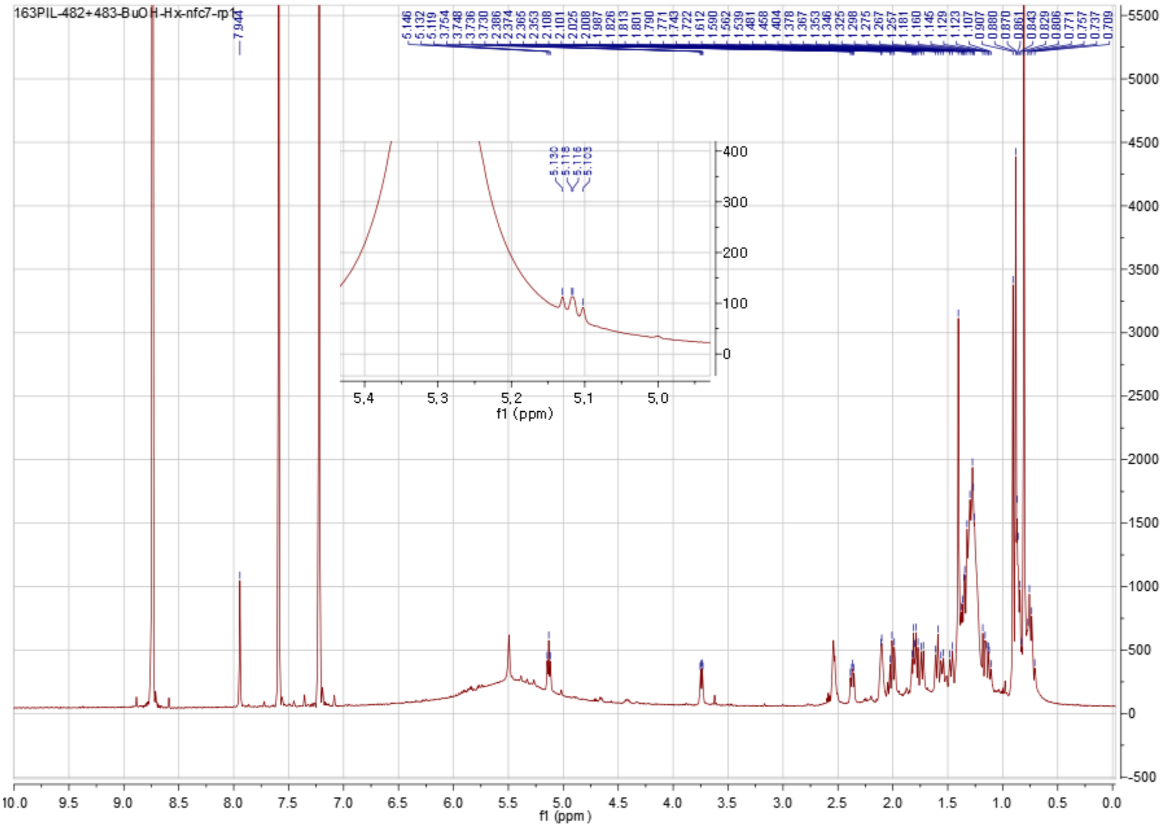

Fig. S.8.1. ^{1}H NMR spectrum of compound **8** in C_5D_5N

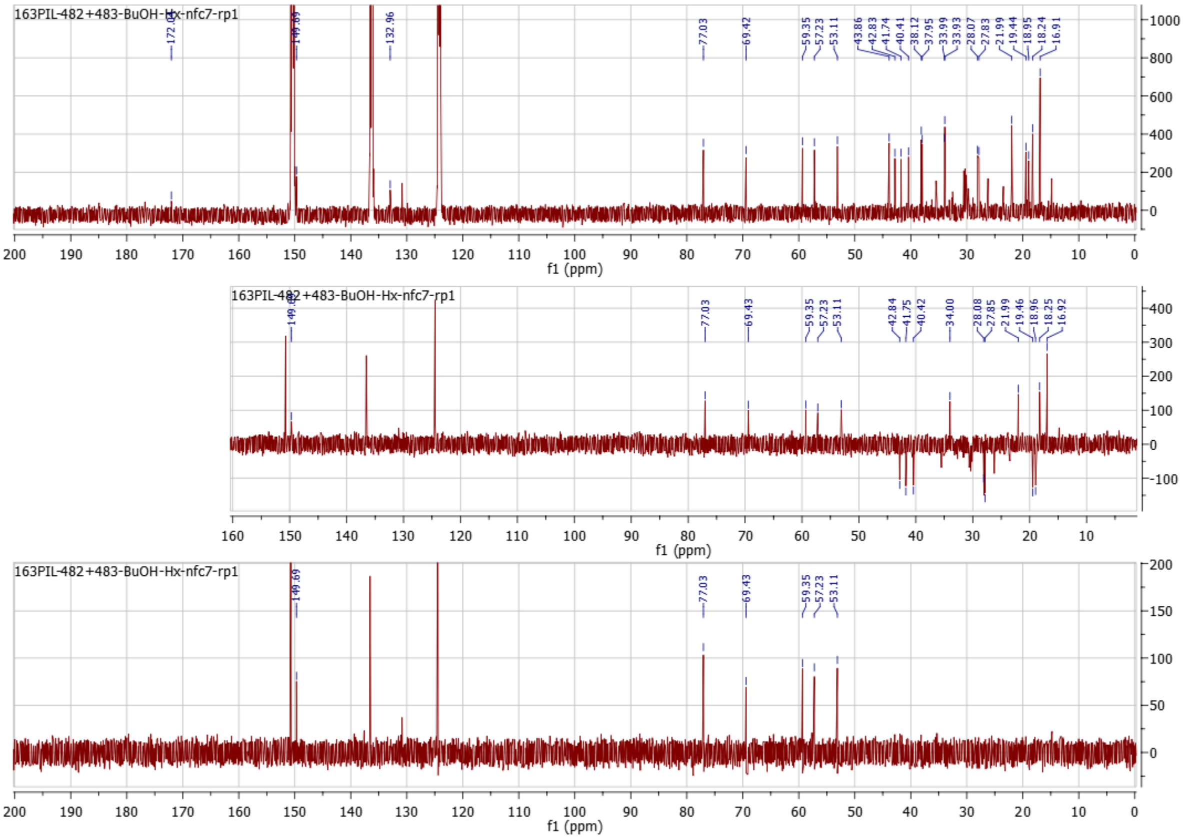

Fig. S.8.2. ^{13}C NMR and DEPT spectra of compound **8** in C_5D_5N

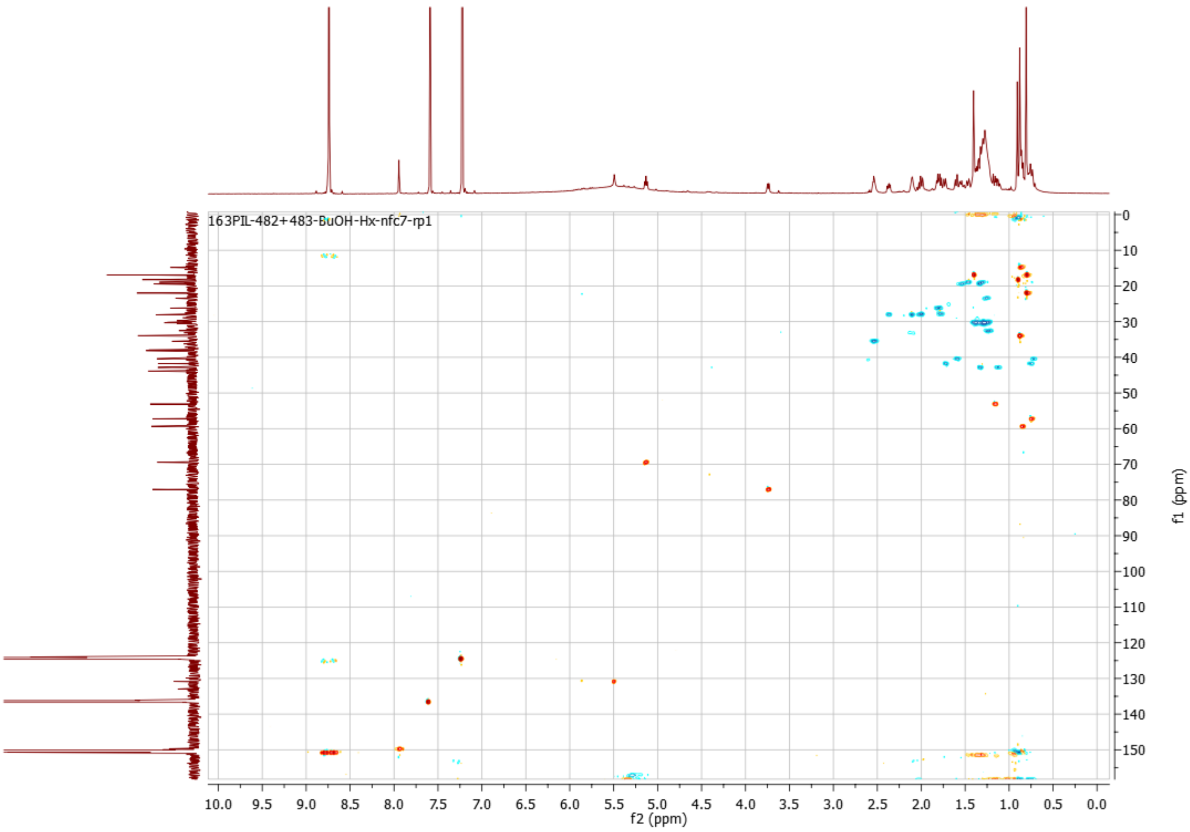

Fig. S.8.3. HSQC spectrum of compound **8** in C_5D_5N

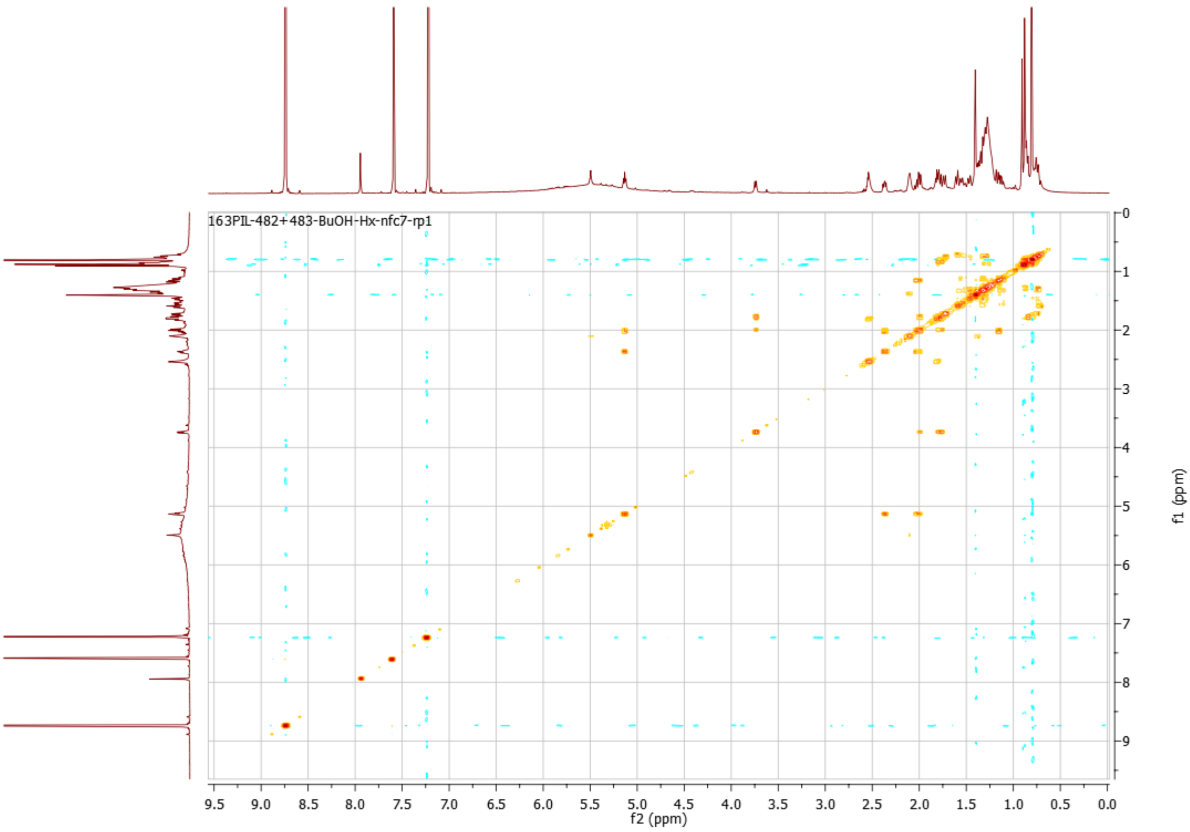

Fig. S.8.4. COSY spectrum of compound **8** in C_5D_5N

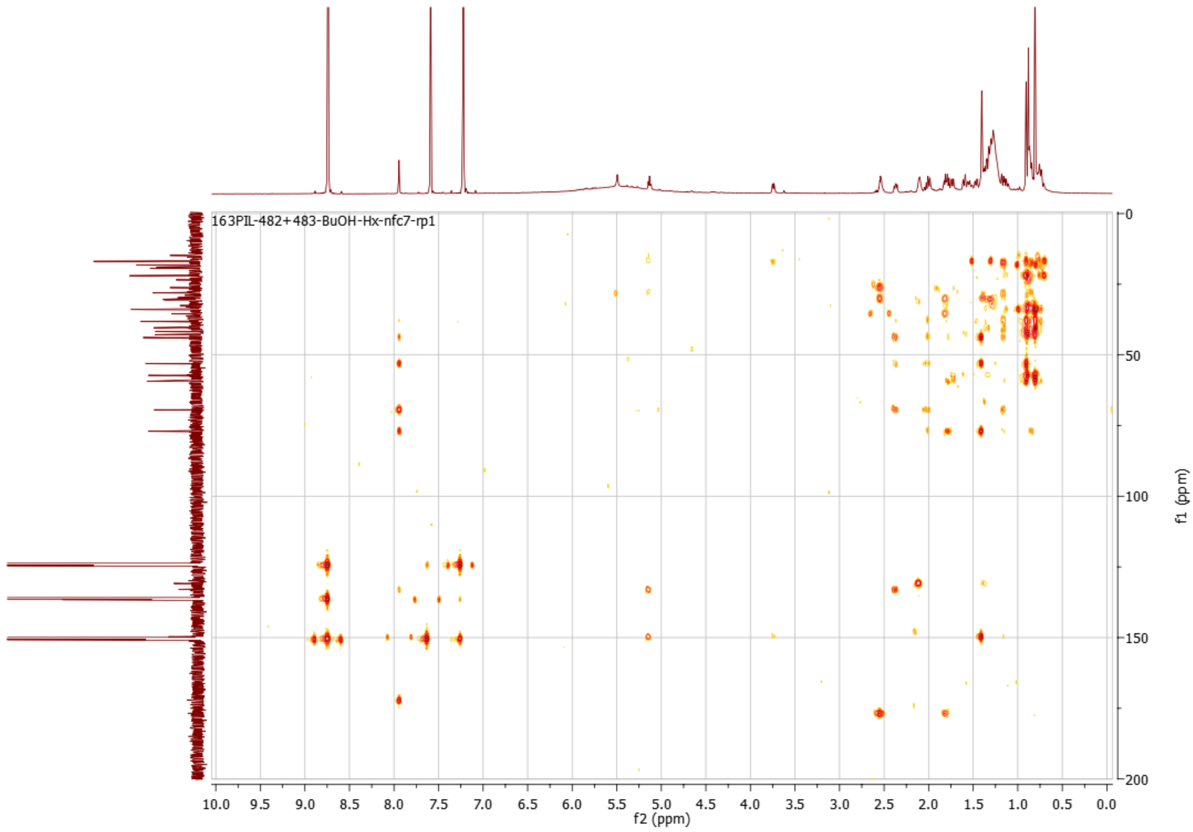

Fig. S.8.5. HMBC spectrum of compound **8** in C_5D_5N

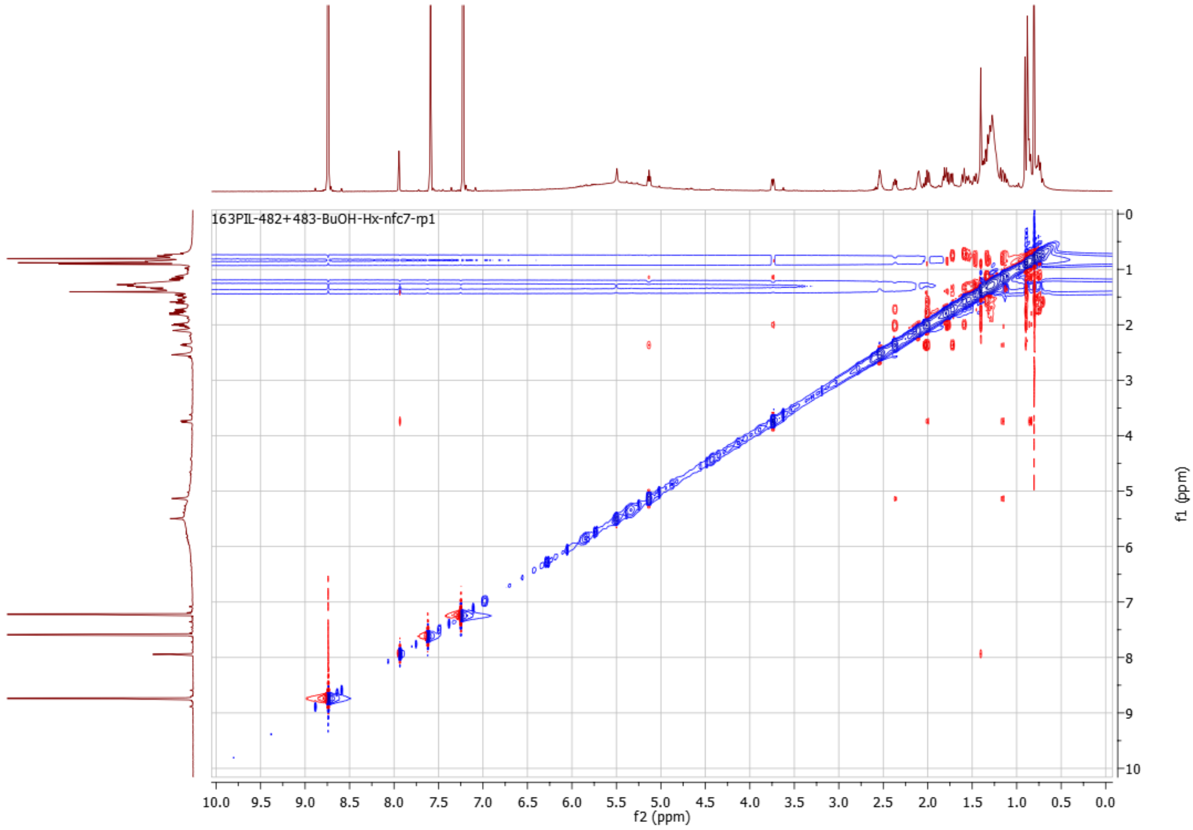

Fig. S.8.6. ROESY spectrum of compound **8** in C_5D_5N

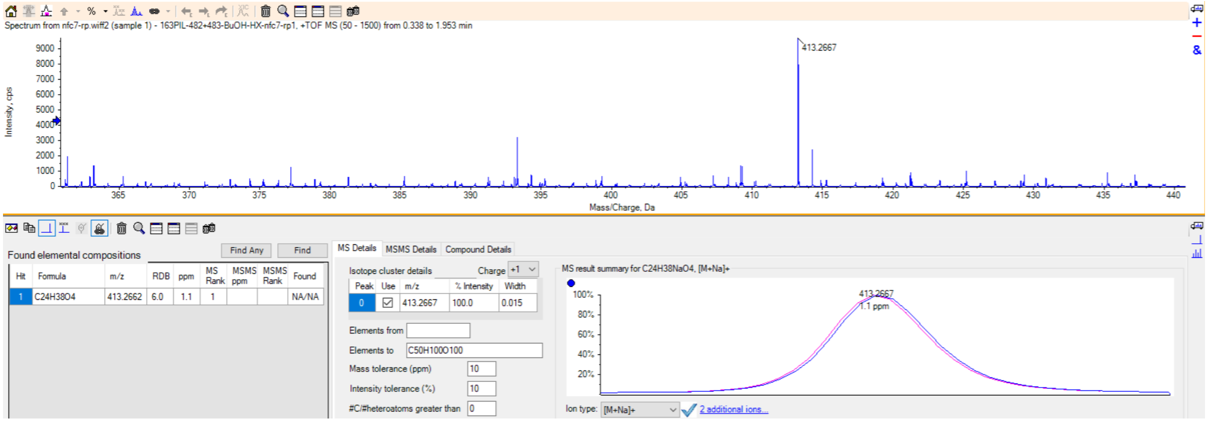

Fig. S.8.7. HR-ESIMS spectrum of compound **8**

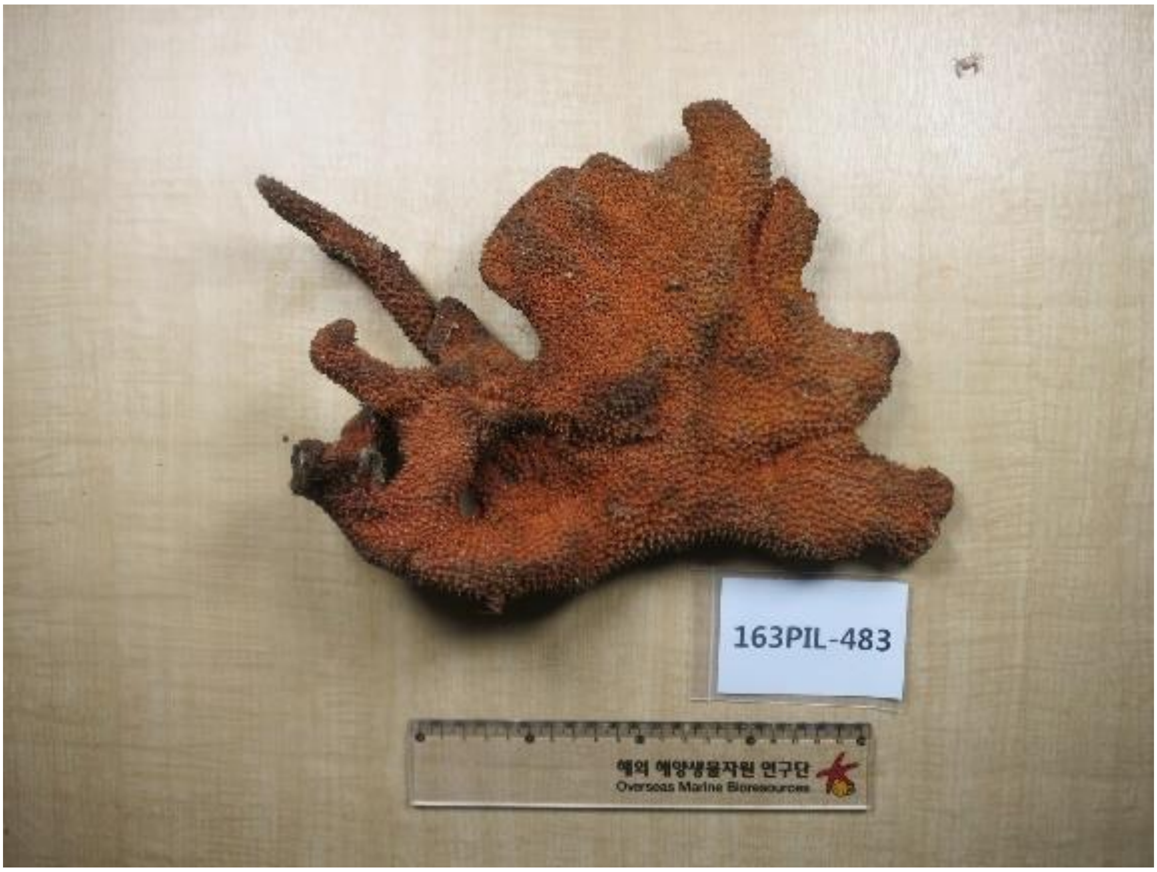

Fig. S.9. A photo of *Hyrtios erectus* used in this research
